# Genome-wide association study of myocardial infarction, atrial fibrillation, acute stroke, acute kidney injury and delirium after cardiac surgery – a sub-analysis of the RIPHeart-Study

**DOI:** 10.1186/s12872-019-1002-x

**Published:** 2019-01-24

**Authors:** Sabine Westphal, Christian Stoppe, Matthias Gruenewald, Berthold Bein, Jochen Renner, Jochen Cremer, Mark Coburn, Gereon Schaelte, Andreas Boening, Bernd Niemann, Frank Kletzin, Jan Roesner, Ulrich Strouhal, Christian Reyher, Rita Laufenberg-Feldmann, Marion Ferner, Ivo F. Brandes, Martin Bauer, Andreas Kortgen, Sebastian N. Stehr, Maria Wittmann, Georg Baumgarten, Rafael Struck, Tanja Meyer-Treschan, Peter Kienbaum, Matthias Heringlake, Julika Schoen, Michael Sander, Sascha Treskatsch, Thorsten Smul, Ewa Wolwender, Thomas Schilling, Frauke Degenhardt, Andre Franke, Soeren Mucha, Lukas Tittmann, Madeline Kohlhaas, Georg Fuernau, Oana Brosteanu, Dirk Hasenclever, Kai Zacharowski, Patrick Meybohm, RIPHeart-Study Collaborators, Ana Stevanovic, Rolf Rossaint, Marc Felzen, Andreas Goetzenich, Tobias Moormann, Katharina Chalk, Pascal Knuefermann, Olaf Boehm, Andreas Hoeft, Michael Winterhalter, Sonja Iken, Christian Weber, Carolin Wiedenbeck, Gerhard Schwarzmann, Karin Pense, Andreas Zierer Stephan Fichtlscherer, Gerold Goerlach, Matthias Wollbrueck, Ursula Boening, Markus Weigand, Julia Strauchmann, Konrad August, Kai U. Morsbach, Markus Paxian, Konrad Reinhard, Matthias Gruenewald, Jens Scholz, Ole Broch, Helga Francksen, Martin Albrecht, Bernd Kuhr, Hermann Heinze, Hauke Paarmann, Hans-Hinrich Sievers, Stefan Klotz, Thomas Hachenberg, Christian Werner, Susanne Mauff, Angela Alms, Stefan Bergt, Norbert Roewer

**Affiliations:** 1 Department of Anaesthesiology, Intensive Care Medicine and Pain Therapy, University Hospital Frankfurt, Frankfurt, Germany; 2 Department of Anaesthesiology, Medical Faculty, RWTH Aachen, University Aachen, Aachen, Germany; 3 Department of Anaesthesiology, Intensive Care Medicine, Emergency Medicine and Pain Therapy, Asklepios Klinik St. Georg, Hamburg, Germany; 4 Department of Anaesthesiology and Intensive Care Medicine, University Hospital Schleswig-Holstein, Campus Kiel, Kiel, Germany; 5 Department of Cardiovascular Surgery, University Hospital Schleswig-Holstein, Campus Kiel, Kiel, Germany; 6 Department of Cardiovascular Surgery, University of Giessen, Giessen, Germany; 7 Department of Anaesthesiology and Intensive Care, University of Giessen, Giessen, Germany; 8 Clinic of Anaesthesiology and Intensive Care Medicine, University Hospital Rostock, Rostock, Germany; 9 Department of Anaesthesiology and Intensive Care, Suedstadt Hospital Rostock, Rostock, Germany; 10 Department of Anesthesiology, Medical Center of Johannes Gutenberg-University, Mainz, Germany; 11 Department of Anaesthesiology and Intensive Care Medicine, University Hospital Goettingen, Goettingen, Germany; 12 Department of Anaesthesiology and Intensive Care, Klinikum Region Hannover, Hannover, Germany; 13 Department of Anaesthesiology and Intensive Care Medicine and Center for Sepsis Control and Care, Jena University Hospital, Jena, Germany; 14 Department of Anesthesiology and Intensive Care Medicine, University Hospital Leipzig, Leipzig, Germany; 15 Department of Anaesthesiology and Intensive Care Medicine, University Hospital Bonn, Bonn, Germany; 16 Department of Anaesthesiology and Intensive Care Medicine, Johanniter Hospital Bonn, Bonn, Germany; 17 Department of Anaesthesiology and Intensive Care Medicine, University Hospital Duesseldorf, Duesseldorf, Germany; 18 Department of Anaesthesiology and Intensive Care Medicine, University Luebeck, Luebeck, Germany; 19 Department of Anaesthesiology and Intensive Care Medicine, Hospital Neuruppin, Neuruppin, Germany; 20 Department of Anaesthesiology and Intensive Care Medicine, Charité-Universitätsmedizin Berlin, Campus Charité Mitte, Berlin, Germany; 21 Department of Anaesthesiology, University Hospital Wuerzburg, Wuerzburg, Germany; 22 Institute of Clinical Molecular Biology, Kiel University, University Hospital Schleswig-Holstein, Campus Kiel, Kiel, Germany; 23 University Heart Center Luebeck, Medical Clinic II (Cardiology/Angiology/Intensive Care Medicine), University Hospital Schleswig-Holstein, Luebeck, Luebeck, Germany; 24 Clinical Trial Centre, University Leipzig, Leipzig, Germany; 25 Institute for Medical Informatics, Statistics and Epidemiology, University Leipzig, Leipzig, Germany

**Keywords:** Genome-wide association study, Cardiac surgery, Atrial fibrillation, Delirium, Myocardial infarction, Acute kidney injury, Stroke

## Abstract

**Background:**

The aim of our study was the identification of genetic variants associated with postoperative complications after cardiac surgery.

**Methods:**

We conducted a prospective, double-blind, multicenter, randomized trial (RIPHeart). We performed a genome-wide association study (GWAS) in 1170 patients of both genders (871 males, 299 females) from the RIPHeart-Study cohort. Patients undergoing non-emergent cardiac surgery were included. Primary endpoint comprises a binary composite complication rate covering atrial fibrillation, delirium, non-fatal myocardial infarction, acute renal failure and/or any new stroke until hospital discharge with a maximum of fourteen days after surgery.

**Results:**

A total of 547,644 genotyped markers were available for analysis. Following quality control and adjustment for clinical covariate, one SNP reached genome-wide significance (*PHLPP2*, rs78064607, *p* = 3.77 × 10^− 8^) and 139 (adjusted for all other outcomes) SNPs showed promising association with *p* < 1 × 10^− 5^ from the GWAS.

**Conclusions:**

We identified several potential loci, in particular *PHLPP2*, *BBS9*, *RyR2*, *DUSP4* and *HSPA8*, associated with new-onset of atrial fibrillation, delirium, myocardial infarction, acute kidney injury and stroke after cardiac surgery.

**Trial registration:**

The study was registered with ClinicalTrials.gov NCT01067703, prospectively registered on 11 Feb 2010.

**Electronic supplementary material:**

The online version of this article (10.1186/s12872-019-1002-x) contains supplementary material, which is available to authorized users.

## Background

Coronary heart disease is the leading cause of death and disability worldwide and is responsible for about 7.2 million deaths every year. Cardiac surgery is one of the most common cardiac procedures, performed annually in about 1.5 million patients worldwide. In spite of advances in surgical techniques, the incidence of complications after cardiac surgery using cardiopulmonary bypass is still high. The most common complications following cardiac surgery are atrial fibrillation (AF), delirium, myocardial infarction (MI), acute renal failure and stroke, all of which increase mortality and lead to a prolonged stay on intensive care unit (ICU) of patients. The restoration of regional blood flow after a period of ischemia during cardiac surgery frequently causes further cellular organ injury and thereby potentially limiting the recovery of function. Reperfusion is often associated with microvascular dysfunction. Activated endothelial cells produce excessive reactive oxygen species (ROS), but less nitric oxide, leading to release of inflammatory mediators, mitochondrial dysfunction, oxidative stress and finally cell death [1].

Yet, the inflammatory mediators released as a consequence of reperfusion also appear to activate endothelial cells in remote organs not initially exposed to the ischemic stress, i.e. the kidney and the central nervous system. This distant response to ischemia/ reperfusion (I/R) can result in leukocyte-dependent microvascular injury that is characteristic of the “systemic inflammatory response syndrome”, potentially leading to delirium, stroke and acute kidney dysfunction [2].

Hypercholesterolemia, diabetes and hypertension, the occurrence and extent of complications after cardiac surgery could have a genetic basis. A genetic bias is strongly suggested by observations that the wide variability concerning incidence and severity of complications after cardiac surgery could not be explained by clinical or interventional risks. As postoperative organ dysfunction is common after cardiac surgery, several previous studies have performed preoperative genomic characterization of patients to identify genotypes, which render the patients vulnerable for the development of a specific organ dysfunction. Kertai et al. identified genetic variants in patients exhibiting AF and MI after cardiac surgery [3, 4]. Another recent study reported genetic variants concerning acute kidney injury after cardiac surgery [5]. All of these studies investigated one or two complications after cardiac surgery but none examined the complications in all, nor investigated a composite of all main complications. Therefore, we conducted a comprehensive genome-wide association study (GWAS) to identify common genetic variants associated with the main complications after cardiac surgery as new-onset postoperative AF, MI, delirium, stroke and acute renal failure.

## Methods

We performed a GWAS using in total 1170 DNA samples from the RIPHeart (Remote Ischemic Preconditioning for Heart Surgery) study cohort [6, 7] (871 males, 299 females) in the dataset testing 547,644 variants to identify candidate genes that predetermine main complications after cardiac surgery. Various samples were excluded for the different subanalyses. See below (statistics) for more information.

The initial objective of our prospective, double-blind, multicentre, randomized controlled RIPHeart study was to investigate whether upper limb remote ischemic preconditioning compared to sham intervention reduced the incidence of the primary endpoint including death, MI, stroke, and acute renal failure until hospital discharge in adults scheduled for elective cardiac surgery requiring cardiopulmonary bypass (for further information please read the english synopsis and study protocol of the RIPHeart study in Additional file 1: Figure S3 and Additional file 2: Figure S4 in the supplementary). As the initial intervention study did not show any group differences, this predefined secondary analysis of genome-wide association now includes all patients irrespective of the initial group assignment.

### Patient populations

The cohort comprised 1204 patients who underwent an elective cardiac surgery requiring cardiopulmonary bypass (e.g. coronary artery bypass graft, valve surgery, ascending aorta replacement) between January 2011 and May 2014 and were analysed. 1170 (871 males, 299 females) patients met eligibility criteria after applying quality control and excluding patients with missing genotypes or phenotypic information.

### Outcome measures

In this GWAS-study, the primary endpoint comprises a composite complication rate covering AF, delirium, MI, acute renal failure, and/or any new stroke. Non-fatal myocardial infarction was defined by

- biomarker values more than five times the 99th percentile of the normal reference range combined with new pathological Q-waves
- or new left bundle branch block (LBBB) within the first 72 h
- standard clinical criteria for myocardial infarction from 72 h on
- new ischemic finding by echocardiography/angiography
- or myocardial infarction diagnosed at autopsy.

Some patients present with ST elevation or new LBBB, and suffer sudden cardiac death before cardiac biomarkers become abnormal or pathological signs of myocardial necrosis become evident at autopsy. These patients should be classified as having had a fatal myocardial infarction [8].

A blinded clinical endpoint committee assessed all available electrocardiograms for reference reading. Stroke was defined by any new, temporary or permanent, focal or global neurological deficit, or evidence of stroke on autopsy, and was evaluated according to the National Institutes of Health Stroke Scale (≥ 4 points) [9]. Acute renal failure was defined by any serum creatinine greater than or equal to two-fold increase from baseline, urine output ≤0.5 mL/kg/h for 12 h [10], use of renal replacement therapy, or evidence of renal failure on autopsy. New onset of AF was recorded by electrocardiograms. Delirium was assessed with the CAM-ICU score [11].

While delirium and AF were recorded within 4 days after surgery, MI, stroke and acute renal failure were analyzed until hospital discharge with a maximum of 14 days after surgery. Additionally several other clinical variables were recorded. For details see Table 1.

**Table 1:** Baseline Patient Characteristics

Variable	*N* = 1170
Age – yr	65.7 ± 10.3
Male sex — no./total no. (%)	865/1163 (74.4%)
Preexisting conditions — no./total no. (%)	
Ischemic heart disease	859/1160 (74.1%)
Aorta ascendens aneurysm	155/1161 (13.4%)
Previous myocardial infarction	325/1159 (28.0%)
Chronic heart failure	253/1157 (21.9%)
Chronic obstructive pulmonary disease	97/1163 (8.3%)
Current smoking	238/1162 (20.5%)
Peripheral vascular disease	82/1157 (7.1%)
Chronic kidney disease	131/1159 (11.3%)
Diabetes mellitus	280/1163 (24.1%)
Previous stroke	75/1160 (6.5%)
Chronic arterial hypertension	963/1159 (83.1%)
Drug history no./total no. (%)	
Beta blocker	721/1163 (62.0%)
ACE inhibitor	591/1163 (50.8%)
Logistic EuroSCORE	4.2 ± 2.5
Type of surgery performed — no./total no. (%)	
Coronary artery bypass graft (alone)	506/1163 (43.5%)
Aortic valve replacement/ reconstruction (alone)	247/1163 (21.2%)
Mitral valve replacement/ reconstruction (alone)	40/1163 (3.4%)
Aorta ascendens replacement (alone)	35/1163 (3.0%)
Combined procedures	318/1163 (27.3%)
Other type of surgery^a^	17/1163 (1.5%)
Time of procedures — minutes / total no.	
Duration of cardiopulmonary bypass	231.5 ± 59.8 / 1159
Duration of aortic cross clamping	77.6 ± 25.5 / 1156
Endpoints (adjusted)	
Death — no. (%)	9/1163 (0.8%)
Myocardial infarction — no. (%)	87/1026 (8.5%)
Stroke — no. (%)	17/1106 (1.5%)
Acute renal failure — no. (%)	52/1072 (4.9%)
New-onset atrial fibrillation — no. (%)	242/882 (27.4%)
Delirium — no. (%)	148/967 (15.3%)

^a^Plus–minus values are means ± SD. EuroSCORE denotes European System for Cardiac Operative Risk Evaluation. Note that patients could have had multiple events, e.g. patients suffering from both stroke and acute renal failure. Delirium was assessed by the Confusion Assessment Method for the Intensive Care Unit (CAM-ICU Score)

### Genotyping

Genomic DNA was isolated from whole blood of patients using standard procedures. Genotyping was performed for 1224 samples (500 cases and 724 controls) on the Illumina Human CoreExome-24 BeadChip (Illumina, Inc., San Diego, CA, USA) comprising 547,644 markers.

### Quality control of genotyped data

Markers were called using zCall [12], an algorithm specifically designed for calling low frequency variants. Samples with the following criteria were excluded: Call rate < 98%, heterozygosity > mean + 3x standard deviation (SD), duplicate samples and related samples (IBD > 0.185). Equally, markers with the following criteria were excluded: Call rate < 95%, failing Hardy-Weinberg-equilibrium tests in controls (*p* < 0.00001), markers mapping to chromosome 0, markers with differential missingness between batches (*p* < 10^− 5^) duplicate and triallelic markers as well as INDELs. For the principal components analysis (HAPMAP PCA), data were merged to the HAPMAP Phase III CEU, CHB and YRI populations. Variants with a minor allele frequency (MAF)-threshold smaller than 0.05 and with a *p*-value of the statistical test for Hardy Weinberg Equilibrium (HWE) in control samples p < 10^− 5^ were excluded. A batch QC was performed on the remaining samples. Data were again analyzed using PCA and flashpca [13]. Samples outside the rectangle [median + − 3* standard deviation] were excluded from the analysis. This left 1170 samples and 522,502 variants for analysis.

### Phasing and imputation

Data were then phased using SHAPEIT v2 [14], excluding all variants not matching to the 1000 Genomes Phase I variants according to their allelic information and using only chromosomes 1 to 22. Data were phased using 100 states, 7 burn, 8 prune and 20 main iterations as well as an effective cohort size of 11,418. Data were then imputed using IMPUTE2 v3.0 [15] in fragments of 5 Mb. Fragments comprising less than 200 variants were merged before imputation. Imputation was performed using the 1000 Genomes Phase I cohort as reference with 20,000 Ne, 250 buffer and 10 burnin and 30 main iterations as well as 80 k and 500 k_hap, 3 outdp. Genotyped data were kept and not overwritten by IMPUTE2. Following imputation markers with an imputation INFO-Score < 0.8 were excluded, leaving 9,007,469 variants on chromosomes 1 to 22 for analysis.

### Statistical analysis

Analysis was performed on 1163 samples. 7 of the above samples were excluded with missing phenotype data. Association tests were performed using PLINK v1.9 [16]. Statistical analysis of phenotypic data was performed using R (v3.0.2). Fisher’s exact test was used for the analysis of categorical and binary data, Student’s t-tests were used for the analysis of continuous data. Also, to take into account possible combined effects of the investigated clinical outcomes, we performed a variable selection algorithm using a logistic regression and a stepwise procedure (forwards and backwards) based on the Akaike Information Criterion (AIC) in R as well as a random forest approach using r2VIM [17] with 500 trees, mtry = 1/3 node size proportion of 10% and 5 runs. Random forest, when using regression mode for a binary outcome, will return probabilities of class membership. The overlap of these three methods was calculated. Variables that occurred more than 2 times were used.

r2vim is a feature selection method that uses relative importance measures to estimate the predictive power of, in this case, covariates in respect to the outcome (here disease) using a random forest approach and is described further in [17]. Using these relative importance measures, features that are correlated with the outcome are selected. r2vim is a package that is available in R. In a stepwise regression approach the stepAIC function of R can be used to build statistical models (starting from a null model that contains no variables in forward selection and from the full model containing all covariates in backwards selection) that contain any number of covariates. The final model then contains all variables that are most predictive for the outcome. In each step a covariate is added or deleted and the improvement of the model is tested against a previous model using the AIC. The AIC is a statistical measure of the goodness of fit of a model to a particular dataset (in this case disease outcome and clinical phenotypes).

Logistic regression analysis was performed on AF, delirium, MI, renal failure, stroke and the composite. The composite is a “compound” phenotype stating whether or not a sample had a phenotype or not. Genotypes were presented as dosage data between 0 and 2 and the *p*-value of association as well as the Odds ratios and their respective 95% confidence intervals were recorded. All analyses were adjusted for EuroSCORE (European System for Cardiac Operative Risk Evaluation) [18], age, diabetes mellitus, type of surgery, and baseline creatinine. Analysis of MI was adjusted for use of any cardiac assist device; stroke for severe sepsis/ septic shock; AF for cholesterol lowering drugs and smoking; delirium for any delirium medications, re-thoracotomy, cholesterol lowering drugs, center, NYHA (New York Heart Association) class, severe sepsis/ septic shock; renal failure was not adjusted for any additional covariate. Since one individual might have multiple of the outcomes, two types of analyses were performed: adjust each phenotype for each of the other outcomes to identify variants unique for each phenotype or without adjusting for other outcomes. Here, variants that might overlap between the outcomes are analyzed. A complete list of all adjustments is provided in the supplementary (Additional file 3: Table S1).

In this analysis we considered the widely excepted *p*-value threshold of 5 × 10^− 8^ as genome-wide significant and 1 × 10^− 5^ as nominally significant.

In total, 882 samples were analyzed for AF, 967 for delir, 1026 for MI, 1072 for renal failure and 1106 for stroke.

## Results

### Baseline characteristics

**Fig. 1:**
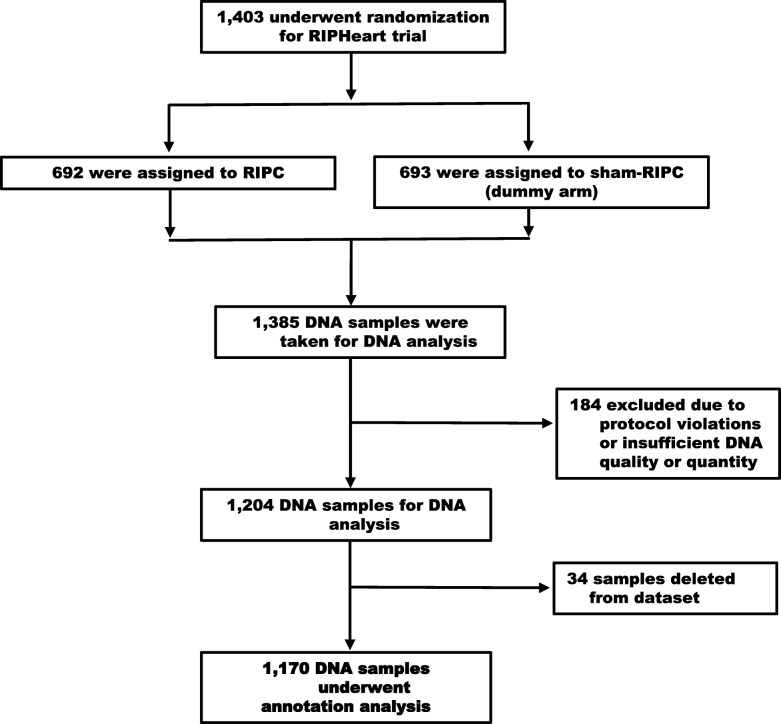
Randomization and Follow-up. Of the 1403 randomized patients, 1170 were included in the GWAS annotation analysis

A total of 1403 patients underwent randomization in the RIPHeart study, 1170 of these patients were included in the GWA analysis (Fig. 1). Baseline demographic and clinical characteristics are shown in Table 1. A wide range of cardiac surgical procedures were included. More than 27% of the original patients had congestive heart failure with NYHA class III or higher and 31% of patients had an EuroSCORE ≥6 or higher before surgery representing a high proportion of high-risk patients [6].

Postoperative AF was seen in 27.4% (242/882) of the patients. Delirium occurred in 15.3% (148/967), MI in 8.5% (87/1026), acute renal failure in 4.9% (52/1072) and stroke in 1.5% (17/1106).

### Explorative genotype analysis

A total 9,007,469 imputed markers with an imputation info score higher than 0.8 were available for analysis. We performed analyses with and without an adjustment for all other outcomes and a composite. The GWAS results are depicted using Manhattan plots (Additional file 4: Figure S1) and quantile-quantile (Q-Q) plots (Additional file 5: Figure S2) for each analysis and each complication. In this analysis we considered the widely excepted p-value threshold of 5 × 10^− 8^ as genome-wide significant and 1 × 10^− 5^ as nominally significant. Results presented were performed with adjustment for all other outcomes and the composite.

Only one SNP reached genome-wide significance of *p* < 5 × 10^− 8^(rs78064607, located in an intron of PH domain and leucine rich repeat protein phosphatase 2 gene (*PHLLP2*), *p* = 3.77 × 10^− 8^, (OR_L95 = 0.01, OR_U95 = 0.09, OR with 95% CI 0.02) and was associated with *renal failure*.

The complete list of SNPs showing associations with genes and exhibit the pre-defined p-value of *p* < 1 × 10^− 5^ from the GWAS with regard to AF, MI, delirium, and renal failure, and stroke is shown in the Additional file 6: Table S2. SNPs with lowest *p*-values for each complication are shown in Table 2.

**Table 2:** List of related SNPs. Summary of SNPs with lowest p-values, influencing known gene loci for each complication, adjusted for each complication

Chr	SNP	Base pair	Gene symbol	Gene	*P* value	OR with 95% CI	OR_L95	OR_U95	complication
2	rs7557600	48,772,202	STON1	Stonin1, may be involved in the endocytic machinery	7.25e-07	0.49	0.37	0.65	AFF
3	rs115155878	114,055,988	ZBTB20	Zinc finger and BTB domain containing 20, may be a transcription factor that may be involved in hematopoiesis, oncogenesis and immune response	8.45e-07	0.21	0.11	0.39	AFF
13	rs9563027	51,708,406	LINC00371	Long intergenic non-protein coding RNA 371	9.82e-07	0.56	0.45	0.71	AFF
5	rs4574581	89,925,895	GPR98	G protein-coupled receptor 98, receptor that may have an important role in the development of the central nervous system	2.43e-06	0.54	0.42	0.70	AFF
2	rs13008718	65,961,565	AC074391.1	lincRNA	9.99e-07	0.45	0.33	0.62	DELIR
14	rs188623516	46,926,170	LINC00871	Long intergenic non-protein coding RNA 871	4.02e-06	0.02	0.00	0.10	DELIR
3	rs727476	60,316,417	FHIT	Fragile histidine triad	1.70e-06	0.46	0.33	0.63	MI
7	rs9690969	40,541,259	SUGCT	Succinyl-CoA-glutarate-CoA-transferase	1.19e-06	0.44	0.31	0.61	MI
**16**	**rs78064607**	**71,723,181**	**PHLPP2**	**PH domain and leucine rich repeat protein phosphatase 2, plays a crucial role after I/R injury in the brain and oxidative stress injury in the kidney.**	**3.77e-08**	**0.02**	**0.01**	**0.09**	**RENFAIL**
8	rs189437718	134,655,419	SNORA40	Small nucleolar RNA, H/ACA Box 40	3.60e-07	0.05	0.01	0.15	RENFAIL
1	rs72654815	21,354,625	EIF4G3	Eukaryotic Translation Initiation Factor 4 Gamma 3	6.79e-07	0.11	0.05	0.27	RENFAIL
**11**	**rs77876049**	**122,936,811**	**HSPA8**	**Heat shock protein 8, HSPA8 seems to play an important role in the regulation of cellular processes after I/R injury both in the heart and in the brain.**	**9.14e-06**	**0.25**	**0.14**	**0.46**	**RENFAIL**
**7**	**rs79995619**	**33,511,328**	**BBS9**	**Bardet-Biedl Syndrome 9, mutations in this genes are associated with the Bardet-Biedl syndrome, which is characterized by renal failure**	**3.35e-07**	**0,001**	**0.00**	**0.06**	**STROKE**
3	rs181832941	189,567,428	TP63	Tumor protein p63	3.65e-07	0.01	0.00	0.06	STROKE
18	rs140914711	41,414,229	RNU6-443P	RNA, U6 small nuclear 443, pseudogene	4.07e-07	0.04	0.01	0.13	STROKE
**1**	**rs192540202**	**237,511,541**	**RYR2**	**Ryanodine receptor 2, calcium channel in the myocard muscle**	**6.33e-07**	**0.02**	**0.00**	**0.08**	**STROKE**

Bold: genes with supposed association to I/R injury

SNPs with lowest p-values located in regions associated with genes in patients with complications after cardiac surgery, adjusted for each outcome are: *STON1* (stonin1, rs7557600, *p* = 7.25 × 10^− 7^), *ZBTB20* (Zinc finger and BTB domain containing 20, rs115155878, *p* = 8.45 × 10^− 7^), *LINC00371* (Long intergenic non-protein coding RNA 371, rs9563027, *p* = 9.82 × 10^− 7^) and *GPR98* (G protein-coupled receptor 98, rs4574581, *p* = 2.43 × 10^− 6^) for *AF*, *AC074391.1* (rs13008718, *p* = 9.99 × 10^− 7^), *LINC00871* (Long intergenic non-protein coding RNA 871, rs1886223516, *p* = 4.02 × 10^− 6^) for *delirium*, *FHIT* (Fragile histidine triad, rs727476, *p* = 1.70 × 10^− 6^) and *SUGCT* (Succinyl-CoA-glutarate-CoA-transferase, rs9690969, *p* = 1.91 × 10^− 6^) for *MI*, *SNORA40* (Small nucleolar RNA, H/ACA Box 40, rs189437718, *p* = 3.60 × 10^− 7^), *EIF4G3* (Eukaryotic Translation Initiation Factor 4 Gamma 3, rs72654815, *p* = 6.79 × 10^− 7^) for *renal failure* and *BBS9* (Bardet-Biedl Syndrome 9, rs79995619, *p* = 3.35 × 10^− 7^), *TP63* (Tumor protein p63, rs181832941, *p* = 3.65 × 10^− 7^), *RNU6-443P* (RNA, U6 small nuclear 443, rs140914711, *p* = 4.07 × 10^− 7^) and (*RyR2* (Ryanodine receptor 2, rs192540202, *p* = 6.33 × 10^− 7^) for *stroke*.

Besides adjusted analysis, the composite, meaning a “compound” phenotype stating whether or not a sample had a phenotype or not, comprises five SNPs, three with association with genes: *RP5-968 J1.1* (rs200890 *p* = 1.19 × 10^− 6^), *DUSP4* (Dual Specificity Phosphatase 4, rs4732926, *p* = 5.53 × 10^− 6^, OR_L95 = 0.47, OR_U95 = 0.74) and *WLS* (Wntless Wnt Ligand Secretion Mediator) and *GNG12-AS1 (*GNG Antisense RNA 1), (rs74081211, *p* = 6.25 × 10^− 6^).

**Fig. 2:**
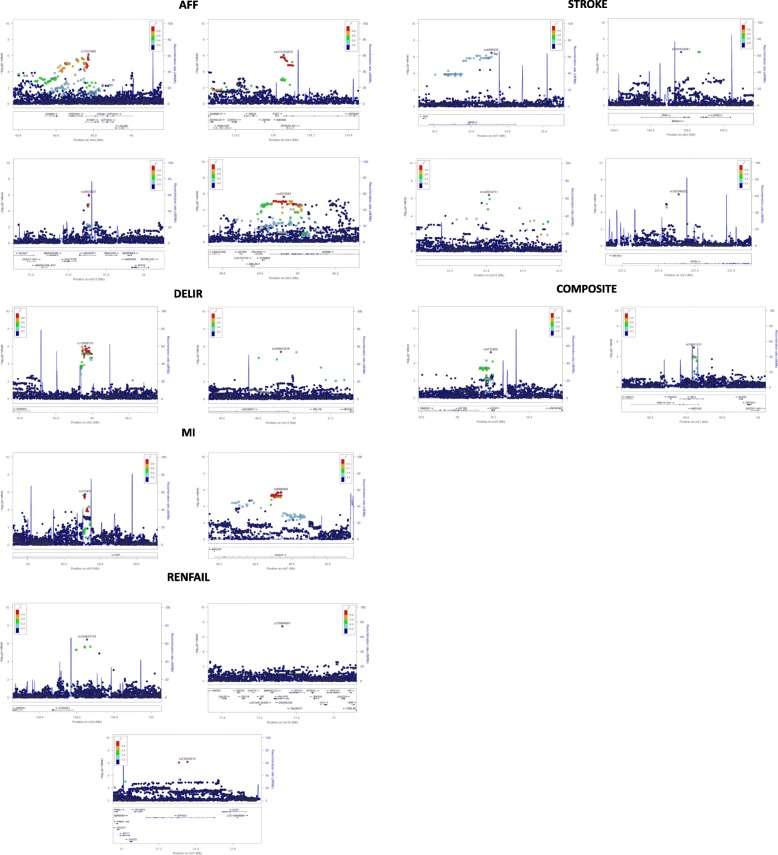
**a-f** SNP loci with lowest *p*-values associated with **a**) AF, **b**) delirium, **c**) MI, **d**) renal failure and **e**) stroke

Loci of SNPs with lowest p-values in genes are shown in Fig. 2.

## Discussion

As postoperative organ dysfunction is common after cardiac surgery, previous studies have performed preoperative genomic characterization of patients to identify genotypes, which render the patients vulnerable for the development of a specific organ dysfunction. Kertai et al. identified genetic variants in patients exhibiting AF and MI after cardiac surgery [3, 4]. Another recent study reported genetic variants concerning acute kidney injury after cardiac surgery [5]. To identify common genetic variants associated with the main complications AF, delirium, MI, acute renal failure and stroke, we performed a GWAS study with DNA samples of 1170 patients after cardiac surgery. We identified one SNP reaching genome-wide significance (p < 5 × 10^− 8^) and nearly 150 SNPs which reached the a priori defined discovery threshold of p < 1 × 10^− 5^. Since one individual might have multiple of the outcomes, we performed two types of analyses. Either we adjusted each phenotype of the other outcomes to identify variants unique for each phenotype or performed analyses without adjusting for each other. Here, variants might overlap between the outcomes and influenced genes could have effects to several organs. Besides, we identified a “compound” phenotype stating whether or not a sample has a phenotype or not.

*DUSP4* (rs4732926, Dual Specificity Phosphatase 4) was identified as a compound phenotype. *DUSP4* is an inducible nuclear phosphatase that is involved in regulating cardiovascular function under oxidative stress [19]. *DUSP4* −/− mice showed an increase of I/R-induced infarct caused by an over activation of p38, a stress-activated and pro-inflammatory kinase. In accordance to this, overexpression of *DUSP4* in endothelial cells prevents hypoxia/ reoxygenation-induced apoptosis via the upregulation of eNOS [20]. Therefore, it would be worthwhile to have a closer look inside the exact role of *DUSP4* during cardiac surgery.

rs78064607, the only SNP with genome-wide significance in our study is located in *PHLPP2* (PH Domain and Leucine Rich Repeat Protein Phosphatase 2) and was associated with increased risk of acute kidney injury. *PHLPP2* is a phosphatase, important for the regulation of Akt kinases and PKC isoforms [21, 22]. Akt belongs to the so called pro-survival kinases, involved in the protective pathway during myocardial ischemia/ reperfusion [23]. Akt controls the balance between cell survival and apoptosis, as well as proliferation and cellular quiescence. Activation of PI3K/Akt seems to be protective against I/R injury. *PHLPP2* dephosphorylates Akt (precisely Akt1 and Akt3) and therefore inactivates Akt. *PHLPP2* inhibition leads to neuronal protection after cerebral ischemia/reperfusion injury in rats [24, 25]. The imbalance of cell pro-death and pro-survival signaling pathways determines the neuronal fate during ischemia/reperfusion injury. In a rat model of I/R injury it was shown that inhibition of *PHLPP2* attenuates cell death in I/R injury. Very recently, it was demonstrated that *PHLPP2* plays a pivotal role in acetaminophen induced oxidative renal toxicity by influencing Nrf2 stability via Akt1/Gsk3b/Fyn kinase axis [26]. Down regulation of *PHLPP2* by morin, a bioflavonoid, significantly prevented the toxicity induced renal damages. In this respect, down regulation of *PHLPP2* may provide positive effects in the kidney and in the brain, two vital organs affected by cardiac surgery. In line with our GWAS of complications after cardiac surgery we found the SNP rs78064607 located in the *PHLPP2* gene with a genome-wide significance. Because the SNP is located in the intron region or intergenic region, respectively, of the gene, the exact effect on the *PHLPP2* gene cannot be evaluated. Probably an enhancer/ silencer region is affected, leading to up or down regulation of the *PHLPP2* gene. Association of possible changes in the expression of *PHLPP2* with increased occurrence of complications, especially RENFAIL after cardiac surgery could be a hind of an increased expression of *PHLPP2*. But this assumption is highly speculative and has to be confirmed by further gene expression analyses.

*ZBTB20* (Zinc finger and BTB domain containing 20, rs115155878 is related to this gene) is widely expressed in human hematopoietic cells, including DCs, monocytes, B cells and T cells. *ZBTB20* deficiency in mice attenuated TLR-triggered production of pro inflammatory cytokines in macrophages [27]. This could have an influence of mechanisms within the scope of I/R.

For stroke, rs4098926, the SNP with the lowest *p*-value for this complication is located in the *BBS9* gene. *BBS9* is also known as parathyroid hormone-responsive B1 gene (PTHB1). Mutations of *BBS9* are related with the Bardet-Biedl syndrome, a rare genetic disorder with highly variable symptoms. The underlying cause is malfunction of primary cilia, a key component of cellular communication function as signal transduction antennae. Kidney disease is a key feature and major cause of early mortality of patients with Bardet-Biedl syndrome. Intact cilia are critical under kidney injury conditions caused by ischemia/ reperfusion, because cilia are sensors of damages and activate cell proliferation probably to promote renal recovery [28, 29]. Changes in *BBS9* could contribute to higher complication rate concerning the kidney in patients undergoing cardiac surgery and further investigations of involvement of *BBS9* in postoperative renal injuries are worthwhile. Additionally, the exact role of *BBS9* in the pathogenesis of stroke after cardiac surgery has to be evaluated.

rs192540202 is located in an intron region of *RyR2* (ryanodine receptor 2). *RyR2* is primarily found in cardiac muscle and forms a Ca^2+^ release channel on the membrane of the sarcoplasmic reticulum. Abnormal *RyR2* function is recognized as an important part of the pathophysiology of heart failure, especially contractile dysfunction, arrhythmia and sudden death [30, 31]. Numerous studies revealed that abnormal Ca^2+^ homeostasis may play an important role in the electric and contractile remodeling accompanying sustained atrial fibrillation [32, 33]. Very recently, Xie et al. identified a link between oxidative stress and *RyR2* [34]. Mice with mutations in the *RyR2* receptor exhibited mitochondrial dysfunction, increased reactive oxygen species production and increased AF susceptibility. Because oxidative stress and mitochondria dysfunction plays a pivotal role in the pathogenesis of I/R injury of organs, changes in *RyR2* receptor associated with disturbed Ca^2+^ homeostasis could contribute to higher risk of complication after cardiac surgery.

*RyR2* also has an impact on Ca^2+^ homeostasis in the brain during cerebral ischemia [35]. In a rat model of brain ischemia, Bull et al. could demonstrate that amplification of Ca^2+^ by *RyR2* entry signals may contribute to cortical neuronal death.

Very interestingly, knockdown of *RyR2* in a spinal cord injury model in rats inhibited the increase of pro-inflammatory cytokines, improved mitochondrial dysfunction and reduced oxidative stress [36]. Because release of pro-inflammatory cytokines, massive ROS production and mitochondrial dysfunction are the main causes of I/R injury of organs, examination of the exact role of *RyR2* could be very interesting.

rs77876049 is located in the *HSPA8* gene. Although this SNP has a higher p-value, it is reasonable to have a closer look at this protein because of its interesting involvement in the mechanisms of I/R injury. *HSPA8* (Heat Shock Protein 8, also known as Hsc70 or Hsp73) is a member of the heat shock protein 70 family and facilitates the correct folding of newly translated or misfolded proteins. *HSPA8* plays an important role in signal transduction, apoptosis, protein homeostasis, cell growth and differentiation. Zou et al. could demonstrate that *HSPA8*, constitutively expressed in the myocardium, is released during ischemia/ reperfusion and induces the myocardial inflammatory response and modulates cardiac function [37]. Acute myocardial ischemia can lead to a cascade of cellular and ischemic tissue, causing irreversible damage. In myocardial ischemia and reperfusion, the myocardial cells release *HSPA8* and reduce myocardial cell injury [38]. Thus, *HSPA8* plays a critical role in regulating the myocardial innate immune system and cardiac function after ischemia/ reperfusion. Probably, *HSPA8* specifically has a protective effect in patients undergoing open heart surgery [39]. It has also been shown that Chaperone-mediated autophagy (CMA), under involvement of *HSPA8*, of damaged or leaky *RyR2* receptors after I/R may play a protective role after I/R injury and could contribute to myocardial remodeling [40]. A combined functional impairment of *HSPA8* and *RyR2* in patients undergoing cardiac surgery could contribute to increased myocardial complications because of lack of functioned *RyR2* receptors and the inability to remove damaged *RyR2* receptors by CMA. *HSPA8* also plays a protective role in the process of ischemic stroke by protection of nerve cells and inhibition of neuronal apoptosis [41–43]. *HSPA8* seems to play an important role in the regulation of cellular processes after I/R injury both in the heart and in the brain. Therefore, patients with variants in this gene might have an increased complication rate after cardiac surgery. So, *HSPA8* might be a prognostic factor, but validation of these findings will require additional studies with independent subject panels.

None of the further identified genetic variants associated with atrial fibrillation [44, 45], myocardial infarction [46, 47], stroke [48] or renal dysfunction [49] in non-surgical patients was found in our analyses. In contrast, some of the described genetic variants associated with complications after cardiac surgery, namely *BBS9* in renal dysfunction [5], were found in our study. This indicates a unique pathogenesis in the subset of ischemia/ reperfusion after cardiac surgery that differs from pathogenesis in non-surgical patients. Surprisingly, neither Kertai et al. [3] nor our study could replicate previously reported associations between common genetic variants at the 9p21 locus and risk for myocardial infarction after cardiac surgery [50, 51]. Probably, variations in study design or differences in data analysis could be reasons for these variations and further studies are needed to explain these discrepancies.

## Conclusions

Here we report the first GWAS in a cohort of patients at risk of AF, delirium, MI, acute renal failure and stroke after cardiac surgery. We identified several polymorphisms associated with these complications. In most cases, loci are noncoding, and many loci are far from discovered genes in non-coding regions, the effects of SNPs on genes are completely unknown or the functions of the influenced genes are unknown. Furthermore, GWAS almost exclusively detects the effects of common SNPs, any rare variants will not be detected. Nevertheless, we identified some very interesting potential correlations between genetic polymorphisms and the occurrence of complications. In particular, the described concurrence of *HSPA8* and *RyR2* for atrial fibrillation and myocardial infarction, the involvement of *DUSP4* in I/R injury, the role of *PHLPP2* in developing complications after cardiac surgery and the involvement of *BBS9* in renal dysfunction could be interesting for further future examinations. Follow-up studies are needed to transfer these findings into biological insights that could result in predictive and therapeutic advances in the perioperative care of cardiac surgery patients.

## Additional files

Additional file 1: **Figure S3.** English synopsis of study protocol. (PDF 105 kb)

Additional file 2: **Figure S4.** Study protocol of the RIPHeart study. (PDF 4311 kb)

Additional file 3: **Table S1.** List of adjustment variables. (PDF 65 kb)

Additional file 4: **Figure S1.** Manhattan Plot of genome-wide association with AF, delirium, MI, renal failure and stroke. The x-axis represents the chromosomes in physical order, the y axis showing –log_10_(p) for all single nucleotide polymorphisms (SNPs). a) Adjustment for all other outcomes: one SNP reached genome-wide significance (*p* < 5 × 10^− 8^, red line) and 139 SNPs reached the predefined threshold of *p* < 1 × 10^− 5^ (blue line). b) No adjustment for all other outcomes and composite: 132 SNPs reached the predefined threshold of p < 1 × 10^− 5^. (PDF 498 kb)

Additional file 5: **Figure S2.** Quantile-quantile plot showing the expected distribution of association test statistics across the SNPs compared to the observed values for AF, delirium, MI, renal failure and stroke. a) Adjustment for all other outcomes. b) No adjustment for all other outcomes and composite. (PDF 305 kb)

Additional file 6: **Table S2.** Complete table of SNPs reaching the predefined threshold of *p* < 1 × 10^− 5^ with and without adjustment for all other complications. (PDF 1177 kb)

## Abbreviations

AF: Atrial Fibrillation
GWAS: Genome Wide Association Analysis
ICU: Intensive Care Unit
MI: Myocardial Infarction
SNP: Single Nucleotide Polymorphism
