# Supplementary material for: Genome-wide association study of myocardial infarction, atrial fibrillation, acute stroke, acute kidney injury and delirium after cardiac surgery – a sub-analysis of the RIPHeart-Study
Source: BMC Cardiovasc Disord. 2019 Jan 24;19:26. doi: 10.1186/s12872-019-1002-x (PMC6345037; doi:10.1186/s12872-019-1002-x)
Supplement: Supplementary file 1 — Figure S3. English synopsis of study protocol. (PDF 105 kb) [file 12872_2019_1002_MOESM1_ESM.pdf]

Synopsis

Title of the study	Remote Ischaemic Preconditioning for Heart Surgery
Short Titel	RIPHeart - Study
Indication	Patients undergoing isolated on-pump coronary-artery bypass graft (CABG) surgery
Study design	The study is a prospective, multicentre, randomised, controlled clinical study.
Primary endpoints	The primary endpoint is defined as a composite of<br>• all-cause mortality<br>• non-fatal myocardial infarction<br>• any new stroke and/or<br>• acute renal failure<br>until hospital discharge (with a maximum of 14 days after surgery)
Secondary endpoints	1. The occurrence of any individual component of the composite endpoint at 30 days and 3 and 12 months after surgery (phone interview)<br>2. The cumulative duration of invasive ventilator support (up to d30 post operation)<br>3. The cumulative duration of non-invasive ventilation (up to d 30 post operation)<br>4. Length of stay on the intensive care (ICU)<br>5. Need of catecholamines<br>6. Total hospital stay<br>7. Troponin T (preoperative, 6, 12, 24, and 48 h after surgery)<br>8. Troponin I (preoperative, 6,12, 24, and 48 h after surgery)<br>9. Renal function<br>10. Vasopressor and inotropic support (yes/ no)<br>11. New onset of atrial fibrillation<br>12. Incidence of postoperative delirium within 4 days after surgery (yes/no)<br>13. Use of any delirium medication within 4 days after surgery (yes/no)<br>14. Use of any cardiac assist devices intraoperative and postoperative within 30 days (yes/no)
Sample size	N= 2070 patients, N=1035 per group
Study population	Key inclusion criteria:<br>▪ patients ≥ 18 years<br>▪ on-pump isolated coronary-artery bypass graft (CABG) surgery<br>▪ Informed consent<br><br>Key exclusion criteria:<br>▪ Off-pump precedure<br>▪ concomitant diagnosis (e.g., ejection fraction <30%, diabetes mellitus, Instable angina pectoris, increased baseline troponin concentration; dialysis)<br>▪ nicorandil and/or sulfonylurea and/or $P2Y_{12}$ platelet receptor inhibitors<br>▪ pregnancy
Interventions	Experimental intervention: RIPC will be induced prior to skin incision by 4 cycles of upper limb ischaemia (5 min blood pressure cuff inflations to a pressure of at least 220 mmHg and 5 min cuff deflations).

	Control interventions:<br>Sham-RIPC will be induced by 4 cycles of 'pseudo'-ischaemia (5 min blood-pressure cuff inflations to a pressure of at least 220 mmHg and 5 min cuff deflations) using a dummy arm, under Sevoflurane based anaesthesia<br>Duration of intervention per patient: 40 minutes<br>Follow-up per patient: Discharge, 30 days, month 3, 6 and 12 after surgery
Statistical Analysis	Efficacy: The primary endpoints AUC of cTnT and AKI will be compared in the following way: Firstly, the experimental arm (RIPC group) will be compared with the Sham-RIPC arm. Secondly, if results are significant for at least one of the two co-primary endpoints, the experimental arm will be compared with the RIPC/Propofol arm for the two primary endpoints. Group comparisons will be tested using appropriate 2 sample tests (t-test in case of normal distribution or Wilcoxon-Mann-Whitney test otherwise for AUC of cTnT) and the χ^2-test for AKI.<br>Description of the primary efficacy analysis and population: Tests will be two-sided using Bonferroni-corrected significance levels of alpha = 2.5 % to account for the two primary endpoints. Power estimation bases on a power of 95 % for the comparisons of RIPC vs. Sham-RIP. All patients with available co-primary endpoints will be included in the analysis population.<br>Safety: Safety data on RIPC will be evaluated descriptively and summarised by treatment arm, including all recruited study patients (safety population).<br>Secondary endpoints will be compared between the treatment arms with t-tests, Wilcoxon-Mann-Whitney tests, χ^2-tests and log-rank test. Furthermore, appropriate regression models will be used for adjusted analysis.
