# Supplementary material for: Genome-wide association study of myocardial infarction, atrial fibrillation, acute stroke, acute kidney injury and delirium after cardiac surgery – a sub-analysis of the RIPHeart-Study
Source: BMC Cardiovasc Disord. 2019 Jan 24;19:26. doi: 10.1186/s12872-019-1002-x (PMC6345037; doi:10.1186/s12872-019-1002-x)
Supplement: Supplementary file 2 — Figure S4. Study protocol of the RIPHeart study. (PDF 4311 kb) [file 12872_2019_1002_MOESM2_ESM.pdf]

Protocol

This trial protocol has been provided by the authors to give readers additional information about their work.

Protocol for: Meybohm P, Bein B, Brosteanu O, et al. A multicenter trial of remote ischemic preconditioning for heart surgery. N Engl J Med 2015;373:1397-407. DOI: 10.1056/NEJMoa1413579

Supplementary Appendix Study Protocol

Remote Ischemic Preconditioning for Heart Surgery (RIPHeart-Study)

Corresponding author: Prof. Dr. Patrick Meybohm, MD, MHBA**;** Department of Anaesthesiology, Intensive Care Medicine and Pain Therapy, University Hospital Frankfurt**,** Theodor-Stern-Kai 7**,** 60590 Frankfurt am Main Germany, Email: patrick.meybohm@kgu.de

This supplement contains the following items:

1) **Study protocol**
   a. Study protocol Version 1.0 – 17/10/2010 (German version)
   b. Methods and Design manuscript (Meybohm P, et al. *Eur Heart Journal* 2012; 33:1423–1425)
   c. Study protocol Amendment Version 1 – 07/04/2014 (German version)
   d. Summary of changes
2) **Statistical Analysis Plan**
   a. Statistical Analysis Plan V1.0 – 08/12/2014
   b. Statistical Analysis Plan V2.0 – 08/01/2015
   c. Summary of changes

Prüfplan

Prospektive, randomisierte, doppelblinde, multizentrische klinische Prüfung zum Einfluss der ischämischen Fern- (*Remote*) Präkonditionierung bei herzchirurgischen Patienten

Kurztitel:

RIPHeart-Studie

Gefördert von der Deutschen Forschungsgemeinschaft (DFG)

Förderkennzeichen: ME 3559/1-1

Studienleitung	Biometrie
**PD Dr. med. Patrick Meybohm**<br>**Prof. Dr. med. Berthold Bein**<br>Klinik für Anästhesiologie und Operative Intensivmedizin<br>**Prof. Dr. med. Jochen Cremer**<br>Klinik für Herz- und Gefäßchirurgie<br>Universitätsklinikum Schleswig-Holstein, Campus Kiel<br>Schwanenweg 21<br>24105 Kiel<br><br>**Prof. Dr. Dr. med. Kai Zacharowski**<br>Klinik für Anästhesiologie, Intensivmedizin und Schmerztherapie<br>Klinikum der J.-W. von Goethe-Universität<br>Theodor-Stern-Kai 7<br>60590 Frankfurt am Main	**Dr. Dirk Hasenclever**<br>Institut für Medizinische Informatik, Statistik und Epidemiologie, Universität Leipzig<br>Härtelstraße 16-18, 04107 Leipzig<br><br>Koordination<br><br>**Holger Bogatsch**<br>Zentrum für Klinische Studien Leipzig - KKS<br>Universität Leipzig<br>Härtelstraße 16-18, 04107 Leipzig

Datum der Fassung: 17.10.2010

Status der Fassung: final 1.0

© 2010

Schriftführer:
Meybohm P, Bein B, Cremer J, Zacharowski K, Hasenclever D, Brosteanu O, Bogatsch H.

Erstellt auf der Grundlage der Prüfplanvorlage des Zentrums für Klinische Studien Leipzig - KKS

INHALTSVERZEICHNIS

ALLGEMEINE INFORMATIONEN 5

Verantwortliche Personen 5
Prüfplan-Synopse 8
Flow Chart 11

1 RATIONALE UND FRAGESTELLUNG 12

1.1 Hintergrund 12
1.2 Darstellung der Studientherapie 13
1.3 Nutzen-Risiko Abschätzung 15

2 STUDIENZIELE 16

2.1 Primäres Ziel/ primärer Endpunkt 16
2.2 Sekundäre Ziele/ sekundäre Endpunkte 18

3 STUDIENBESCHREIBUNG 19

3.1 Studiendesign 19
3.2 Personelle und technische Anforderungen an Prüfzentren 20
3.3 Teilnehmende Zentren und Zahl der Patienten 20
3.4 Erwartete Studiendauer 20
3.5 Studienabbruch 21

4 STUDIENPOPULATION 22

4.1 Einschlusskriterien 22
4.2 Ausschlusskriterien 22
4.3 Begründung für den Einschluss spezieller Populationen 24

5 INDIVIDUELLER STUDIENABLAUF 24

5.1 Prüfung der Ein- und Ausschlusskriterien 24
5.2 Patientenaufklärung und -einwilligung 25
5.3 Aufnahme in die Studie 26
5.4 Beschreibung des Studienablaufes 28
5.5 Nachbeobachtung 34
5.6 Vorzeitiger Abbruch der Nachbeobachtung 35

6 UNERWÜNSCHTE EREIGNISSE UND VIGILANZSYSTEM 35

6.1 Unerwünschte Ereignisse (AE/SAE) 35
6.2 Sicherheitsanalysen 38
6.3 Therapeutische Maßnahmen im Rahmen eines unerwünschten Ereignisses 38

7 BIOMETRISCHE ASPEKTE DER STUDIE 39

7.1 Randomisationsalgorithmus 39
7.2 Endpunkte der Studie 39
7.3 Statistische Formulierung der Studienfrage 41
7.4 Fallzahldiskussion 41
7.5 Verfahren zur Datenanalyse 43
7.6 Zwischenauswertungen 44
7.7 Endauswertung 45

8 WISSENSCHAFTLICHE BEGLEITPROJEKTE 45

8.1 Genomanalysen 45

9 ETHISCHE GRUNDLAGEN 46
9.1 GCP-Erklärung 46
10 ANTRAGSTELLUNG 46
11 DOKUMENTATION 46
11.1 Patientendokumentationsbögen (CRF) 46
11.2 Datenmanagement und Archivierung 48
12 REFERENZBEGUTACHTUNGEN 49
12.1 Endpunkt Komitee 49
13 ÜBERWACHUNG DER STUDIE 50
13.1 Zugang zu Quelldaten 50
13.2 Monitoring 50
13.3 Audits 51
13.4 Unabhängige Überwachung der Studie 51
14 DATENSCHUTZ UND SCHWEIGEPFLICHT 51
15 ADMINISTRATIVE REGELUNGEN 52
15.1 Studiendurchführung nach Prüfplan 52
15.2 Nachträgliche Prüfplanänderungen 53
15.3 Finanzierung und Versicherungen 53
15.4 Publikationsvereinbarungen und Registrierung 54
16 LITERATURVERZEICHNIS 55
17 UNTERSCHRIFTEN ZUM PRÜFPLAN 62
18 ANERKENNUNG DES PRÜFPLANS 63
19 ANHANG 64
19.1 Confusion Assessment Method for the Intensive Care Unit (CAM-ICU) 64
19.2 EKG-Veränderungen 65
19.3 Abkürzungen 66
19.4 Teilnehmende Prüfzentren 67
19.5 European System for Cardiac Operative Risk Evaluation (EuroSCORE) 69
19.6 Transösophageale Echokardiographie 70
19.7 Klassifikation unerwünschter Ereignisse (AE/ SAE) 71

ALLGEMEINE INFORMATIONEN

Verantwortliche Personen

Studienleitung	**PD Dr. med. Patrick Meybohm (KAI)**<br>**Prof. Dr. med. Berthold Bein**<br>Klinik für Anästhesiologie und Operative Intensivmedizin<br>Universitätsklinikum Schleswig-Holstein, Campus Kiel<br>Schwanenweg 21, 24105 Kiel<br>Tel.: 0431-597 2971; Fax: 0431-597 3002<br>E-Mail: meybohm@anaesthesie.uni-kiel.de<br><br>**Prof. Dr. med. Jochen Cremer**<br>Klinik für Herz- und Gefäßchirurgie<br>Universitätsklinikum Schleswig-Holstein, Campus Kiel<br>Arnold-Heller-Straße 3 (Haus 18), 24105 Kiel<br>Tel.: 0431-597 4401; Fax: 0431-597 4402<br>E-Mail: jochen.cremer@uk-sh.de
Koordination der klinischen Prüfung	**PD Dr. med. Patrick Meybohm (KAI)**<br>**Prof. Dr. med. Berthold Bein**<br>Klinik für Anästhesiologie und Operative Intensivmedizin<br>Universitätsklinikum Schleswig-Holstein, Campus Kiel<br>Schwanenweg 21, 24105 Kiel<br>Tel.: 0431-597 2971; Fax: 0431-597 3002<br>E-Mail: meybohm@anaesthesie.uni-kiel.de<br><br>**Prof. Dr. Dr. med. Kai Zacharowski**<br>Klinik für Anästhesiologie, Intensivmedizin und Schmerztherapie<br>Klinikum der J.-W. von Goethe-Universität<br>Theodor-Stern-Kai 7, 60590 Frankfurt am Main<br>Tel.: 069-63015998; Fax: 069-63015881<br>E-Mail: direktion.anaesthesie@kgu.de
Projektmanagement	**Holger Bogatsch**<br>Zentrum für Klinische Studien Leipzig - KKS<br>Universität Leipzig<br>Härtelstr. 16-18, 04107 Leipzig<br>Tel.: 0341- 97 16154; Fax: 0341- 97 16259<br>E-Mail: holger.bogatsch@zks.uni-leipzig.de
Medizinischer Konsiliardienst	**PD Dr. med. Patrick Meybohm (KAI)**<br>**Prof. Dr. med. Berthold Bein**<br>Klinik für Anästhesiologie und Operative Intensivmedizin<br>Universitätsklinikum Schleswig-Holstein, Campus Kiel<br>Schwanenweg 21, 24105 Kiel<br>Tel.: 0431-597 2971; Fax: 0431-597 3002<br>E-Mail: meybohm@anaesthesie.uni-kiel.de
Datenmanagement	**Vera Schleicher, Bianca Scholze**<br>Zentrum für Klinische Studien Leipzig - KKS<br>Universität Leipzig

	Härtelstr. 16-18, 04107 Leipzig<br>Tel.: 0341- 97 16261; Fax: 0341- 97 16 259<br>E-Mail: vera.schleicher@zks.uni-leipzig.de<br>bianca.scholze@zks.uni-leipzig.de
Biometrie	**Dr. rer. medic. Dirk Hasenclever**<br>Institut für Medizinische Informatik, Statistik und Epidemiologie (IMISE)<br>Universität Leipzig<br>Härtelstr. 16-18, 04107 Leipzig<br>Tel.: 0341- 97 16121; Fax: 0341- 97 16109<br>E-Mail: dirk.hasenclever@imise.uni-leipzig.de
Monitoring	**Dr. rer. nat. Silke Hauer**<br>Zentrum für Klinische Studien Leipzig - KKS<br>Universität Leipzig<br>Härtelstr. 16-18, 04107 Leipzig<br>Tel.: 0341- 97 16252 ; Fax: 0341- 97 16219<br>E-Mail: silke.hauer@zks.uni-leipzig.de
Sicherheit	**Madlen Dörschmann**<br>Zentrum für Klinische Studien Leipzig - KKS<br>Universität Leipzig<br>Härtelstr. 16-18, 04107 Leipzig<br>Telefon: 0341- 97 16129; Fax: 0341- 97 16 278<br>Email: pharmacovigilance@zks.uni-leipzig.de
Referenzgremien	**PD Dr. rer. physiol. Martin Albrecht**<br>Universitätsklinikum Schleswig-Holstein, Campus Kiel<br>Klinik für Anästhesiologie und Operative Intensivmedizin<br>Labor Anästhesiologie, Haus 17, Raum 37<br>Michaelisstraße 5, 24105 Kiel<br>Tel.: 0431-597 1652; Fax: 0431-597 3002<br>E-Mail: albrecht@anaesthesie.uni-kiel.de
Förderer	**Deutsche Forschungsgemeinschaft (DFG)**<br>Dr. Simone Müller<br>Kennedyallee 40, 53175 Bonn<br>Telefon: 0228-885 2359; Telefax: 0228-885 2777<br>Email: simone.mueller@dfg.de<br><br>Förderkennzeichen: ME 3559/1-1
Independent Data Monitoring and Safety Committee (IDMC)	**Dipl. Inform. Jürgen Hedderich**<br>Institut für Medizinische Informatik und Statistik<br>Brunswiker Str. 10, 24105 Kiel<br>Telefon: 0431-597 3182; Fax: 0431-597 3193<br>Email: hedderich@medinfo.uni-kiel.de<br><br>**Prof. Dr. Georg Lutter**<br>Klinik für Herz- und Gefäßchirurgie<br>Universitätsklinikum Schleswig-Holstein, Campus Kiel<br>Arnold-Heller-Straße 3 (Haus 18), 24105 Kiel<br>Tel.: 0431-597 8036; Fax: 0431-597 4402

	Email: georg.lutter@uk-sh.de<br><br>**Prof. Dr. Georg von Knobelsdorff**<br>St. Bernward Krankenhaus<br>Treibestr. 9, 31134 Hildesheim<br>Telefon: 05121-901425<br>Email: anaesthesie@bernward-khs.de
Endpunkt Komitee	**Prof. Dr. med. Heribert Schunkert**<br>Medizinische Klinik II - Kardiologie<br>Universitätsklinikum Schleswig-Holstein, Campus Lübeck<br>Ratzeburger Allee 160, 23538 Lübeck<br>Tel.: 0451-500 2501; Fax: 0451-500 6437<br>Email: heribert.schunkert@uk-sh.de<br><br>**Prof. Dr. med. Norbert Frey**<br>Medizinische Klinik III - Kardiologie<br>Universitätsklinikum Schleswig-Holstein, Campus Kiel<br>Arnold-Heller-Straße 3 (Haus 6), 24105 Kiel<br>Tel.: 0431-597 1440; Fax: 0431 597-1470<br>Email: norbert.frey@uk-sh.de
Wissenschaftliche Begleitprojekte	**DNA-Probenbank**<br><br>**Prof. Dr. med. Stefan Schreiber**<br>Universitätsklinikum Schleswig-Holstein, Campus Kiel<br>Biobank popgen – Studienzentrale<br>Arnold-Heller-Strasse 3, Haus 3, 24105 Kiel<br>Tel.: 0431-597 3710; Fax: 0431-597 3730<br>E-Mail: info@popgen.de

Prüfplan-Synopse

**Titel der Studie**	Prospektive, randomisierte, doppelblinde, multizentrische klinische Prüfung zum Einfluss der ischämischen Fern- (Remote) Präkonditionierung bei herzchirurgischen Patienten
**Kurzbezeichnung**	RIPHeart-Studie
**Indikation**	Patienten mit geplantem elektiven herzchirurgischen Eingriff an der Herz-Lungen-Maschine
**Primäres Ziel**<br>**und Endpunkt**	Prüfung des Einflusses der Intervention auf ein Composite outcome (Gesamtmortalität, Myokardinfarkt, Schlaganfall, Akutes Nierenversagen) bis zur Krankenhausentlassung (bzw. maximal 14 Tage postoperativ)
**Sekundäre Ziele**<br>**und Endpunkte**	Evaluation der Intervention auf:<br>- Dauer der mechanischen Beatmung<br>- Verweildauer auf ITS bzw. im Krankenhaus<br>- Myokardschaden<br>- Nierenfunktion<br>- Postoperatives Delir<br>- Katecholaminbedarf<br>- Neu aufgetretenes Vorhofflimmern<br>- Einsatz mechanischer kardialer Unterstützungssysteme<br>- Einzelkomponenten des kombinierten Endpunkts bis zur Krankenhaus-entlassung (max. 14 Tage bei längerem Krankenhausaufenthalt) sowie 30 Tage, 90 Tage und 12 Monate postoperativ<br>- Myokardiale Dysfunktion (optional)<br>- Postoperative neurokognitive Dysfunktion (optional)
**Studiendesign**	Prospektiv, kontrolliert, randomisiert, multizentrisch, doppelblinde Studie
**Studienpopulation**	*Einschlusskriterien*<br>1. Elektiver herzchirurgischer Eingriff an der Herz-Lungen-Maschine (HLM), insbesondere Aortokoronare Bypassoperation, Aortenklappenersatz, Mitralklappenersatz oder -rekonstruktion, Aorta ascendens Ersatz oder Kombinationseingriff<br>2. Alter ≥18 Jahre<br>3. schriftliche Einwilligungserklärung des Patienten<br><br>*Ausschlusskriterien*<br>In Bezug auf den Eingriff:<br>1. Off-pump Verfahren; minimal-invasive Eingriffe über laterale Thorakotomie; Aorta descendens Ersatz; gleichzeitige Carotis-TEA, selektive antegrade Hirnperfusion während HLM-Stillstand<br>2. Voroperation an Herz/ Aorta<br>In Bezug auf die kardiale Anamnese:<br>3. Schwere linksventrikuläre Funktionsstörung (EF≤30%)<br>4. Chronisches/ intermittierendes Vorhofflimmern innerhalb der letzten 6 Monate präoperativ; Medikamentöse Einnahme von Digitalis-Präparaten, Amiodaron und/oder Antiarrhythmika Klasse I (z.B. Ajmalin, Flecainid, Propafenon, Tocainid)<br>5. Implantierter Herzschrittmacher oder Defibrillator<br>6. Myokardinfarkt innerhalb der letzten 7 Tage präoperativ, instabile Angina pectoris innerhalb der letzten 24 h präoperativ mit bereits präoperativen

	positiven Troponin T Anstieg<br>In Bezug auf weitere Begleiterkrankungen:<br>7. Schwere Niereninsuffizienz (Kreatinwert i.S. > 2,0 mg/dl (> 176 µmol/l)<br>8. Schwere Leberinsuffizienz (spontan INR > 1,8 und/oder Bilirubin i. S. > 8,8 mg/dl (> 150 µmol/l) und/oder nach ärztlicher Einschätzung)<br>9. Schwere Lungenerkrankung (SpO_2<85% bei Raumluft und/oder FeV1≤ 50% und/oder nach ärztlicher Einschätzung)<br>10. Schwerer Alkoholabusus<br>11. Schlaganfall in den letzten 2 Monaten präoperativ<br>12. NIH Stroke Scale > 2 präoperativ<br>13. Schwere neurologische Erkrankungen (z.B. Morbus Parkinson, Multiple Sklerose, Morbus Alzheimer, präoperatives Delir und/oder nach ärztlicher Einschätzung)<br>14. Akute Infektionen mit Antibiotikatherapie innerhalb der letzten 7 Tage präoperativ<br>15. Medikamentöse Einnahme von Sulfonylharnstoffen (Glibenclamid) und Nicorandil, die innerhalb von 24 h präoperativ nicht pausiert wurden, da für beide Medikamente Interaktionen mit potentiellen Konditionierungs-mechanismen gezeigt wurden<br>16. Peripher arterielle Verschlusskrankheit Stadium IV<br>17. Heparin-induzierte Thrombozytopenie Typ II<br>Weitere:<br>18. Teilnahme an anderen klinischen Prüfungen innerhalb der letzten 30 Tage<br>19. Infauste Prognose aufgrund von Nebenerkrankungen<br>20. weitergehende Beziehung zum Prüfer (z.B. Mitarbeiter, Verwandte, Kollegen)<br>21. Frauen während der Schwangerschaft und Stillzeit<br>22. Sprach-/ Verständigungsprobleme im Rahmen der Aufklärung<br>23. Mangelnde Kooperationsbereitschaft (Compliance)
**Patientenzahl**	n=2070 insgesamt, d.h. n=1035 pro Arm
**Therapie**	Bei allen Studienpatienten wird nach der Narkoseeinleitung an einem Oberarm eine manuelle Blutdruckmanschette angelegt. Zusätzlich wird ein Dummy-Arm mit einer zweiten manuellen Blutdruckmanschette neben den Interventionsarm gelagert, die dann zusammen abgedeckt werden. Durch eine unverblindete Studienassistentin/ Pflegekraft erfolgt anschließend die Bedienung einer der beiden Blutdruckmanschetten:<br>**Gruppe 1 - Ischämische Fern *(Remote)*- Präkonditionierung (RIPC):** Nach der Narkoseeinleitung wird mit der Blutdruckmanschette an dem Oberarm des Patienten für 5 Minuten ein Druck appliziert, der mindestens 30 mm Hg höher als der invasiv gemessene, systolische arterielle Blutdruck des Patienten ist und nicht < 200 mm Hg liegt. Nach dieser Ischämiephase wird 5 Minuten pausiert. Dieses Procedere wird viermal wiederholt.<br>**Gruppe 2 - Kontrolle:** Bei Patienten in der Kontrollgruppe werden nach Narkoseeinleitung mit der zweiten Blutdruckmanschette an dem Dummy-Arm entsprechend dem RIPC-Protokoll vier identischen Zyklen appliziert – Wechsel von jeweils 5 Minuten Aufpumpen bis zu einem Druck von 200 mm Hg und 5 Minuten Pause.
**Biometrie**	Der primäre zusammengesetzte Endpunkt ist binär. Die Bewertung des Therapieeffekts erfolgt über eine logistische Regression. Kovariaten neben der Therapie sind der EuroSCORE, der Diabetes-mellitus Status (Ja/Nein), und die Begleittherapie mit Statinen (Ja/Nein). Zentrumsunterschiede werden als Randomeffekt berücksichtigt. Die Testentscheidung erfolgt über den Wald-Test für den Koeffizienten des Therapieeffekts. Die Risikoreduktion wird als Odds-Ratio mit 95% Konfidenzintervall quantifiziert.<br>Die primäre Auswertung erfolgt in der ‚Intention-to-treat' Population.

**Zeitplan**	*Patientenbezogen:*<br>Dauer der Intervention: 40 min<br>Nachbeobachtungsdauer: 12 Monate<br><br>*Studienbezogen:*<br>Die klinische Prüfung beginnt mit dem Datum der Randomisation des ersten Patienten. Die Dauer der klinischen Prüfung pro Patient beläuft sich über den gesamten Aufenthalt im Operationssaal (Intervention), auf der Intensivstation bis zum Zeitpunkt der Krankenhausentlassung. Über diesen Zeitraum werden studienbezogene Daten erhoben. Eine Nachbeobachtung erfolgt 30 Tage, 90 Tage und 12 Monate postoperativ.<br>Bei einer erwarteten mittleren Rekrutierungsrate von 1 bis 2 Patienten pro Woche pro Zentrum wird von einer Rekrutierungszeit von 2 bis 3 Jahren ausgegangen. Nach der Rekrutierungszeit wird die klinische Prüfung aufgrund der Nachbeobachtungszeit noch maximal 12 Monate fortdauern.<br>Die Endauswertung erfolgt in zwei Abschnitten;<br>• Analyse des Primären Endpunkts sowie aller bis Tag 30 erhobenen sekundären Endpunkte nach Krankenhausentlassung bzw. nach Tag 30 postoperativ des letzten eingeschlossenen Patienten und entsprechender Datenbereinigung<br>• Analyse der Einzelkomponenten des primären Endpunkts im Langzeit-Follow-up nach einjähriger Nachbeobachtung des letzten Patienten und entsprechender Datenbereinigung<br>Mit Erstellung des Abschlussberichts ist die klinische Prüfung formell beendet.

Flow Chart

	Präoperativ		OP	Postoperativ						Follow-up			
	V0		V1	V2	V3	V4	V5	V6	V7	V8	V9	V10	
**Untersuchung / Abfrage**	**Baseline**	**Randomisierung**	**Intervention/ Operation**	**0 - 24h postoperativ**	**24 - 48h postoperativ**	**48 - 72h postoperativ**	**72 - 96h postoperativ**	**ITS-Verlegung**	**KH-Entlassung**	**30±5 Tage postoperativ**	**90±15 Tage postoperativ**	**12±1 Monate postoperativ**	**Verdacht primäres Ereignis**
Einwilligungserklärung	●												
Ein- und Ausschlusskriterien	●												
Randomisierung		●											
Demografie	●												
Körperliche Untersuchung	●												
Anamnese / Begleiterkrankungen	●												
Begleitmedikation / Begleitbehandlung	●		●				●[1]	●					
Routinelabor	●		●[2]	●	●				●[3]				
Troponin T	●			●[4]	●								
DNA-Probe [5]	●												
12-Kanal-EKG	●			●		●							●
Lungenfunktion	●												
Herzkatheteruntersuchung [6]	●												
Echokardiographie [7]	●												
NIH Stroke Skale	●							●	●				●
Delir CAM-ICU	●			●	●	●	●						
Neurokognitive Testbatterie[8]	●								●		●		
Intervention			●										
Hämodynamik			●	●									
Bilanz[9]			●	●				●					
Katecholamine			●	●									
Transösophageale Echokardiographie [8]			●										
Operationsdaten			●										
Vorhofflimmern				●	●	●	●						
Beatmungszeit								●					
Komplikationen								●					
mechanische kardiale Unterstützungssysteme								●					
ITS-Verweildauer								●					
KH-Verweildauer									●				
Parameter des primären Endpunkts				●	●	●	●	●	●				●
Telefoninterview										●	●	●	
Unerwünschte Ereignisse[10]			●	●	●	●	●						

ITS: Intensivstation; KH: Krankenhaus; CAM-ICU: Confusion Assessment Method in the Intensive Care Unit; OP: Operation; V1-10: Visite 1-10

[1] nur postoperative Begleitmedikation zur Delirbehandlung

[2] tiefster Hämatokritwert und/oder Hämoglobinwert, höchster Glukosewert

[3] Maximaler Kreatininwert während Krankenhausaufenthalt

[4] nach 6, 12 und 24 h postoperativ

[5] Abnahme von DNA-Probe einmalig im Verlauf nach gesonderter Einwilligung

[6] Herzkatheterbefund max. 3 Monate alt

[7] Echokardiographiebefund max. 1 Monat alt

[8] Optional

[9] maximal innerhalb der ersten 7 Tage postoperativ auf Intensivstation

[10] Unerwünschte Ereignisse innerhalb 96 h postoperativ, die möglicherweise mit der Intervention (RIPC/Placebo) im Zusammenhang stehen

1 RATIONALE UND FRAGESTELLUNG

1.1 Hintergrund

Herzchirurgische Eingriffe mit dem Einsatz einer Herz-Lungen-Maschine (HLM) werden weltweit bei ca. 1,5 Millionen Patienten pro Jahr durchgeführt, und stellen damit eine der häufigsten operativen Eingriffe dar. In Abhängigkeit von verschiedenen Risikofaktoren, wie z.B. Notfalleingriff, Patientenalter, linksventrikuläre Herzfunktion, Komorbiditäten (Diabetes, peripher arterielle Verschlusskrankheit, Leber- und Niereninsuffizienz) und dem Ausmaß des operativen Eingriffs (Aortokoronare Bypass-Operation (ACB), Klappenoperation oder Kombinationseingriff) beträgt die perioperative Mortalität bei Patienten mit einem herzchirurgischen Eingriff heute noch bis zu 10%.[12,14,34,41,45] Trotz enormer Fortschritte bei der Narkoseführung und moderner Operationstechniken besteht zudem für das Herz ein relevantes Ischämierisiko, welches sowohl in eine rechts- als auch linksventrikuläre Funktionsstörung resultieren kann,[46,56] da während des intraoperativen Herzstillstands mit Klemmung der Aorta ascendens die Koronarien nicht mit Blut versorgt werden und den Herzmuskelzellen somit kein Sauerstoff mehr zugeführt wird.[5,55,59] Die Folge ist ein myokardiales Stunning, Apoptose und ein Ischämie-/Reperfusionssyndrom. Bei vielen Patienten ist daher postoperativ eine Erhöhung der Herzenzyme detektierbar. Im Hinblick auf postoperative Herzrhythmusstörungen stellt das akute Vorhofflimmern mit einer Inzidenz von 20-40% einen relevanten kardialen Endpunkt dar.[22]

Darüber hinaus kommt es gerade bei multi-morbiden Patienten postoperativ gehäuft zu neurologischen Funktionsstörungen,[4] die sich in 1 bis 4% als ischämischer Hirninfarkt darstellen,[6] aber wesentlich häufiger als postoperatives Delir[7,37] und postoperative kognitive Dysfunktion (POCD),[39,40] respektive Bewusstseinsstörungen, Verlust von kognitiven Fähigkeiten und Verwirrtheitszuständen auftreten. Je nach Patientenkollektiv entwickeln Intensivpatienten in bis zu 80 % ein Delir. Das Delir gilt als unabhängiger Prädiktor für erhöhte Mortalität auch nach Entlassung von der Intensivstation.[13,42,44] Ursächlich besteht wahrscheinlich eine Wechselwirkung zwischen verschiedenen prädisponierenden Risikofaktoren, wie z.B. Alkohol- und Nikotinabusus sowie vorbestehende kognitive Einschränkungen, und den präzipitierenden klinischen Auslösern. Zu den möglichen perioperativ auslösenden Faktoren zählen die Art des operativen Eingriffs, zerebrale Hypoxie und Ischämie, Schmerz, Infektionen, akute Stoffwechselentgleisung, Mangelzustände, hormonelle Störungen, akute vaskuläre Erkrankungen und medikamentös-toxische Probleme.

POCD wurde bei 30-70% der Patienten zum Zeitpunkt der Krankenhausentlassung, und bei 20-40% der Patienten 1 Jahr postoperativ beschrieben.[40] Die zu Grunde liegenden Mechanismen sind im Wesentlichen embolische Ereignisse, eine intraoperative Reduktion der zerebralen Perfusion, Ischämie-/Reperfusionsschäden und ein ‚Systemic Inflammatory Response Syndrome'. Die Folge sind verlängerte Liegezeiten im Krankenhaus, die mit einer schlechteren Wiedereingliederungsmöglichkeit in das häusliche Umfeld einhergehen.

Des weiteren wird der postoperative Erholungsprozess bei Patienten nach herzchirurgischen Eingriffen durch das hohe Risiko von Nierenfunktionsstörungen beeinflusst.[11,58,62] Die Inzidenz für akute Nierenschäden reicht in Abhängigkeit von der Definition von 1% bis 30%, wobei 1-5% der Patienten dialysepflichtig werden.[30,52] Verschiedene operations- und patientenabhängige Parameter, wie z.B. die Länge des kardio-pulmonalen Bypass, die Operationsart, eine reduzierte linksventrikuläre Pumpfunktion und vorbestehende Nierenfunktionsstörungen erhöhen das Risiko.[52] Insofern hätte ein Therapieansatz, welcher die Widerstandsfähigkeit der Niere erhöht, den perioperativen Ischämie-/Reperfusionsschaden zu überstehen, wichtige klinische Relevanz.

1.2 Darstellung der Studientherapie

Klinische Beobachtungen lieferten erste Hinweise darauf, dass Patienten mit schwerer Angina pectoris bei gleichem Schweregrad einen Herzinfarkt häufiger überleben als Patienten, bei denen es ohne Vorwarnung zum Verschluss des Koronargefäßes gekommen ist.[21,57] Unterstützung findet diese These in zahlreichen experimentellen und klinischen Studien, in denen ein oder mehrere kurze Zyklen von Ischämie/Reperfusion am gleichen Organ kurz vor (Präkonditionierung) oder nach (Postkonditionierung) einem länger dauernden Gefäßverschluss mit anschließender Reperfusion die Infarktgröße reduzieren konnten.[26,27,60] Dem liegt ein angeborener Schutzmechanismus zu Grunde, der mit einer gesteigerten Toleranz gegenüber einem nachfolgenden ischämischen Ereignis einhergeht. Da die direkte ischämische Präkonditionierung am gleichen Organ mit dem Risiko einer zusätzlichen ischämie-bedingten Organschädigung einhergeht, konnte sich die ursprüngliche Idee der ischämischen Präkonditionierung direkt am Zielorgan im klinischen Alltag nicht durchsetzen. Alternativ konnte tierexperimentell und klinisch gezeigt werden, dass verschiedene Pharmaka, insbesondere auch volatile Anästhetika, ähnliche protektive Effekte im Rahmen einer perioperativen Prä- und Postkonditionierung bewirken können, wenngleich der klinische Nutzen in aktuellen Metaanalysen weiterhin umstritten ist.[29,59]

Umso mehr stellt die neue Technik der ischämischen Fern (*Remote*)- Präkonditionierung (RIPC; remote ischaemic preconditioning) an weiter entfernten Organen eine viel

versprechende Alternative dar. Mit Hilfe dieser Methode wurden in verschiedenen experimentellen Untersuchungen Gewebe wie Darm, Niere oder Skelettmuskulatur einer wiederholten kurzzeitigen Ischämie ausgesetzt, wodurch wiederum neurohumorale Faktoren wie Adenosin, Opioide, Bradykinin und Calcitonin Gene Related Protein freigesetzt wurden.[18] Auf diese Weise können endogene Protektionsmechanismen aktiviert werden, die eine Toleranz gegenüber einem nachfolgenden ischämischen Ereignis im gesamten Organismus induzieren und folglich auch den myokardialen Schaden nach einem längeren Koronarverschluss verringern.[19,49,61] Ebenso gibt es bereits erste experimentelle Hinweise, dass die RIPC den neuronalen Zellschaden nach einer zerebralen Ischämie reduzieren kann, wenngleich die Mechanismen hierfür noch unbekannt sind.[10,51,63] Kharbanda und Kollegen[25] konnten am Modell der Unterarmischämie mit konsekutiver Endotheldysfunktion durch die RIPC am kontralateralen Arm bei gesunden Probanden eine Abschwächung des Ischämie-/Reperfusionsschadens und der induzierten Endothelfunktionsstörung aufzeigen.

Britische Kardiologen veröffentlichten ihre „Proof of principle"-Studie im *Lancet*, in der eine nicht-invasive absichtlich induzierte wiederholte Ischämie am Arm mit Hilfe einer Blutdruckmanschette in drei Fünf-Minuten-Zyklen bei 57 Patienten mit elektiver ACB-Operation den postoperativen Troponin I Anstieg, respektive myokardialen Zellschaden, um 43 Prozent reduzierte.[17] Ähnlich positive Ergebnisse konnten Cheung und Kollegen[8] bei 17 Kindern nach Korrektur von angeborenen Herzfehlern erzielen. Im Gegensatz dazu konnten Rahman et al. vor kurzem bei insgesamt 162 Patienten mit elektiver ACB-Operation keinen signifikanten Vorteil durch RIPC im Hinblick auf die Freisetzung von Troponin T, Katecholamindosis sowie Nieren- und Lungenschädigung finden.[50] Interessanterweise erfolgte in dieser Studie eine doppelte Verblindung durch das Aufpumpen einer Blutdruckmanschette am Patientenarm (RIPC-Gruppe) oder an einem Dummy-Arm (Kontroll-Gruppe). Die Arbeitsgruppe um Ali et al.[3] bestätigte wiederum den protektiven Nutzen der RIPC bei 82 Patienten mit elektiv abdomineller Bauchaortenaneurysma-Operation. Hoole und Kollegen[20] zeigten vor kurzem, dass Remote-Präkonditionierung die Freisetzung von Troponin I auch bei Patienten nach einem elektiven Herzkathetereingriff verringert, und insbesondere die Inzidenz kardiovaskulärer Ereignisse als relevanten klinischen Endpunkt innerhalb von 6 Monaten nach dem Eingriff reduziert. Der genaue Mechanismus für diesen Langzeiteffekt ist bisher noch unbekannt, aber zumindest für die klassische ischämische Präkonditionierung wurden bereits thrombozyten-inhibierende und anti-thrombotische Effekte beschrieben,[32] die möglicherweise vulnerable Plaques stabilisieren und die Endothelfunktion verbessern.[25]

Die publizierten Fallzahlen sind derzeit aber noch ungenügend, um daraus evidenzbasierte Rückschlüsse für die klinische Praxis ziehen zu können.

Eine prospektiv randomisierte, kontrollierte Studie, die Patienten einschließt, welche sich einem elektiven herzchirurgischen Eingriff (ACB, Herzklappenersatz, Aorta ascendens Ersatz, Kombinationseingriff) unterziehen müssen, fehlt derzeit. Zudem wurde der Einfluss der RIPC bisher noch nicht hinsichtlich relevanter klinischer Endpunkte wie Überlebensrate, Myokardinfarktrate, Nierenfunktion, Schlaganfallrate, Intensiv- und Krankenhaus-verweildauer, Delir und POCD untersucht.

Es soll die **Haupthypothese** getestet werden, dass RIPC das Risiko für das Auftreten von kardialen, zerebralen und renalen Ereignissen definiert als kombinierter Endpunkt aus Mortalität, Myokardinfarkt, Schlaganfall und/oder akutes Nierenversagen bis zum Zeitpunkt der Krankenhausentlassung (max. bis 14 Tage postoperativ bei längerem Krankenhausaufenthalt) um 1/3 reduziert.

Folgende **Nebenhypothesen** sollen getestet werden: RIPC verbessert die linksventrikuläre Pumpfunktion und reduziert das Auftreten der einzelnen Komponenten des kombinierten Endpunktes zu den Zeitpunkten Krankenhausentlassung sowie 30 Tage, 90 Tage und 12 Monate postoperativ. Darüber hinaus reduziert RIPC die Intensivstations- und Krankenhausverweildauer, den Myokardschaden, den Nierenschaden, die Häufigkeit von neu aufgetretenem Vorhofflimmern sowie von Delir und POCD.

1.3 Nutzen-Risiko Abschätzung

In der Interventionsgruppe wird die gegenwärtige Standardtherapie um RIPC erweitert. Die Patienten der Kontrollgruppe erhalten die derzeit übliche Standardtherapie.

Für RIPC konnte in zahlreichen experimentellen und klinischen Studien eine Reduktion des myokardialen Schadens aufgezeigt werden. Darüber hinaus beeinflusst RIPC möglicherweise die Morbidität und Mortalität günstig. Nach der gegenwärtigen Studienlage wäre somit ein Interventionsvorteil möglich, wenn Patienten mit einem elektiven herzchirurgischen Eingriff eine RIPC erhalten würden (siehe auch Kap. 1.2).

Das wiederholte Aufpumpen der Blutdruckmanschette für jeweils 5 Minuten kann als sicher angesehen werden. Da die Intervention nach der Narkoseeinleitung erfolgt, ist ein zusätzliches Schmerz- oder Stressempfinden durch die eigentliche Intervention unwahrscheinlich. Nebenwirkungen sind bisher nicht bekannt geworden. Das potentielle Risiko für Thrombose, Plaqueruptur oder Embolisation wird auch bei Patienten mit vorbestehender Arteriosklerose an den oberen Extremitäten als gering eingeschätzt.

Andererseits ist die Studienlage nicht ausreichend, um eine Empfehlung für die Anwendung von RIPC bei allen Patienten mit einem elektiven herzchirurgischen Eingriff auszusprechen.

Damit kann diese klinische Prüfung für ethisch vertretbar gehalten werden, da ein hohes Nutzenpotential (Senkung der Mortalität und Morbidität) einem niedrigen Risiko (nebenwirkungsarme Intervention) gegenüber steht.

Die Studienteilnahme hat keinen Einfluss auf die perioperative Betreuung. Das wiederholte Aufpumpen der Blutdruckmanschette am OP-Tag nimmt etwa 40 Minuten in Anspruch. Da dies nach der Narkoseeinleitung startet, und somit parallel zu den notwendigen Operationsvorbereitungen durchgeführt wird, wird die Narkose und Operation nicht verlängert. Die postoperative Therapie erfolgt nach klinischer Routine bei allen Patienten gleich.

Für die Laboranalysen werden den Patienten über mehrere Tage täglich Serum- und Plasmaproben entnommen. Diese Maßnahmen sind unbedenklich. Die gewonnene Menge an Blut stellt für den Patienten keine Gefahr dar. Eine Punktion ist nicht notwendig, da die Blutproben über Katheter entnommen werden können, die im Rahmen der perioperativen Überwachung und Behandlung gelegt werden. Die in dieser klinischen Prüfung gewonnenen Blutproben unterliegen strenger Maßnahmen zur Sicherung des Datenschutzes (siehe Kap. 14).

Im Rahmen eines Begleitprojektes wird darüber hinaus bei den Patienten, die zusätzlich DNA-Analysen zustimmen, einmalig Blut entnommen.

2 STUDIENZIELE

2.1 Primäres Ziel/ primärer Endpunkt

Das primäre Ziel der klinischen Prüfung ist zu untersuchen, ob RIPC einen Einfluss auf das Auftreten eines **kombinierten Endpunkts** (‚Composite Outcome’ **aus Mortalität, Myokardinfarkt, Schlaganfall und/oder akutes Nierenversagen**) innerhalb des Krankenhausaufenthaltes **bis zum Zeitpunkt der Krankenhausentlassung** (**maximal jedoch innerhalb der ersten 14 Tage postoperativ** bei längerem Krankenhausaufenthalt), hat. Zwecks Standardisierung der unterschiedlichen postoperativen Zeitpunkte wird im aktuellen Prüfplan durchgehend das Ende der Operation (= Ende der Hautnaht) mit dem Zeitpunkt *‘Null’* der postoperativen Phase definiert. Die Definition *‚Krankenhausentlassung’* bezieht sich auf die Entlassung des Patienten bei vollständiger Genesung aus dem lokalen Prüfzentrum zurück in den eigenen privaten Wohnbereich bzw. Pflegeeinrichtung bzw. Rehaklinik. Bei einer vorzeitigen Sekundärverlegung des Patienten aus dem Prüfzentrum in

ein peripheres Krankenhaus muss das primäre Prüfzentrum die Informationen zum primären Endpunkt schriftlich aus dem peripheren Haus einholen (z.B. Arztbrief).

I. **Mortalität**: Tod jeglicher Ursache

II. **Myokardinfarkt**: Die Diagnose eines nicht-tödlichen perioperativen Myokardinfarkts nach einem herzchirurgischen Eingriff wird wie folgt definiert:

i) ≤ 72 h postoperativ in Anlehnung an die *„Joint Task Force of the European Society of Cardiology/ American College of Cardiology/ American Heart Association for the Redefinition of Myocardial Infarction“* (Myokardinfarkt Typ 5):

1) Troponin T Anstieg größer als 5-fach des oberen Referenzwertes und

2 a) neue pathologische Q-Welle (oder neuer Linksschenkelblock) im EKG (detaillierte Kriterien im Anhang 19.2; 12-Kanal-EKG präoperativ, 24 und 72 h postoperativ)) oder

2 b) frischer Ischämienachweis in der Echokardiographie oder

2 c) falls vorhanden, postoperativ Koronarangiographie mit Nachweis von frischem Bypass- bzw. Koronararterienverschluss

ii) >72h postoperativ während des Krankenhausaufenthaltes sowie nach der Krankenhausentlassung erfolgt bei klinischen Zeichen eines Myokardinfarkts und nach Bestätigung durch einen Kardiologen die Diagnose eines Myokardinfarkts über die gebräuchlichen Kriterien (z.B. Troponin, CK-MB, ST-Hebung im EKG) in den lokalen Studienzentren an nicht vordefinierten Zeitpunkten, und/oder

iii) Hinweise auf einen Myokardinfarkt in der Autopsie.

III. **Schlaganfall**: Die Diagnose eines Schlaganfalls wird wie folgt definiert:

i) Während des primären Krankenhausaufenthaltes erfolgt die Diagnose bei jedem postoperativ neu aufgetretenen und länger als 24 h anhaltenden fokal/global neurologischen Defizit und einem Punktwert der NIH-Stroke Skala (NIHSS) ≥ 4 (NIHSS wird präoperativ, bei Intensivstations- und Krankenhausentlassung erhoben), und/oder

ii) Nach der Krankenhausentlassung erfolgt die Diagnose bei klinischen Zeichen eines Schlaganfalls und nach Bestätigung durch einen Neurologen über die gebräuchlichen Kriterien in den lokalen Studienzentren an nicht vordefinierten Zeitpunkten, und/oder

iii) Hinweise auf einen Schlaganfall in der Autopsie.

IV. **Akutes Nierenversagen**: Die Diagnose eines akuten Nierenversagens wird in Anlehnung an die RIFLE (Risk, Injury, Failure, Loss of kidney function, and End-stage kidney disease) – Klassifikation wie folgt definiert:

i) Maximaler postoperativer Kreatininwert während des Krankenhausaufenthaltes im Vergleich zum präoperativen Wert ≥ 2-fach erhöht (=RIFLE – Injury), und/oder

ii) Urinausscheidung ≤ 0,5 ml/kg/h über ein Zeitintervall von 12 h (=RIFLE – Injury), und/oder

iii) Nierenersatzverfahren (= RIFLE – Failure), und/oder

iv) Hinweise auf Akutes Nierenversagen in der Autopsie.

2.2 Sekundäre Ziele/ sekundäre Endpunkte

In den sekundären Zielen der klinischen Prüfung soll untersucht werden,

1. ob und in wie weit die Intervention die **kumulative Dauer der postoperativen mechanischen invasiven Beatmung** und evt. **nicht-invasiven Beatmung bis zum Zeitpunkt der Entlassung von der Intensivstation/ Intermediate Care Unit (maximal jedoch 30 Tage postoperativ)** günstig beeinflusst.

2. ob und in wie weit die Intervention die **postoperative Gesamtverweildauer auf der Intensivstation/ Intermediate Care Unit** (Zeitintervall zwischen Ende der Operation (= Ende der Hautnaht) und Verlegung auf Normalstation) **und im Krankenhaus** (Zeitintervall zwischen Ende der Operation und Entlassung des Patienten aus dem lokalen Prüfzentrum zurück in den privaten Wohnbereich bzw. Pflegeeinrichtung bzw. Rehaklinik) günstig beeinflusst.

3. ob und in wie weit die Intervention den Myokardschaden (**kardiales Troponin T i. S. präoperativ, 6, 12, 24 und 48 Stunden postoperativ)** günstig beeinflusst. Der Parameter wird unabhängig vom Intensivstationsaufenthalt an den definierten Zeitpunkten erhoben.

4. ob und in wie weit die Intervention die Nierenfunktion (**Kreatinin i. S. präoperativ, 24 und 48 Stunden postoperativ**; sowie **maximaler postoperativer Kreatininwert** während des Krankenhausaufenthaltes) günstig beeinflusst. Der Parameter wird unabhängig vom Intensivstationsaufenthalt an den definierten Zeitpunkten erhoben.

5. ob und in wie weit die Intervention die Überlebensrate und die **Häufigkeit der Einzelkomponenten des kombinierten Endpunktes** zum Zeitpunkt der **Krankenhausentlassung (maximal 14 Tage)** und **nach 30 Tagen, 90 Tagen und 12 Monaten postoperativ** günstig beeinflusst.

6. ob und in wie weit die Intervention das **Auftreten von Delir 24, 48, 72 und 96 Stunden postoperativ** anhand eines standardisierten Fragebogens (CAM-ICU; „Confusion Assessment Method for the Intensive Care Unit“) beeinflusst.[28] Der CAM-ICU wird unabhängig vom Intensivstationsaufenthalt an den definierten Zeitpunkten benutzt.
7. ob und in wie weit die Intervention den Einsatz von **Medikamenten zur Delirbehandlung** (z.B. Haloperidol, Promethazin, Lorazepam, Risperidon, Clonidin) **bis 96 h postoperativ** beeinflusst
8. ob und in wie weit die Intervention den i) **intraoperativen** und ii) **< 24 h postoperativen kumulativen Katecholaminbedarf** günstig beeinflusst.
9. ob und wie weit die Intervention die Häufigkeit von **neu aufgetretenem Vorhofflimmern** innerhalb von **96 h postoperativ** reduziert.
10. ob und wie weit die Intervention die Häufigkeit von **mechanischen kardialen Unterstützungssystemen** (z.B. Intra-aortale Ballonpumpe (IABP), Extrakorporale Kreislaufunterstützung (ECLS)) **bis zum Zeitpunkt der ITS/IMC-Entlassung**, **maximal jedoch bis 30 Tage postoperativ** reduziert.
11. **Optional:** ob und in wie weit die Intervention die **myokardiale Dysfunktion intraoperativ** günstig beeinflusst (wird nur in ausgewählten Prüfzentren erhoben).
12. **Optional**: ob und in wie weit die Intervention die Häufigkeit von **POCD 5-7 Tage** und **90 Tage postoperativ** günstig beeinflusst (wird nur in ausgewählten Prüfzentren erhoben).

Im Begleitprojekt (Kap. 8) sollen DNA-Proben zunächst zentral gesammelt werden, um im weiteren Verlauf genom-weite Assoziationsstudien von single nucleotide polymorphisms (SNP) und/oder gezielten Analyse so genannter Kandidatengene und postoperativen Komplikationen (z.B. Delir, Sepsis, akutes Nierenversagen, akutes Vorhofflimmern) zu untersuchen.

3 STUDIENBESCHREIBUNG

3.1 Studiendesign

Die klinische Prüfung wird als multizentrische, prospektive, randomisierte Untersuchung durchgeführt. Patienten, behandelnde Ärzte, Prüfer und Datenmanager sind hinsichtlich der

Intervention (RIPC vs. Placebo) verblindet. Die Anwendung der Intervention erfolgt durch eine Studienassistentin/ Pflegekraft unverblindet.

3.2 Personelle und technische Anforderungen an Prüfzentren

Die Studienleitung führt gemeinsam mit dem ZKS Leipzig – KKS nach individueller Rücksprache mit den Prüfzentren eine Pre-study-Visite durch. Hierbei wird die klinische Prüfung vorgestellt und die Eignung der Prüfzentren überprüft. Jedes Prüfzentrum erhält vor Beginn der klinischen Prüfung den Prüfplan ausgehändigt und bestätigt durch Unterschrift, dass die Infrastruktur zur Durchführung der klinischen Prüfung an dem Prüfzentrum vorhanden ist. Die Qualifikation des Prüfzentrums und der Prüfer wird dazu vor Beginn der klinischen Prüfung in einem Formblatt erfasst. Der Leiter des Prüfzentrums muss ein in der perioperativen Betreuung herzchirurgischer Patienten erfahrener Arzt sein.

Die Studienassistenten/ Pflegekräfte werden im Rahmen des Initiierungsbesuchs für Randomisation und Intervention geschult. Die Schulung der Prüfer berücksichtigt insbesondere die klinischen Inhalte und Untersuchungsmethoden (Prüfplan, Working Instructions, EKG, NIH Stroke Scale, CAM-ICU, neurokognitive Testung, Echokardiographie etc.).

3.3 Teilnehmende Zentren und Zahl der Patienten

Die klinische Prüfung wird als multizentrische klinische Prüfung durchgeführt. Es nehmen 10 Zentren an der klinischen Prüfung teil (siehe Anhang 19.4). Insgesamt sollen 2070 Patienten in die klinische Prüfung eingeschlossen werden.

3.4 Erwartete Studiendauer

Die klinische Prüfung beginnt mit dem Datum der Randomisation des ersten Patienten.

Die Dauer der klinischen Prüfung pro Patient beläuft sich über den gesamten Aufenthalt im Operationssaal (Intervention), auf der Intensivstation bis zum Zeitpunkt der Krankenhausentlassung. Über diesen Zeitraum werden studienbezogene Daten erhoben. Bei längerem Aufenthalt auf der Intensivstation und im Krankenhaus werden Daten zum primären Endpunkt maximal 14 Tage postoperativ und Daten zu den sekundären Endpunkten maximal 30 Tage postoperativ erhoben. Eine Nachbeobachtung erfolgt 30 Tage, 90 Tage und 12 Monate postoperativ.

Optional erfolgt an einzelnen Prüfzentren zusätzlich die Erhebung der neurokognitiven Testbatterie zu den Zeitpunkten: i) präoperativ, ii) 5 -7 Tage postoperativ sowie iii) 90 ± 15 Tage postoperativ (siehe Prüfarztordner).

Bei einer erwarteten mittleren Rekrutierungsrate von 1 bis 2 Patienten pro Woche pro Zentrum wird von einer Rekrutierungszeit von 2 bis 3 Jahren ausgegangen. Nach der Rekrutierungszeit wird die klinische Prüfung aufgrund der Nachbeobachtungszeit noch maximal 12 Monate fortdauern.

Es ist geplant, die Datenerfassung 3 Monate nach Abschluss der Follow-up Phase des letzten Patienten zu beenden und die Datenbank zu schließen. Mit Erstellung des Abschlussberichts ist die klinische Prüfung formell beendet.

3.5 Studienabbruch

3.5.1 Abbruch der Studie in einem Prüfzentrum

Die Studie kann in einem Prüfzentrum abgebrochen werden, wenn

- es nicht den technischen Anforderungen des Prüfplans genügt,
- die Studiendurchführung nicht dem Prüfplan genügt,
- die Datenqualität unzureichend ist,
- es unzureichend rekrutiert.

Über den Ausschluss entscheidet die Studienleitung, gegebenenfalls in Abstimmung mit dem Biometriker. Prüfärzte oder Prüfzentren, die nicht mehr an der Studie teilnehmen, müssen unverzüglich den Studienleiter über ihre Entscheidung informieren. Die Entscheidung soll immer auch begründet werden. Die Weiterbehandlung noch in der Studie befindlicher Patienten wird mit der Studienleitung abgesprochen.

3.5.2 Abbruch der gesamten Studie bzw. einzelner Studienarme

Die Studie kann durch die Studienleitung vorzeitig abgebrochen werden im Falle von

- schwerwiegenden unerwünschten Ereignissen
- unerwarteten, unerwünschten Ereignissen, die zu einer konsekutiv geänderten Nutzen-Risiko-Bewertung führen
- weiteren Änderungen in der Nutzen-Risiko-Bewertung
- nicht ausreichender Rekrutierungsrate.

Das Independent Data Monitoring and Safety Committee (IDMC) wird einmal jährlich ein Ergebnis zu den vorgelegten Analysen formulieren (siehe auch Abschnitt 13.4). Das IDMC kann auf Basis der Analysen der Studienleitung den Abbruch der gesamten klinischen Prüfung bzw. einzelner Arme empfehlen. Die endgültige Entscheidung über den Abbruch der Studie liegt bei der Studienleitung.

4 STUDIENPOPULATION

4.1 Einschlusskriterien

Für den Einschluss in die klinische Prüfung müssen alle Einschlusskriterien erfüllt sein.

1. elektiver herzchirurgischer Eingriff an der Herz-Lungen-Maschine, insbesondere Aortokoronare Bypassoperation, Aortenklappenersatz, Mitralklappenersatz oder -rekonstruktion, Aorta ascendens Ersatz oder Kombinationseingriff
2. Alter ≥ 18 Jahre
3. Schriftliche Einwilligungserklärung des Patienten/der Patientin

4.2 Ausschlusskriterien

Für den Einschluss in die klinische Prüfung darf kein Ausschlusskriterium zutreffen.

Ausschlusskriterien in Bezug auf den Eingriff:

1. Off-pump Verfahren
2. Minimal-invasive Eingriffe über laterale Thorakotomie
3. Aorta descendens Ersatz
4. Gleichzeitige Carotis-TEA
5. Selektive antegrade Hirnperfusion während HLM-Stillstand
6. Voroperation an Herz/ Aorta, da alte Verwachsungen im Thoraxbereich das operative Vorgehen sowie die Komplikationsrate beeinflussen

Ausschlusskriterien in Bezug auf die kardiale Anamnese:

7. Schwere linksventrikuläre Funktionsstörung (EF≤30% mittels Coronarangiographie und/oder Echokardiographie)

8. Chronisches Vorhofflimmern oder intermittierendes Vorhofflimmern innerhalb der letzten 6 Monate präoperativ
9. Medikamentöse Einnahme von Digitalis-Präparaten, Amiodaron und/oder Antiarrhythmika Klasse I (z.B. Ajmalin, Flecainid, Propafenon, Tocainid)
10. Implantierter Herzschrittmacher oder Defibrillator
11. Myokardinfarkt innerhalb der letzten 7 Tage präoperativ
12. Instabile Angina pectoris innerhalb der letzten 24 h präoperativ mit bereits präoperativen positiven Troponin T Anstieg

Ausschlusskriterien in Bezug auf weitere Begleiterkrankungen:

13. Schwere Niereninsuffizienz (Kreatinwert i.S. > 2,0 mg/dl (> 176 µmol/l))
14. Schwere Leberinsuffizienz (spontan INR >1,8 und/oder Bilirubin i. Serum > 8,8 mg/dl (> 150 µmol/l) und/oder nach ärztlicher Einschätzung)
15. Schwere Lungenerkrankung (SpO_2<85% bei Raumluft und/oder FeV1≤ 50% und/oder nach ärztlicher Einschätzung)
16. Schwerer Alkoholabusus
17. Schlaganfall innerhalb der letzten 2 Monate präoperativ
18. NIH Stroke Scale > 2 präoperativ
19. Schwere neurologische Erkrankungen (z.B. Morbus Parkinson, Multiple Sklerose, Morbus Alzheimer, präoperatives Delir und/oder nach ärztlicher Einschätzung)
20. Akute Infektionen mit Antibiotikatherapie innerhalb der letzten 7 Tage präoperativ
21. Medikamentöse Einnahme von Sulfonylharnstoffen (z.B. Glibenclamid) und Nicorandil, die innerhalb von 24 h präoperativ nicht pausiert wurden, da für beide Medikamente Interaktionen mit potentiellen Konditionierungsmechanismen gezeigt wurden
22. Peripher arterielle Verschlusskrankheit Stadium IV
23. Heparin-induzierte Thrombozytopenie Typ II

Weitere Ausschlusskriterien:

24. Teilnahme an anderen klinischen Prüfungen innerhalb der letzten 30 Tage
25. Infauste Prognose aufgrund von Nebenerkrankungen (z.B. Überlebenswahrscheinlichkeit < 12 Monate)

26. Weitergehende Beziehung zum Prüfer (z.B. Mitarbeiter, Verwandte, Kollegen)
27. Frauen während der Schwangerschaft und Stillzeit
28. Sprach-/ Verständigungsprobleme im Rahmen der Aufklärung
29. Mangelnde Kooperationsbereitschaft (Compliance)

4.3 Begründung für den Einschluss spezieller Populationen

Entfällt.

5 INDIVIDUELLER STUDIENABLAUF

5.1 Prüfung der Ein- und Ausschlusskriterien

Die Prüfung der Ein- und Ausschlusskriterien potentieller Studienpatienten ("Screening") erfolgt anhand einer vorgefertigten Checkliste:

- Alle Patienten, die die Einschlusskriterien (d.h. elektiver herzchirurgischer Eingriff an der Herz-Lungen-Maschine und Alter ≥ 18 Jahre erfüllen, werden in einer Screeningliste (Screening-Log; siehe Prüfarztordner) pro Prüfzentrum dokumentiert.
- Wenn ein Patient alle Einschlusskriterien erfüllt und keine Ausschlusskriterien vorliegen ("eligible"), ist im Screening-Log zu dokumentieren, ob ein Einschluss in die klinische Prüfung erfolgt ist ("enrolled").
- Falls der Patient nicht in die klinische Prüfung eingeschlossen wird, so sind im Screening-Log mindestens 1 Ausschlusskriterium - bei möglicher Mehrfachnennung max. 4 relevante Ausschlusskriterien - zu dokumentieren. Jeder einschlussfähige Patient ist zu dokumentieren.

Im Rahmen der Überprüfung der Ein- und Ausschlusskriterien dokumentiert der Prüfarzt, dass der Patient aktuell und auch in den vorangegangenen 30 Tagen nicht an einer anderen klinischen Prüfung oder einem anderen Forschungsprojekt teilnimmt/ teilgenommen hat. Hierfür wird die Patientenakte sorgfältig auf entsprechende Angaben überprüft. Es wird dem Patienten erklärt, dass der Patient nicht an einer anderen klinischen Prüfung oder einem anderen Forschungsprojekt teilnehmen darf, da eine gleichzeitige Teilnahme an mehreren klinischen Prüfungen oder Forschungsprojekten unkalkulierbare Risiken für den Patienten darstellen können.

5.2 Patientenaufklärung und -einwilligung

5.2.1 Aufklärung und Einwilligungserklärung

Vor Aufnahme in die klinische Prüfung (Randomisation) wird jeder Patient vom behandelnden Arzt mündlich und schriftlich über Wesen, Bedeutung, Ziele, Tragweite, erwartete Vorteile und mögliche Risiken der klinischen Prüfung informiert.

Der Prüfer händigt dem Patienten vor bzw. bei der mündlichen Aufklärung die schriftliche Patienteninformation aus. Dem Patienten muss ausreichend Zeit zum Lesen der Patienteninformation und die Gelegenheit, offene Fragen mit dem Arzt zu klären, gegeben werden. Anschließend ist dem Patienten eine angemessen Bedenkzeit vor der Einwilligung zur Teilnahme an der klinischen Prüfung zu gewähren.

Entscheidet sich der Patient für eine Teilnahme an der klinischen Prüfung, wird die Einwilligungserklärung in zweifacher Ausfertigung unterzeichnet. Ein Exemplar erhält der Patient, das andere verbleibt beim Prüfer (abzulegen im Prüfarztordner).

In der Einwilligungserklärung willigt der Patient nicht nur zur Teilnahme an der klinischen Prüfung ein, sondern genehmigt außerdem die Einsicht in seine Originalkrankenunterlagen durch den Monitor und weitere befugte Personen (Inspektoren und Auditoren). Die Einwilligung des Patienten muss sich ausdrücklich auch auf die Erhebung und Verarbeitung von Angaben über die Gesundheit sowie der Kontaktaufnahme des Studienleiters zum behandelnden Hausarzt beziehen. Deshalb ist der Patient explizit über Zweck und Umfang der Erhebung und die Verwendung personenbezogener Daten, insbesondere von Gesundheitsdaten zu informieren.

5.2.2 Vorgehensweise bei der Entnahme von genetischem Material (DNA-Probe)

Im Rahmen der klinischen Prüfung soll einmalig eine DNA-Probe für genetische Untersuchungen entnommen werden. Die DNA-Probe wird zur Durchführung genomweiter "Single Nucleotide Polymorphism (SNP)"-Untersuchungen (Hochdurchsatzverfahren mittels SNP-Array) und/oder gezielten Analyse so genannter Kandidatengene verwendet. Ziele der genetischen Untersuchungen sind die Identifikation von genetischen Faktoren im pathophysiologischen Zusammenhang der Intervention und der postoperativen Komplikationen.

Der Patient hat die Möglichkeit, unabhängig von seiner Zustimmung zur Teilnahme an der klinischen Prüfung der Entnahme der DNA-Probe zu widersprechen. Die Entnahme der DNA-Probe ist nicht zeitkritisch.

5.2.3 Rücknahme der Einwilligung

Patienten können jederzeit und ohne Angabe von Gründen ihre Einwilligung zurückziehen und die Studie abbrechen. Der Patient wird in einem solchen Fall gebeten, den Abbruchgrund zu nennen, jedoch darauf hingewiesen, dass er dies nicht tun muss. Die Information, dass, wann und wofür ein Patient randomisiert wurde, und dass und wann er seine Einwilligung zurückgezogen hat, muss in der Dokumentation erhalten bleiben.

Der Patient ist darüber zu informieren, dass im Falle eines Widerrufs der Einwilligung die gespeicherten Daten weiterhin verwendet werden dürfen.

5.3 Aufnahme in die Studie

5.3.1 Registrierung und Randomisierung

Registrierung und Randomisierung sowie Mitteilung des Randomisationsergebnisses erfolgt durch einen automatisierten internetbasierten Dienst, der durch das ZKS Leipzig - KKS zur Verfügung gestellt wird.

Die Registrierung kann erfolgen, sobald der Patient seine Einwilligung gegeben hat. Bei der Registrierung werden die Ein- und Ausschlusskriterien abgefragt. Wir empfehlen, die Registrierung kurz vor dem Operationstermin durchzuführen, damit die Ein- und Ausschlusskriterien zum Zeitpunkt der Operation gelten (Vgl. 5.3.4).

Die Randomisation erfolgt nach der Registrierung – ebenfalls möglichst zeitnah vor der Operation. Sie darf nur durch eine akkreditierte unverblindete Studienassistentin/ Pflegekraft erfolgen.

Die Randomisierung wird nach i) Zentren und nach ii) dem individuellen Risiko für die perioperative Mortalität anhand des *European System for Cardiac Operative Risk Evaluation* (EuroSCORE)[38] Internet-basiert stratifiziert. Der EuroSCORE schätzt das individuelle Operationsrisiko ab, innerhalb von 30 Tagen postoperativ zu versterben. Für die Randomisation werden die einzelnen Parameter des EuroSCORE abgefragt. Die Stratifizierung erfolgt danach anhand des durch die Datenbank berechneten EuroSCORE.

In der aktuellen Studie werden mit Hilfe des absoluten EuroSCORE bei einem cut-off Risikowert ≥ 6 „Hochrisikopatienten“, welche ungefähr 20% der Gesamtgruppe[15] darstellen, stratifiziert. Die einzelnen Komponenten des EuroSCORE sind im Anhang 19.5 aufgeführt.

Nach erfolgreicher Randomisation erfolgt die Mitteilung des Ergebnisses Internet-basiert verschlüsselt an die unverblindete Studienassistentin/ Pflegekraft. Ein Ausdruck des Randomisationsergebnisses wird für jeden einzelnen Patienten in einem gesicherten und nur

für die unverblindete Studienassistentin/ Pflegekraft zugänglichen Prüfarztordner abgeheftet. Nach Abschluss der Intervention erfolgt eine Bestätigung der Gruppenzuteilung und Dokumentation der Intervention per Fax mit Hilfe einer Rückantwortvorlage an das ZKS Leipzig – KKS.

5.3.2 Entnahme der DNA-Probe

Die Entnahme der DNA-Probe darf nur erfolgen, wenn in der, den Vorgaben der lokalen Ethikkommission entsprechenden Einwilligungserklärung des Patienten eine Zustimmung zur Entnahme der DNA-Probe gegeben wurde!

Für die Entnahme der DNA-Probe werden drei mit einem Barcode-Aufkleber versehene Blutröhrchen genutzt, die von der Biobank Popgen, Universitätsklinikum Schleswig-Holstein, Campus Kiel, als Komplettpaket inklusive Rücksendeumschlag und Begleitschein vorbereitet sind. Nach Blutentnahme sollen die 3 Blutröhrchen in dem beiliegenden Rücksendeumschlag an die Biobank Popgen, Universitätsklinikum Schleswig-Holstein, Campus Kiel, ungekühlt innerhalb von 2 Tagen nach Entnahme zurückgesendet werden. Zusätzliche Kennzeichnungen der DNA-Probe sind unzulässig! **Um nach Abschluss der Studie die klinischen Daten der Patienten mit den DNA-Analysen abzugleichen, muss ein weiterer Barcode-Aufkleber der Biobank Popgen in den CRF-Bogen geklebt werden.**

5.3.3 Verzögerung der geplanten Operation

Wird nach der Registrierung (unabhängig davon, ob der Patient bereits randomisiert wurde oder nicht) aus organisatorischen Gründen die Operation kurzfristig innerhalb eines Zeitfensters von maximal ≤7 Tagen verschoben, bleibt der Patient in der Studie, und wird nicht ausgeschlossen. Um potentielle neu aufgetretene Begleiterkrankungen und/oder kardiovaskuläre Ereignisse während der „Wartezeit" aber auszuschließen, werden spätestens am Operationstag vor der Intervention kurz die Ausschlusskriterien erneut geprüft.

5.3.4 Feststellung von Verletzungen der Ein- und Ausschlusskriterien

Zeitraum zwischen Registrierung und Intervention

Wird nach der Registrierung – aber vor der Intervention – festgestellt, dass unerwartet bei einem Patienten Ausschlusskriterien zutreffen (z.B. Änderung der Operationstechnik, akute Verschlechterung des Patienten, etc.), wird dieser Patient noch vor der Intervention wieder ausgeschlossen. Es erfolgt umgehend eine Meldung an das Datenmanagement des ZKS

Leipzig - KKS. Der betreffende Patient gehört nicht der Studienpopulation an, es erfolgt keine weitere Beobachtung oder Dokumentation im Rahmen der Studie.

Zeitraum nach Beginn der Intervention

Wird nachträglich - nach Beginn der Intervention - festgestellt, dass zum Zeitpunkt der Rekrutierung eines Patienten bereits eine Verletzung der Ein- und Ausschlusskriterien vorlag, so wird das Datenmanagement des ZKS Leipzig - KKS hierüber so schnell wie möglich informiert. Die Verletzung von Ein- und Ausschlusskriterien ist in dieser Situation aber **KEIN GRUND** für einen Abbruch der Studie bei dem betreffenden Patienten.

Es werden dann im Allgemeinen zwei Analysepopulationen differenziert:

(1) “Intention To Treat” = Auswertung aller Patienten wie randomisiert

(2) „Per Protocol“= Auswertung aller Patienten ohne relevante Prüfplanverletzungen.

In der Analysepopulation „Per Protocol“ würden die Patienten mit nachträglichen Verletzungen der Ein- und Ausschlusskriterien im Rahmen der Datenauswertung ausgeschlossen werden (siehe auch Abschnitt 7.5.1).

5.4 Beschreibung des Studienablaufes

5.4.1 Visite 0 - Einschluss in die klinische Prüfung (Baseline)

- Patientenaufklärung und Einwilligungserklärung
- Ein- und Ausschlusskriterien
- Demographische Daten
- Familienanamnese
- Körperliche Untersuchung
- Anamnese und Begleiterkrankungen
- Begleitmedikation
- Routinelabor (max. 7 Tage alt; Glukose; Blutbild: Hämoglobin/ Hämatokrit, Thrombozytenanzahl, Leukozytenanzahl; Nierenfunktion: Kreatinin i. S.; Leberfunktion: Bilirubin i. S.; Gerinnungssystem: INR/ Quick, PTT)
- Studienbezogener Parameter: Troponin T i. S.
- DNA-Probe (falls Einwilligung vorliegend)
- 12-Kanal-EKG (bei klinisch stabilen Patienten max. 7 Tage alt)
- Lungenfunktion (soweit bereits durchgeführt)
- Herzkatheterbefund (max. 3 Monate alt)
- Echokardiographiebefund (max. 1 Monat alt)

- NIH Stroke Scale
- CAM-ICU Score
- Optional: Neurokognitive Testung

  Zur Evaluation der postoperativen neurokognitiven Dysfunktion (POCD) sollen die an dieser Untersuchung teilnehmenden Patienten (1) am Tag vor der Operation, (2) nach 5-7 Tagen und (3) nach 90±15 Tagen postoperativ mittels einer standardisierten Testbatterie in Anlehnung an ein „Consensus Statement“ speziell für herzchirurgische Patienten[37,53] untersucht werden (siehe Prüfarztordner).

5.4.2 Randomisation

Randomisierung sowie Mitteilung des Randomisationsergebnisses erfolgt durch einen automatisierten internetbasierten Dienst, der durch das ZKS Leipzig - KKS zur Verfügung gestellt wird (Vgl. 5.3.1)

5.4.3 Visite 1 – Intervention und Operation

- Narkose und Management der Herz-Lungen-Maschine

  Bei allen Patienten wird die Narkose standardisiert als **totalintravenöse Anästhesie**, z.B. mit Propofol (Induktion: 1,5-2 mg/kg; Erhaltung 3-6 mg/kg/h) und Sufentanil (Induktion: 0,5 µg/kg; Erhaltung: 0,5-1,5 µg/kg/h) durchgeführt. **Volatile Anästhetika sollen nicht verabreicht werden.** Die Instrumentierung beinhaltet mindestens einen arteriellen und einen zentralvenösen 3-Lumen-Katheter.

  Um im Rahmen der Studie den Einfluss von Kortison auf die primären und sekundären Studienziele zu minimieren, sollte eine prophylaktische und breit angewandte intraoperative Gabe von niedrig dosiertem Hydrocortison (<200 mg/24 h) zurückhaltend erfolgen, da für eine generelle Empfehlung die aktuelle Studienlage noch unzureichend ist. Im Einzelfall kann bei einem höheren intraoperativen Katecholaminbedarf je nach klinik-internem Standard die Kortisongabe erwogen werden.

  Folgende Zielparameter werden beim Management der Herz-Lungen-Maschine (HLM)/ des kardio-pulmonalen Bypass berücksichtigt:

  - pulsatiler Fluss von 2-3 l/min/m^2 (=80-130% des berechneten idealen Herzzeitvolumens),
  - mittlerer arterieller Blutdruck von 50–75 mm Hg
  - Hämatokrit von 25-30%,

- vorzugsweise milde Hypothermie bei 32-34℃ nasale Temperatur während der aortalen Klemmphase (alternativ auch übliches lokales Temperaturmanagement),
- bei Aorta ascendens Ersatz vorzugsweise tief hypothermer Herz-Kreislaufstillstand ohne selektive antegrade Hirnperfusion bei 18-30℃ nasale Temperatur (alternativ auch übliches lokales Temperaturmanagement) und
- vorzugsweise kalte Blutkardioplegie nach Buckberg (alternativ auch übliche lokale Kardioplegielösung).

Die Patienten werden nach dem kardio-pulmonalen Bypass beatmet und sediert auf der Intensivstation/ Intermediate Care Unit überwacht. Bei stabilen Kreislaufverhältnissen, suffizienter Eigenatmung und adäquater Orientierung erfolgt die Verlegung auf Normalstation und nach 5-10 Tagen die Entlassung aus dem Krankenhaus.

Im Rahmen der perioperativen Blutzuckereinstellung wird mittels intermittierender/kontinuierlicher i.v. Gabe von Insulin ein Blutzuckerwert von 70 bis 160 mg/dL angestrebt (intraoperativ während milder Hypothermie Blutzuckerwert <200 mg/dl).

- Intervention

Bei allen Studienpatienten wird nach der Narkoseeinleitung an einem Oberarm eine manuelle Blutdruckmanschette angelegt. Zusätzlich wird ein Dummy-Arm mit einer zweiten manuellen Blutdruckmanschette neben den Interventionsarm gelagert, die dann zusammen abgedeckt werden. Durch eine Studienassistentin/ Pflegekraft erfolgt anschließend entsprechend dem Randomisationsergebnis die Bedienung einer der beiden Blutdruckmanschetten:

Gruppe 1 - Ischämische Fern *(Remote)*- Präkonditionierung: Nach der Narkoseeinleitung wird mit der **Blutdruckmanschette am Oberarm** des Patienten für 5 Minuten ein Druck appliziert, der mindestens 30 mm Hg höher als der invasiv gemessene, systolische arterielle Blutdruck des Patienten ist und nicht < 200 mm Hg liegt. Nach dieser Ischämiephase wird 5 Minuten pausiert. Dieses Procedere wird viermal wiederholt. Die Anzahl der Zyklen und Dauer der Ischämie/Reperfusionsphasen sind in der Literatur variabel beschrieben. In Anlehnung an Cheung et al.[8], Kharbanda et al.[24], Schmidt et al.[54] sieht die vorliegende Studie vier 5-minütige Zyklen vor.

Gruppe 2 – Kontrolle/ Placebo: Bei Patienten in der Kontrollgruppe werden nach der Narkoseeinleitung mit der zweiten Blutdruckmanschette an dem Dummy-Arm

entsprechend dem RIPC-Protokoll vier identische Zyklen appliziert – Wechsel von jeweils 5 Minuten Aufpumpen bis zu einem Druck von 200 mm Hg und 5 Minuten Pause.

Um die doppelte Blindung im Rahmen der Intervention nicht zu gefährden (Aufpumpen bei RIPC führt zum Verlust des arteriellen Pulssignals), sollte die invasive arterielle Blutdruckmessung am kontralateralen Arm und die Pulsoxymetrie am kontralateralen Arm bzw. peripher an Ohr oder Nase erfolgen. Alternativ muss in der Phase der Intervention am Interventionsarm das Monitoring deaktiviert sein.
Als **Sonderfall** gilt die Entnahme der A. radialis als aortokoronarer Bypass am kontralateralen Nicht-Interventionsarm. Bei diesen Patienten sollte die invasive arterielle Blutdruckmessung über einen Katheter in der A. femoralis und die Messung der Sauerstoffsättigung peripher an Ohr oder Nase erfolgen.

Die Verwendung von zwei abgedeckten Blutdruckmanschetten durch eine Studienassistentin/ Pflegekraft stellt sicher, dass bei jedem Patienten ein vergleichbares Aufpumpmanöver zu hören ist, und entsprechend des Randomisierungsergebnis die richtige Manschette bedient wird:

RIPC: aktives Aufpumpen der Blutdruckmanschette am Patientenoberarm
Kontrolle: aktives Aufpumpen der Blutdruckmanschette am Dummy-Arm[50]

Auf diese Art und Weise gelingt eine ‚echte doppelte Verblindung' von Patient, Operateur, Anästhesist, Intensivmediziner und Datenmanager.

- Operation
  - Dokumentation von Operationsdaten (HLM (Bypass)-Zeit, Aortenklemmzeit, HLM-Stillstandzeit bei Aorta ascendens Ersatz, tiefste Temperatur während der Aortenklemmzeit, HLM-Fluss, Art der Kardioplegielösung)
  - Dokumentation der Hämodynamik (Mittlerer arterieller Blutdruck und Herzfrequenz; vor Narkoseeinleitung, nach Hautschnitt, und 15, 30 und 60 Minuten nach Ende der HLM (post-HLM))
  - Dokumentation der Bilanz (Einfuhr: Gesamtmenge von kristalloiden und kolloidalen Infusionslösungen, Erythrozytenkonzentrat, Fresh Frozen Plasma, Thrombozytenkonzentrat, Kardioplegie und HLM-Primingvolumen; Ausfuhr: Gesamt-Blutverlust, Gesamt-Urinausscheidung)
  - Katecholaminverbrauch (Gesamtmenge von Noradrenalin, Adrenalin, Dobutamin, Vasopressin, Enoximon, Milrinon)

- Begleitmedikation (Gesamtmenge von Insulin, Kortison, Antiarrhythmika, Diuretika)
- Begleitbehandlung (mechanische Kreislaufunterstützung)
- Dokumentation von intraoperativen Routinelaborwerten (tiefster Hämatokritwert und/oder Hämoglobinwert, höchster Glukosewert)
- Dokumentation von Verlegung (Intensivstation, Intermediate Care Unit, Aufwachraum)
- Optional: Transösophageale Echokardiographie 1) nach Narkoseeinleitung vor der HLM und 2) 30±5 min nach Ende der HLM (post-HLM) erhoben
- Die mittels der transösophagealen Echokardiographie zu erhebenden Funktionsparameter sind im Anhang 19.6 detailliert aufgeführt. Insbesondere sollen Parameter sowohl der systolischen (linksventrikuläre Ejektionsfraktion ($LVEF_{Simpson}$, $LVEF_{Teich}$, Fractional $shortening_{Teich}$)) als auch der diastolischen Herzfunktion (E/A Ratio, frühe Dezelerationszeit, Myocardial Performance Index) analysiert werden.
- Unerwünschte Ereignisse (AE/ SAE; siehe Kap. 6)

5.4.4 Visite 2 (0 bis 24 h postoperativ)

- Dokumentation der Hämodynamik (Mittlerer arterieller Blutdruck und Herzfrequenz 24 h postoperativ)
- Dokumentation der Bilanz (Einfuhr: Gesamtmenge von kristalloiden und kolloidalen Infusionslösungen (Art der kolloidalen Infusionslösung), parenterale und enterale Ernährung, Erythrozytenkonzentrat, Fresh Frozen Plasma, Thrombozytenkonzentrat; Ausfuhr: Gesamt-Blutverlust, Gesamt-Urinausscheidung, Drainageverluste) innerhalb der ersten 24 h postoperativ
- Katecholaminverbrauch (Gesamtmenge von Noradrenalin, Adrenalin, Dobutamin, Vasopressin, Enoximon, Milrinon) innerhalb der ersten 24 h postoperativ
- Routinelabor (Glukose; Blutbild: Hämoglobin/ Hämatokrit, Thrombozytenanzahl, Leukozytenanzahl; Nierenfunktion: Kreatinin i. S.; Gerinnungssystem: INR/ Quick, PTT) 24 h postoperativ
- Troponin T 6, 12, und 24 h postoperativ
- 12-Kanal-EKG 24 h postoperativ
- CAM-ICU (siehe Anlage 19.1.) 24 h postoperativ
- Neu aufgetretenes Vorhofflimmern postoperativ
- Unerwünschte Ereignisse (AE/ SAE; siehe Kap. 6)
- Parameter des kombinierten primären Endpunktes

5.4.5 Visite 3 (24 bis 48 h postoperativ)

- Routinelabor (Glukose; Blutbild: Hämoglobin/ Hämatokrit, Thrombozytenanzahl, Leukozytenanzahl; Nierenfunktion: Kreatinin i. S.; Gerinnungssystem: INR/ Quick, PTT) 48 h postoperativ
- Troponin T bei 48 h postoperativ
- Neu aufgetretenes Vorhofflimmern postoperativ
- CAM-ICU (siehe Anlage 20.1.) 48 h postoperativ
- Unerwünschte Ereignisse (AE/ SAE; siehe Kap. 6)
- Parameter des kombinierten primären Endpunktes

5.4.6 Visite 4 (48 bis 72 h postoperativ)

- 12-Kanal-EKG 72 h postoperativ
- Neu aufgetretenes Vorhofflimmern postoperativ
- CAM-ICU (siehe Anlage 20.1.) 72 h postoperativ
- Unerwünschte Ereignisse (AE/ SAE; siehe Kap. 6)
- Parameter des kombinierten primären Endpunktes

5.4.7 Visite 5 (72 bis 96 h postoperativ)

- Neu aufgetretenes Vorhofflimmern postoperativ
- CAM-ICU (siehe Anlage 20.1.) 96 h postoperativ
- Dokumentation der postoperativen Begleitmedikation zur Delirbehandlung (z.B. Haloperidol, Promethazin, Lorazepam, Risperidon, Clonidin) bis 96 h postoperativ
- Unerwünschte Ereignisse (AE/ SAE; siehe Kap. 6)
- Parameter des kombinierten primären Endpunktes

5.4.8 Visite 6 (Verlegung von der ITS)

- Gesamte ITS-Verweildauer (unabhängig ob primäres Prüfzentrum, peripheres Krankenhaus oder ITS der Rehaklinik)
- Dokumentation von Verlegung (Normalstation, Sekundärverlegung auf die Intensivstation eines peripheren Krankenhauses, Rehaklinik)
- Gesamtmenge von kristalloiden und kolloidalen Infusionslösungen (Typ der kolloidalen Infusionslösung), parenterale und enterale Ernährung, Erythrozytenkonzentraten, Fresh Frozen Plasma, Thrombozytenkonzentraten für die Dauer des ITS-Aufenthalts, maximal jedoch für die ersten 7 Tage postoperativ auf ITS
- Parameter des kombinierten primären Endpunktes (maximal bis Tag 14 postoperativ)

- NIH Stroke Scale

Die folgenden Angaben erfolgen nur für die Dauer des ITS-Aufenthalts (maximal jedoch für die ersten 30 Tage postoperativ bei längerem IST-Aufenthalt):

- Dauer der mechanischen invasiven Beatmung
- Dauer der mechanischen nicht-invasiven Beatmung (z.B. Masken-CPAP; gilt nicht bei reiner Sauerstoffinsufflation über Katheter/ Maske)
- Dokumentation von Re-Operation, Re-Intubation, Tracheotomie, Re-ITS-Aufnahme
- Dokumentation von Begleitmedikation (z.B. Phosphodiesterasehemmer, Levosimendan, Amiodaron, Kortison, Verapamil)
- Dokumentation von Begleittherapie (mechanische kardiale Unterstützungssystemen (IABP, ECLS))
- Dokumentation von postoperativen Komplikationen (Wundinfektion, schwere Sepsis/ septischer Schock, Herz-Kreislaufstillstand, kardiopulmonale Reanimation)

5.4.9 Visite 7 (Krankenhausentlassung)

- NIH Stroke Scale
- Optional: Neurokognitive Testung 5-7 Tage postoperativ
- Dokumentation von Verlegung (privater Wohnbereich, Pflegeheim, Rehaklinik, Sekundärverlegung in ein peripheres Krankenhauses)
- Routinelabor (maximaler postoperativer Kreatininwert i.S. während des Krankenhausaufenthaltes)
- Gesamte Krankenhausverweildauer
- Parameter des kombinierten primären Endpunktes (Mortalität, Myokardinfarkt, Schlaganfall und/oder akutes Nierenversagen) maximal bis Tag 14 postoperativ bei längerem Krankenhausaufenthalt

5.5 Nachbeobachtung

Visite 8-10 (Follow up Tag 30±5, Tag 90±15 und 12±1 Monate postoperativ)

- Parameter des kombinierten Endpunkts (Mortalität, Myokardinfarkt, Schlaganfall und/oder akutes Nierenversagen) – Telefoninterview
- Optional: Neurokognitive Testung einschließlich NIH Stroke Scale 90±15 Tage postoperativ

5.6 Vorzeitiger Abbruch der Nachbeobachtung

Jeder Abbruch der Nachbeobachtung wird durch das den Patienten betreuende Zentrum mit Datum (bzw. der genauest möglichen Angabe des Zeitpunktes) sowie nach Möglichkeit unter Angabe der Umstände und Gründe dokumentiert und dem Datenmanagement des ZKS Leipzig – KKS gemeldet.

Im Rahmen der Nachbeobachtung werden 30 Tage, 90 Tage und 12 Monate postoperativ die Einzelkomponenten des primären Zielkriteriums (Mortalität, Myokardinfarkt, Schlaganfall und/oder akutes Nierenversagen) als sekundäre Ziele der klinischen Prüfung erfasst. Eine Nachbeobachtung kann nicht erfolgen, wenn ein Kontakt zu dem Patienten nicht mehr hergestellt werden kann („Lost to follow-up").

6 UNERWÜNSCHTE EREIGNISSE UND VIGILANZSYSTEM

6.1 Unerwünschte Ereignisse (AE/SAE)

6.1.1 Unerwünschte und schwerwiegende Ereignisse: allgemeine Definition

Die ICH-GCP Guideline E6, Punkte 4.11 und 5.17 und die Deklaration von Helsinki[1] stellen den Schutz des Studienteilnehmers in klinischen Prüfungen an erster Stelle. Deshalb muss auch im Rahmen klinischer Prüfungen von Behandlungen und Therapien, bei denen <u>nicht</u> die Wirkungsweise von Medikamenten und anderen Prüfprodukten untersucht wird, die Sicherheit und Unbedenklichkeit der Anwendung überprüft werden.

Unerwünschte Ereignisse (*adverse events*, AE) sind alle ungünstigen medizinischen Vorkommnisse bei einem Patienten bzw. Probanden der klinischen Forschung, bei dem eine im Prüfplan beschriebene Intervention angewandt wurde.

Dazu zählen Erkrankungen, Krankheitszeichen (auch pathologische Laborbefunde) und Symptome, die nach der Aufnahme des Patienten in die Studie (i.d.R. nach Interventionsbeginn) neu auftreten oder sich verschlechtern.

Schwerwiegende unerwünschte Ereignisse (*severe adverse events*, SAE) liegen vor (Def. nach ICH-Richtlinie E2A, Abschnitt IIB), wenn die Vorkommnisse:

- zum Tod geführt haben,
- lebensbedrohlich sind,

Hinweis: Der Begriff lebensbedrohlich bezieht sich in der Definition als schwerwiegend auf ein Ereignis, in welchem der Patient zum Zeitpunkt des Auftretens des Ereignisses in einer lebensbedrohlichen Situation war; es bezieht sich nicht auf Ereignisse, welche hypothetisch lebensbedrohlich gewesen wären, wenn das Ereignis ernsthafter gewesen wäre.

- einer stationären ärztlichen Behandlung bedürfen oder die Verlängerung eines bestehenden stationären Aufenthaltes erfordern,
- zu bleibenden Schäden führen, oder
- eine angeborene Missbildung bzw. einen Geburtsfehler darstellen.

Ein unerwünschtes Ereignis wird zusätzlich als **unerwartet** definiert, wenn es bisher in der Literatur noch nicht in der aufgetretenen Art oder in der aufgetretenen Intensität im Zusammenhang mit der Intervention beschrieben wurde.

6.1.2 Zeitraum für die Erfassung unerwünschter Ereignisse (AE)

Die Erfassung von AEs beginnt mit der studienspezifischen Intervention und endet **96 h postoperativ**.

6.1.3 Dokumentation unerwünschter Ereignisse

Folgende unerwünschte Ereignisse werden auf dem dafür vorgesehenen AE-Dokumentationsbogen dokumentiert und werden **nicht** gesondert an das ZKS Leipzig – KKS gemeldet:

(1) **Bekannte bzw. erwartete Ereignisse**:
- Thrombose, Plaqueruptur, Embolisation am Interventionsarm
- Durchblutungsstörungen am Interventionsarm
- Nervenschäden am Interventionsarm
- Schwere Hautveränderungen im Bereich der Blutdruckmanschette

(2) **Unerwartete Ereignisse**, sofern sie **möglicherweise im Kausalzusammenhang** mit der Studienintervention RIPC/Placebo stehen.

Entsprechend den obigen Ausführungen werden folgende Ereignisse aber **<u>NICHT</u>** als AE dokumentiert:

- Medizinische oder chirurgische Prozeduren, die typischerweise im perioperativen Verlauf auftreten können (z.B. jegliche Operation, Revisionseingriff, Wundinfektion, Endoskopie, Transfusion von Blutprodukten,…), aber nicht in einem vorstellbaren Zusammenhang mit der Intervention RIPC/Placebo stehen.
- Tägliche Schwankungen im Krankheitsbild, ohne dass über einen längeren Zeitpunkt eine Verschlechterung zu verzeichnen ist und ohne dass die Schwankungen in einem vorstellbaren Zusammenhang mit der Intervention RIPC/Placebo stehen. Dies betrifft auch Begleiterkrankungen.

Bei der Dokumentation unerwünschter Ereignisse werden Klassifikationen verwendet, deren genaue Definitionen im Anhang 19.7 zum Prüfplan zu finden sind.

6.1.4 Dokumentation schwerwiegender unerwünschter Ereignisse (SAE)

Unerwünschte Ereignisse nach den obigen Ausführungen, welche zusätzlich die Kriterien für **„Schwerwiegend“** erfüllen (siehe 6.1.1), werden auf dem dafür vorgesehenen SAE-Dokumentationsbogen erfasst und müssen unverzüglich gemeldet werden, wenn ein vorstellbarer Zusammenhang mit der Intervention besteht (siehe 6.1.5).

Die **Parameter des primären Endpunktes** (Tod, Myokardinfarkt, Schlaganfall und akutes Nierenversagen) sind nur dann auf dem vorgesehenen SAE-Dokumentationsbogen zu erfassen und zu melden, **wenn der Prüfer einen direkten Zusammenhang mit der Intervention RIPC / Placebo vermutet.**

6.1.5 Meldung schwerwiegender unerwünschter Ereignisse

Verantwortlichkeiten der Prüfer

Das Auftreten jedes entsprechend 6.1.1 dokumentationspflichtigen **schwerwiegenden unerwünschten Ereignisses** muss unverzüglich (innerhalb 24 Stunden) nach Bekannt werden durch den Prüfer an die unten genannte Adresse gemeldet werden:

ZKS Leipzig – KKS

Frau Madlen Dörschmann

Universität Leipzig
Zentrum für Klinische Studien Leipzig – KKS
Härtelstr. 16-18, 04107 Leipzig

Telefon: 0341- 97 16129
Fax: 0341- 97 16 278
Email: pharmacovigilance@zks.uni-leipzig.de

Verantwortlichkeiten der Studienleitung

Die Meldung des Prüfers wird auf Vollständigkeit und Plausibilität geprüft und bei Bedarf werden Rückfragen gestellt und nach verfolgt.

Das ZKS Leipzig - KKS veranlasst die Durchführung einer medizinischen Zweitbewertung hinsichtlich Kausalität und Vorhersehbarkeit des SAE durch die Studienleitung. Der SAE-CRF und diese Zweitbewertung muss innerhalb von 2 Tagen dem ZKS Leipzig – KKS (Abteilung für Arzneimittelsicherheit) per Fax an 0341- 97 16 278 geschickt werden.

Die elektronische Erfassung der SAEs erfolgt mit Hilfe eines SAE-Management-Tools der Firma eResearch Technology. Es werden nicht nur die CRF-Daten eingegeben, sondern auch das Ergebnis der medizinischen Zweitbeurteilung durch die Studienleitung hinsichtlich Kausalzusammenhang und Vorhersehbarkeit. Im Anschluss an die Dateneingabe erfolgt die Kodierung der Diagnosen und Reaktionen mit MedDRA.

Im Fall des Todes einer betroffenen Person im **Zusammenhang mit der Intervention (RIPC/ Placebo)** übermittelt der Prüfer auf Anfrage aller beteiligten Ethikkommissionen alle für die Erfüllung ihrer Aufgaben erforderlichen zusätzlichen Auskünfte.

6.2 Sicherheitsanalysen

Die Auswertung sicherheitsrelevanter Endpunkte wird im Kapitel 7 - Biometrie beschrieben.

6.3 Therapeutische Maßnahmen im Rahmen eines unerwünschten Ereignisses

Bedarf der Patient aufgrund des unerwünschten Ereignisses einer Behandlung, so muss diese nach dem derzeitigen Stand der medizinischen Forschung durchgeführt werden, um die Gesundheit des Patienten wieder herzustellen. Geeignete Geräte und Präparate zur Wiederbelebung müssen verfügbar sein, um den Patienten im Notfall schnellstmöglich behandeln zu können.

Die Art und Weise der Behandlung des AE bzw. SAE muss dokumentiert werden.

Zusätzlich ist im CRF zu dokumentieren, wie mit der Intervention (RIPC/ Placebo) umgegangen wurde:

- Intervention/ Behandlung unterbrochen
- Intervention/ Behandlung nicht unterbrochen

- unbekannt
- nicht zutreffend (z.B. wenn Intervention bereits abgeschlossen ist)

7 BIOMETRISCHE ASPEKTE DER STUDIE

7.1 Randomisationsalgorithmus

Die Randomisation erfolgt datenbankgestützt zentral im Datenmanagement des ZKS Leipzig - KKS.

Das Randomisationsverhältnis für die zwei Studienarme beträgt 1:1. Es erfolgt eine Stratifikation nach den folgenden Parametern:

- teilnehmendes Zentrum
- Risiko für perioperative Mortalität gemäß EuroSCORE (siehe Abschnitt 20.5) in zwei Gruppen:
    - Niedriges/ Mittleres Risiko EuroSCORE ≤ 5,
    - Hohes Risiko EuroSCORE ≥ 6.

Zur Randomisation wird der Minimierungsalgorithmus nach Pocock angewandt.[48]

7.2 Endpunkte der Studie

7.2.1 Primärer Endpunkt

Kombinierter klinischer Endpunkt bestehend aus

- Tod jeglicher Ursache und/oder
- Myokardinfarkt und/oder
- Schlaganfall und/oder
- Akutes Nierenversagen

in der Zeit zwischen Intervention und Krankenhausentlassung (bzw. spätestens 14 Tage postoperativ bei längerem Krankenhausaufenthalt) des Patienten.

Zur genauen Definition der Ereignisse „Myokardinfarkt“, „Schlaganfall“ und „Akutes Nierenversagen“ siehe Abschnitt 2.1.

7.2.2 Sekundäre Endpunkte

- Dauer der mechanischen invasiven Beatmung bis maximal 30 Tage postoperativ

  Anzahl der Stunden mit mechanischer Beatmung postoperativ

- Dauer der mechanischen nicht-invasiven Beatmung bis maximal 30 Tage postoperativ

  Anzahl der Stunden mit mechanischer nicht-invasiver Beatmung (z.B. mittels Masken-CPAP) postoperativ

- Gesamte Verweildauer auf der Intensivstation

  Zeit in Stunden von Ende der Operation (= Ende der Hautnaht) bis zur Entlassung von der Intensivstation einschließlich Prüfzentrum, peripheres Krakenhaus und Rehaklinik

- Gesamte Verweildauer im Krankenhaus

  Zeit von Ende der Operation (= Ende der Hautnaht) bis zur Entlassung aus dem Krankenhaus. Eine Sekundärverlegung in ein weiteres Krankenhaus zählt nicht als Entlassung

- Myokardschaden, gemessen am Verlauf des kardialen Troponin T innerhalb der ersten 48 h postoperativ
- Nierenfunktionsstörungen, gemessen am Verlauf des Kreatinin i.S. während des Krankenhausaufenthaltes
- Überlebensrate bei Krankenhausentlassung (bzw. bis maximal Tag 14 postoperativ) sowie 30 Tage, 90 Tage und 12 Monate postoperativ
- Schlaganfall (Definition siehe 2.1) bei Krankenhausentlassung (bzw. bis maximal Tag 14 postoperativ) sowie 30 Tage, 90 Tage und 12 Monate postoperativ
- Myokardinfarkt (Definition siehe 2.1) bei Krankenhausentlassung (bzw. bis maximal Tag 14 postoperativ) sowie 30 Tage, 90 Tage und 12 Monate postoperativ
- Akutes Nierenversagen (Definition siehe 2.1) bei Krankenhausentlassung (bzw. bis maximal Tag 14 postoperativ) sowie 30 Tage, 90 Tage und 12 Monate postoperativ
- Delir, gemessen mit dem CAM-ICU-Fragebogen 24, 48, 72 und 96 h postoperativ sowie gemessen am Einsatz von Medikamenten zur Delirbehandlung bis 96 h postoperativ
- Katecholaminbedarf kumulativ intraoperativ sowie innerhalb der ersten 24 h postoperativ
- Neu aufgetretenes Vorhofflimmern innerhalb der ersten 96 h postoperativ

- Einsatz von mechanischen kardialen Unterstützungssystemen als binäre Variable bis maximal 30 Tage postoperativ
- Optional: Myokardiale Dysfunktion intraoperativ
- Optional: Postoperative neurokognitive Dysfunktion

7.3 Statistische Formulierung der Studienfrage

7.3.1 Statistische Hypothese

Die primäre Fragestellung ist, ob durch ischämische Fern-Präkonditionierung (RIPC) die Rate des kombinierten klinischen Endpunkts (Tod und/oder Myokardinfakt und/oder Schlaganfall und/oder akutes Nierenversagen) gesenkt werden kann.
Da nach dem derzeitigen Kenntnisstand offen ist, ob tatsächlich ein Einfluss der ischämischen Fern *(Remote)*- Präkonditionierung vorliegt und in welche Richtung dieser mögliche Unterschied ausfällt, ist diese Fragestellung anhand eines zweiseitigen statistischen Tests zu untersuchen. Aus diesem Grund postuliert die Nullhypothese die Gleichheit und die Alternativhypothese einen Unterschied.
Die Null- und Alternativhypothesen lauten deshalb:

H_0: $p_{RIPC} = p_{Kontrolle}$

H_A: $p_{RIPC} \neq p_{Kontrolle}$

7.4 Fallzahldiskussion

7.4.1 Abschätzung der Effektgrößen

Die in der Kontrollgruppe erwarteten Raten der Einzelkomponenten des primären Endpunkts basieren im Wesentlichen auf den von der Bundesgeschäftsstelle für Qualitätssicherung GmbH in der BQS-Bundesauswertung 2008 publizierten Daten. Die Auswertung umfasst Daten zu isolierter Koronarchirurgie (n= 45.514), isolierter Aortenklappenchirurgie (n=11.160), sowie kombinierter Koronar- und Aortenklappenchirurgie (n=7.700), und macht Angaben zu folgenden Ereignissen:

- In-Hospital-Letalität
- Nierenschädigung (Injury) oder Nierenversagen (Failure oder Loss) mit den Definitionen
    - Nierenschädigung (Injury): Kreatininwert i.S. post- zu präoperativ 2- bis 3-fach erhöht oder Glomeruläre Filtrationsrate postoperativ um 50 - 75% vermindert

- Nierenversagen (Failure): postoperativ akute Nierenersatztherapie oder Kreatininwert i.S. (postoperativ) ≥ 4 mg/dl oder Kreatininwert i.S. postoperativ mehr als 3-fach erhöht oder Glomeruläre Filtrationsrate postoperativ um mehr als 75% vermindert (Failure)
- postoperativ chronische Nierenersatztherapie (Loss)

• Zerebrovaskuläres Ereignis mit einer Dauer von mehr als 24 Stunden und funktionell relevantem neurologischen Defizit bei Entlassung (Rankin ≥ 2)

Für unsere Studie gehen wir in Bezug auf den chirurgischen Eingriff von einer ähnlichen Zusammensetzung der Studienpopulation wie im BQS-Bericht aus.

Das Auftreten von nicht-fatalen Myokardinfarkten wurde in der BQS-Auswertung nicht untersucht. Relevante Studien berichten Raten an Myokardinfarkten im Bereich von 2,4%-5,1%,[29] 2,8% - 5,4%,[36] 3,9%-4,2%,[31] 6,2%-7,9%;[9] die entspricht im Median einer Rate an Myokardinfarkten von 4,7%.

Die erwarteten Ereignisraten sind in der folgenden Tabelle zusammengefasst.

	N	%
**In-Hospital-Letalität (BQS 2008)**		
Aortenklappe isoliert	11.160	3,5%
Koronarchirurgie+Aortenklappe	7.700	5,8%
Koronarchirurgie	45.514	3,1%
GESAMT		**3,5%**
**postoperative Nierenfunktionsstörung (injury / failure) (BQS 2008)**		
Aortenklappe isoliert	6.543	4,5%
Koronarchirurgie+Aortenklappe	4.241	7,3%
Koronarchirurgie	29.702	3,2%
GESAMT		**3,8%**
**zerebrovaskulären Komplikation (TIA, Schlaganfall oder Koma) (BQS 2008)**		
Aortenklappe isoliert	11.160	1,4%
Koronarchirurgie+Aortenklappe	7.700	2,0%
Koronarchirurgie	45.514	1,2%
GESAMT		**1,3%**
**Myokardinfarkt (Literatur)**		
GESAMT		**4,7%**

Summiert man die vier Komponenten des primären Endpunkts, so ergibt sich eine Gesamtrate von 13,3%. Unter Berücksichtigung der Tatsache, dass einige Myokardinfarkte bzw. Schlaganfälle einen tödlichen Ausgang hatten und daher in der Statistik doppelt zählen, gehen wir von einer Ereignisrate für den kombinierten primären Endpunkt in der Kontrollgruppe von 12% aus.

Studien im Bereich der Herzchirurgie berichten eine Risikoreduktion von 25% bis 70% mit pharmakologischen Therapien.[23,35,36,43] In unserer Studie untersuchen wir eine preisgünstige, einfache und sichere Intervention. Daher halten wir eine Risikoreduktion von 1/3 auf eine Ereignisrate von 8% für klinisch relevant.

7.4.2 Statistische Fehlergrößen

Das Signifikanzniveau wird auf α=5% festgesetzt. Es wird eine Power von 1-β=80% angestrebt.

7.4.3 Drop-outs

Für die Erfassung der vier Komponenten des primären Endpunkts ist die Zeit bis zur Krankenhausentlassung maßgeblich. Drop-outs sind nur für den Fall zu erwarten, dass Patienten in ein anderes Krankenhaus verlegt werden und von dort keine weitere Information erfolgt, oder für den sehr seltenen Fall, dass Patienten ihre Einwilligung zur Studienteilnahme zurückziehen.

Wir gehen von einer Drop-out Rate von maximal 10% aus.

7.4.4 Fallzahlkalkulation

Mit den o.g. statistischen Fehlergrößen, dem Randomisationsverhältnis von 1:1 und der erwarteten Therapieeffekte ist eine Fallzahl von 931 auswertbaren Patienten pro Arm, d.h. 1.862 für die gesamte Studie erforderlich (chi^2-Test, Berechnung mit NCSS 2007 and Power Analysis and Sample Size 2008; Kaysville, Utah, USA).
Unter Berücksichtigung der Drop-out-Rate ist eine Gesamtzahl von 2.070 zu randomisierenden Patienten erforderlich (1035 Patienten pro Arm).

7.5 Verfahren zur Datenanalyse

7.5.1 Analysepopulationen

Die konfirmatorische Analyse des primären Endpunktes und die Analysen der sekundären Endpunkte werden auf der Basis der Intention-to-treat Population (ITT) durchgeführt. Sensitivitätsanalysen erfolgen auf der Basis des Per-Protocol-Set (PPS).

Die ITT umfasst **alle** Patienten,

- die randomisiert wurden und
- deren Einwilligungserklärung vorliegt und

- bei denen mit der Intervention (RIPC / Placebo) für den geplanten herzchirurgischen Eingriff begonnen wurde einschließlich der Patienten mit Protokollverletzungen.

Zur Analysepopulation PPS gehören Patienten der ITT, bei denen **kein schwerwiegender Protokollverstoß** aufgetreten ist.

Insbesondere folgende Protokollverstöße werden als **schwerwiegend** erachtet:

- Verletzung der Ein- und Ausschlusskriterien
- keine oder falsche Studienintervention

Für sekundäre Analysen werden alle Patienten herangezogen, welche für den betrachteten Endpunkt gültige Werte besitzen. Bei Fehlwerten werden ggf. Imputationen vorgenommen, sofern dies bezüglich Anzahl der Fehlwerte und erwarteter Aussage sinnvoll erscheint. Sofern durchführbar, werden bei Fehlwerten Sensitivitätsanalysen für Best- und Worst-Case-Szenarien durchgeführt.

7.5.2 Geplante Analysemethoden

Der primäre zusammengesetzte Endpunkt ist binär. Die Bewertung des Therapieeffekts erfolgt über eine logistische Regression. Kovariaten neben der Therapie sind der EuroSCORE, der Diabetes-mellitus Status (Ja/Nein), und die Begleittherapie mit Statinen (Ja/Nein). Zentrumsunterschiede werden als Randomeffekt berücksichtigt. Die Testentscheidung erfolgt über den Wald-Test für den Koeffizienten des Therapieeffekts. Die Risikoreduktion wird als Odds-Ratio mit 95% Konfidenzintervall quantifiziert.

Die Analyse wird nach dem "Intention-to-treat"-Prinzip (ITT) durchgeführt.
Die sekundären Endpunktanalysen erfolgen deskriptiv sowie mittels Anwendung statistischer Testverfahren. Die Auswertung von Häufigkeiten erfolgt durch Chi^2- oder Fisher Exakt Test. Die Auswertung von metrischen Endpunkten erfolgt durch den Mann-Whitney-U-Test. Neben den Testergebnissen werden Effekt-Schätzer mit 95%-Konfidenzintervallen angegeben.
Zur Beschreibung der unerwünschten und schwerwiegenden unerwünschten Ereignisse werden Listen und tabellarische Zusammenfassungen erstellt.
Ein detaillierter Analyseplan wird vor Beginn der Endauswertung erstellt.

7.6 Zwischenauswertungen

Es sind keine formalen Zwischenauswertungen vorgesehen. Es wird jedoch ein statistisches Monitoring durchgeführt, um die Daten- und Durchführungsqualität zu überwachen.
In regelmäßigen Zeitabständen werden dem „Independent Data Monitoring and Safety Committee“ (IDMC) deskriptive Auswertungen zur Sicherheit sowie zur Daten- und

Durchführungsqualität der Studie vorgelegt.

7.7 Endauswertung

Die Endauswertung wird in zwei Abschnitten durchgeführt.

Die Analyse des primären Endpunkts findet statt, wenn der letzte eingeschlossene Patient bis zur Krankenhausentlassung, spätestens jedoch bis Tag 30 postoperativ nachbeobachtet wurde, und alle Nachfragen zur Dokumentation der Patienten bis Tag 30 postoperativ gelöst wurden.

Zu diesem Zeitpunkt werden auch alle sekundären Endpunkte bis Tag 30 postoperativ sowie die unerwünschten und schwerwiegenden unerwünschten Ereignisse analysiert.

Die Analyse der sekundären Endpunkte, die im Nachbeobachtungszeitraum bis zu 12 Monaten postoperativ erhoben werden, erfolgt nach Abschluss der Nachbeobachtungsphase.

8 WISSENSCHAFTLICHE BEGLEITPROJEKTE

8.1 Genomanalysen

Das Ziel des Begleitprojektes besteht in der Bewertung der Genotypisierung von spezifischen genomischen Polymorphismen in pathophysiologisch relevanten Genen.

Eine zunehmende Fülle an wissenschaftlichen Untersuchungen zeigt deutlich, dass spezifische genomische Polymorphismen (sog. *single nucleotide polymorphisms* [SNP]) von anderweitig gesunden Individuen mit einer schlechteren intensivmedizinischen und perioperativen Prognose einhergehen.[16,33,47,64] Die Identifizierung solcher Genpolymorphismen kann in Zukunft der Aufklärung der interindividuellen Variabilität der pathophysiologischen Reaktion auf schwere Krankheitszustände dienen. Das Ziel des Begleitprojektes besteht in der Bewertung der Genotypisierung von SNPs in pathophysiologisch relevanten Genen unter Kontrolle der genomischen Variabilität durch Typisierung definierter Nicht-Kandidaten SNP (Konzept der "Genomic Controls"). Es soll in dieser klinischen Prüfung eine DNA-Probenbank eingerichtet werden, um Polymorphismen inflammatorischer Mediatoren (z.B. TNF-α, Interleukinen, usw.), des Gerinnungssystems sowie des endothelialen Systems und deren prognostische Bedeutung in der postoperativen Phase nachzuweisen. Darüber hinaus soll der Therapieerfolg bzw. das Therapieversagen

der RIPC mit den SNPs korreliert werden. Diese Untersuchungen werden zu einem späteren Zeitpunkt durchgeführt.

Die entnommenen Blutproben werden Eigentum der Studienleitung.

9 ETHISCHE GRUNDLAGEN

9.1 GCP-Erklärung

Alle an dieser Studie Beteiligten (Studienleitung, Bevollmächtigte und Auftragnehmer der Studienleitung, Prüfer etc.) verpflichten sich, die klinische Prüfung nach den Vorgaben der nationalen Gesetze, den Anforderungen der ICH Guideline for Good Clinical Practice (GCP) E6 vom Juni 1996 und der CPMP/ICH/135/95 vom September 1997 durchzuführen und die Empfehlungen der Deklaration von Helsinki (Version Sommerset West 1996) zu beachten.[1,2]

10 ANTRAGSTELLUNG

Gemäß den Vorgaben der Musterberufsordnung der Ärzte §15 wird die klinische Prüfung vor Beginn den zuständigen Ethikkommissionen der teilnehmenden Prüfärzte zur Beratung vorgelegt. Hierzu wird das bereits vorhandene primäre EK-Votum des Studienleiters den Antragunterlagen beigelegt.

11 DOKUMENTATION

11.1 Patientendokumentationsbögen (CRF)

Die Erstellung des „Case Report Form“ (CRF) erfolgt durch die Studienleitung in Zusammenarbeit mit dem ZKS Leipzig - KKS. Quelldaten (source data) im Sinne der ICH-Richtlinie E6 sind alle routinemäßig erhobenen Daten und Laborberichte bzw. alle Daten in der Patientenakte. Der überwiegende Anteil der zu erfassenden Daten wird routinemäßig bei Intensivpatienten erhoben und direkt in die reguläre Patientenakte eingetragen.

Quelldaten, die direkt im CRF ohne Eintrag in der Patientenakte erfasst werden sind:

- Intervention
- Neurokognitive Testung (optional)

- Transösophageale Echokardiographie (optional)

Der CRF wird auf „non-copy carbon Paper“ gedruckt, so dass der Originalbogen zur Dateneingabe an das ZKS Leipzig - KKS geschickt werden kann. Die Kopie verbleibt im Prüfarztordner im Prüfzentrum.

Alle Einträge im CRF werden deutlich lesbar mit dunklem Kugelschreiber vorgenommen, um die Lesbarkeit von Durchschlägen oder Kopien zu gewährleisten. Korrekturen werden so durchgeführt, dass der Originaleintrag lesbar bleibt und mit Datum und Initialen der zur Korrektur berechtigten Person abgezeichnet. Sollten Daten oder Einträge fehlen, so muss dieses begründet werden. Alle relevanten CRF-Seiten werden durch den Hauptprüfer oder einen Prüfer auf Vollständigkeit und Korrektheit kontrolliert und abgezeichnet. Die Unterschrift dient der Verifizierung der CRF-Einträge.

11.1.1 Aufbau des CRF

Das CRF besteht aus einzelnen Abschnitten. Diese sind:

- Patientenregistrierung
- Screening
- Operation
- 6 / 12 Stunden postoperativ
- 24 Stunden postoperativ
- 48 Stunden postoperativ
- 72 Stunden postoperativ
- 96 Stunden postoperativ
- Verlegung von Intensivstation
- Krankenhausentlassung
- Follow up – Telefoninterview
- Re-Operation / Re-Intubation / Tracheotomie / Re-ITS-Aufnahme
- Primärer Endpunkt
- Studienende
- AE / SAE
- Intervention
- Transösophageale Echokardiographie (optional)
- Neurokognitive Testung (optional)
- Prüfplanverletzung

Die in den jeweiligen Abschnitten zu erfassenden Items werden im Rahmen der Erstellung des CRF näher spezifiziert. Ein papierbasiertes Beispiel-CRF wird den Prüfzentren als Bestandteil des Prüfarztordners ausgehändigt.

Die Prüfer sind gehalten, die entsprechenden Daten zeitnah in die CRFs zu übertragen.

11.1.2 Patientenidentifikationsliste (Prüfarztordner)

In diesem Dokument werden alle in die klinische Prüfung eingeschlossen Patienten mit vollständigem Namen, Geburtsdatum und Geschlecht erfasst und deren Patienten-Identifikationsnummer zugeordnet. Diese Liste darf das Prüfzentrum nicht verlassen und muss im Prüfarztordner abgelegt werden. Diese Liste ist notwendig, um für spätere Nachfragen durch die Dokumentationszentrale den richtigen Patienten identifizieren zu können. Die Patientenidentifizierungsliste muss nach Beendigung der klinischen Prüfung für mindestens 10 Jahre zusammen mit allen anderen Unterlagen archiviert werden (siehe 11.2) Alle patientenbezogenen Daten werden in pseudonymisierter Form erfasst. Jeder Patient ist durch eine Patientennummer, die ihm bei der Randomisierung zugewiesen wird, unverwechselbar gekennzeichnet.

Die Prüfer müssen die Patientenidentifikationsliste zur Identifikation der an der klinischen Prüfung teilnehmenden Patienten führen. Da die Daten der Patienten aus Datenschutzgründen im CRF nur pseudonymisiert eingetragen werden, wird für Notfälle, z.B. Aufarbeitung eines unerwünschten Ereignisses, diese Liste geführt, mit deren Hilfe die Prüfer in der Lage sind, den vollen Namen des Patienten herauszufinden.

11.2 Datenmanagement und Archivierung

Das Datenmanagement erfolgt unter Verwendung des Studienmanagement-Tools eResearch Network. Für die Erstellung der Studiendatenbank wird vom verantwortlichen Projektmanager am ZKS Leipzig - KKS in Zusammenarbeit mit dem Biometriker und Dokumentar ein Pflichtenheft formuliert, das dem Datenbankprogrammierer als Basis für die Erstellung der Datenbankanwendung dient.

Die CRFs werden mittels „eResearch eDatamanagement“ modelliert. Dieses Modul erzeugt in einem Oracle-DBMS (Datenbank Management System) eine Datenbank, in der die Daten aus dem CRF übertragen werden. Dies geschieht mittels einer Maske, die mit dem „eReseach Screen Modeller“ erzeugt wurde. Vor Freigabe wird die Datenbankanwendung auf Fehler getestet und validiert, der Vorgang der Validierung wird schriftlich dokumentiert.

Die auf den CRFs erfassten Daten werden über Eingabemasken in der eResearch-Datenbank erfasst. Es erfolgt eine doppelte Dateneingabe. Die eingegebenen Daten werden täglich über vorprogrammierte, automatische Checks auf Vollständigkeit, Konsistenz und Plausibilität überprüft. Rückfragen (Queries) werden zeitnah in schriftlicher Form an die Prüfzentren bzw. die verantwortlichen Monitore übermittelt.

Es erfolgt eine tägliche Komplettsicherung aller Daten. Durch den Einsatz eines hierarchischen auf Rollen basierenden Zugriffskonzeptes ist ein unberechtigter Zugriff auf die Patientendaten unmöglich. Die Anonymität der Daten im Rahmen von Auswertungen ist sichergestellt. Jede Änderung an den Daten, z.B. aufgrund der Einarbeitung von beantworteten Rückfragen, wird über einen automatischen Audittrail in der Datenbank dokumentiert.

Alle relevanten Studienunterlagen (Trial Master File), die elektronisch erfassten Daten, Originale der SAE-CRFs, der Prüfplan, eventuelle Amendements und der Abschlussbericht werden mindestens 10 Jahre nach Abschluss der klinischen Prüfung am ZKS Leipzig - KKS aufbewahrt.

In den Prüfzentren werden der Prüfarztordner, die Patientenidentifikationsliste, die unterschriebenen Einwilligungserklärungen, Kopien der SAE-CRFs und die Patientenakten mindestens 10 Jahre nach Abschluss der klinischen Prüfung aufbewahrt. Verlangen hausinterne Regelungen oder andere Gesetzgebungen (z.B. Röntgenverordnung, Strahlenschutzgesetz) eine längere Aufbewahrungsfrist, so ist diese zu berücksichtigen.

12 REFERENZBEGUTACHTUNGEN

12.1 Endpunkt Komitee

Das verblindete Endpunkt Komitee setzt sich aus Herr Professor Dr. med. Heribert Schunkert, Medizinische Klinik II – Kardiologie, Universitätsklinikum Schleswig-Holstein, Campus Lübeck und Herr Professor Dr. med. Norbert Frey, Medizinische Klinik III - Kardiologie, Universitätsklinikum Schleswig-Holstein, Campus Kiel zusammen.

Die Diagnose eines nicht-tödlichen perioperativen Myokardinfarkts nach einem herzchirurgischen Eingriff umfasst neben einem obligatorischen Troponin T Anstieg größer als 5-fach des oberen Referenzwertes u.a. eine neue pathologische Q-Welle oder einen neuen Linksschenkelblock im EKG.

Das primäre Ziel des Endpunkt Komitees ist, mit Hilfe der detaillierten Kriterien im Anhang 0 die Begutachtung der EKG-Befunde von den Patienten, die einen postoperativen Troponin T Anstieg größer als 5-fach des oberen Referenzwertes aufzeigen, verblindet zu validieren.

13 ÜBERWACHUNG DER STUDIE

13.1 Zugang zu Quelldaten

Aufgrund gesetzlicher Regelungen zur Sicherung der Datenqualität und zur Überwachung der Studiendurchführung am Zentrum sind die Prüfärzte verpflichtet, autorisierten Dritten Einsicht in die Patientenakten (Quelldaten) zu gewährleisten. Dazu zählen Monitore, Auditoren und weitere Beauftragte der Studienleitung. Diese Personen sind zur Verschwiegenheit verpflichtet.

13.2 Monitoring

Das Monitoring umfasst nach Rücksprache mit den Zentren Pre-study Visite, Initiierungsbesuch und Close-out Visite in allen teilnehmenden Prüfzentren. Die Häufigkeit der regulären Besuche hängt von der Rekrutierung im jeweiligen Prüfzentrum und von der Qualität der Studiendurchführung und der Dokumentation des Prüfzentrums ab. Es ist geplant, nach Rekrutierung von jeweils ca. 20 neuen Patienten, spätestens jedoch 3 Monate nach dem letzten regulären Besuch eine Monitoring-Visite durchzuführen. Im Rahmen dieser regulären Monitoring-Besuche werden alle studienrelevanten Dokumente kontrolliert und ggf. aktualisiert. Dies umfasst insbesondere die Einhaltung der Vorgaben des Prüfplans und der GCP. Bei allen Patienten erfolgt eine Kontrolle der Einwilligungserklärungen.

Für ein umfangreicheres Monitoring werden 50% der Patienten zufällig ausgewählt. Bei diesen Patienten werden Ein- / Ausschlusskriterien, Durchführung der Intervention und die Erfassung der Endpunkte kontrolliert.

Der Umfang des Quelldatenvergleichs wird im Monitoring Manual festgelegt. Zur Durchführung des Monitorings ermöglichen die Prüfer den Zutritt zu den Räumlichkeiten der Studiendurchführung, inkl. Zugriff auf die Akten aller Studienteilnehmer zur Gewährleistung eines vollständigen Quelldatenvergleichs. Die exakte Planung und Durchführung des Monitorings basiert auf den dafür verfügbaren SOPs des ZKS Leipzig – KKS und wird in einem noch zu erstellenden Monitoringmanual genauer beschrieben.

13.3 Audits

Der Förderer und die Studienleitung haben das Recht, in den Prüfzentren Audits durchzuführen. Die Hauptprüfer erlauben daher eine generelle Überprüfung Ihres Zentrums.

13.4 Unabhängige Überwachung der Studie

Im Rahmen dieser Studie beurteilt ein „Independent Data Monitoring and Safety Committee“ (IDMC) bestehend aus unterschiedlichen Experten regelmäßig die Sicherheit der Studienintervention sowie die Integrität und die Validität der erhobenen Daten und die Durchführung der klinischen Prüfung, um die Sicherheitsinteressen der Teilnehmer zu wahren.
Das IDMC hat darüber hinaus die Aufgabe, der Studienleitung Empfehlungen hinsichtlich der weiteren Durchführung (z.B. Abbruch oder Modifizierung) der Studie anhand der vorgelegten Analyse zu geben.
Die Tätigkeit des IDMC wird in einem noch zu erstellenden IDMC-Charter genauer beschrieben.

14 DATENSCHUTZ UND SCHWEIGEPFLICHT

Die klinische Prüfung dient einem rein wissenschaftlichen Ziel. Zur Erreichung des Zieles der klinischen Prüfung ist es erforderlich, medizinische Daten einzelner Patienten zu erheben und zu verarbeiten. Die Datenerhebung erfolgt in den an der klinischen Prüfung beteiligten Zentren (siehe Anhang). Eine namensmäßige Identifizierung bestimmter Patienten durch die Dokumentationszentrale ist zu keinem Zeitpunkt der klinischen Prüfung erforderlich.

Die Übertragung der patientenbezogenen medizinischen Daten von den Prüfzentren zur Dokumentationszentrale erfolgt unter Verwendung eines Pseudonyms; es werden keine Merkmale übertragen, welche eine unmittelbare Identifizierung bestimmter Patienten in der Dokumentationszentrale erlauben. Zur Beantwortung von Rückfragen im Rahmen der laufenden Überwachung der Dokumentationsqualität ist im Prüfzentrum jedoch eine Zuordnung des Pseudonyms zu einem bestimmten Patienten nötig. Dies erfolgt mit Hilfe einer Patientenidentifikationsliste, die stets im Prüfzentrum verbleibt.

Im Rahmen der Datenverarbeitung in der Studienzentrale des ZKS Leipzig – KKS wird mit Hilfe eines Sicherheitskonzepts u.a. der Schutz vor unbefugtem Zugriff und der Schutz vor Datenverlust sichergestellt und dafür Sorge getragen, dass die Bestimmungen des Datenschutzgesetzes eingehalten werden. Die Studiendaten sind vor fremden Zugriff

geschützt und nur Mitarbeiter der Studie dürfen auf diese zugreifen. Diese Mitarbeiter sind zur Verschwiegenheit verpflichtet.

Im Falle eines Widerrufs der Einwilligung durch den Patienten wird geprüft, inwieweit die gespeicherten Daten noch erforderlich sind. Nicht mehr benötigte Daten werden unverzüglich gelöscht. Die erhobenen personenbezogenen Daten werden nach 10 Jahren gelöscht, soweit nicht gesetzliche, satzungsmäßige oder vertragliche Aufbewahrungsfristen entgegenstehen.

Die im Rahmen des Ablaufs der klinischen Prüfung entnommenen DNA-Proben werden mit einer studienweit eindeutigen DNA-ID versehen und an die zentrale Biobank popgen verschickt. Der Biobank werden weder Identifizierungsmerkmale noch Pseudonyme übermittelt. Eine unmittelbare Identifizierung bestimmter Patienten ist der zentralen Biobank dadurch nicht möglich. Die DNA-IDs und die Ergebnisse der laboranalytischen Untersuchungen der DNA-Proben werden zusammen mit dem Pseudonym nach Abschluss der klinischen Prüfung in der Dokumentationszentrale zusammengeführt.

Erklärung zum Datenschutz

Bei Dateneingabe, -verarbeitung und -auswertung, die im Zentrum für Klinische Studien Leipzig – KKS, Universität Leipzig, Härtelstr. 16-18, 04107 Leipzig erfolgt, werden die Bestimmungen des Datenschutzgesetzes eingehalten. Zugriff auf alle Studiendaten haben nur Mitarbeiter der Studie. Diese Personen sind zur Verschwiegenheit verpflichtet. Die Daten sind vor fremden Zugriff geschützt.

15 ADMINISTRATIVE REGELUNGEN

15.1 Studiendurchführung nach Prüfplan

Die hier dargestellte Studie wird entsprechend den Anforderungen von ICH-GCP und den geltenden gesetzlichen Bestimmungen geplant, durchgeführt und ausgewertet.

Protokollverletzungen sind sämtliche Abweichungen von den Anweisungen und Abläufen, die in diesem Prüfplan beschrieben werden. Dazu gehören:

- fehlende Untersuchungen bzw. Durchführung dieser zum falschen Zeitpunkt
- fehlende Compliance
- Einnahme unerlaubter Begleitmedikamente
- Verletzung der Ein- oder Ausschlusskriterien

- falsche Studienintervention

Nachdem ein Studienteilnehmer in die Studie aufgenommen wurde, liegt es in der Verantwortung des Prüfers Protokollverletzungen zu vermeiden, um den Patienten in der Studie zu halten.

Schwerwiegende Protokollverletzungen werden unverzüglich an die Studienleitung gemeldet. Die Einschätzung von Protokollverletzungen erfolgt im Monitoringmanual. Alle Protokollverletzungen werden dokumentiert und vor der statistischen Auswertung der Studie mit dem verantwortlichen Biometriker diskutiert.

Der Prüfer muss sicherstellen, dass alle erhobenen Daten entsprechend dem Prüfplan dokumentiert werden. Kleinere Abweichungen sind im Arbeitsalltag sicherlich nicht zu vermeiden, müssen allerdings mit einer Begründung dokumentiert werden.

15.2 Nachträgliche Prüfplanänderungen

Änderungen der von den Ethik-Kommissionen zustimmend bewerteten klinischen Prüfung, die geeignet sind,

- sich auf die Sicherheit der betroffenen Personen auszuwirken (z.B. Änderung der Studieninterventionen),
- zusätzliche Datenerhebungen oder Auswertungen, die eine Änderung der Patientenaufklärung und/oder -einwilligung erfordern.
- die Auslegung der wissenschaftlichen Dokumente, auf die die klinische Prüfung gestützt wird, oder die wissenschaftliche Aussagekraft der Studienergebnisse zu beeinflussen,
- die Art der Leitung oder Durchführung der klinischen Prüfung wesentlich zu verändern,

dürfen nur vorgenommen werden, wenn diese Änderungen von der Ethik-Kommission zustimmend bewertet wurden.

Kleinere, d. h. nicht genehmigungspflichtige Prüfplanänderungen, die nicht unter die oben aufgeführten Punkte fallen, werden durch die Studienleitkommission per „*Note To File*“ schriftlich festgehalten und an die Prüfzentren verteilt.

15.3 Finanzierung und Versicherungen

Diese klinische Prüfung wird unter dem Förderkennzeichen ME 3559/1-1 von der Deutschen Forschungsgemeinschaft (DFG) gefördert.

Im Rahmen der klinischen Prüfung sind die Patienten gemäß den gesetzlichen Vorgaben versichert. Die Höchstersatzleistung für die einzelne versicherte Person beträgt 500.000,- Euro für die gesamte Dauer der klinischen Prüfung.

Die Versicherung erfolgt über die Atrialis GmbH, Obereckstr. 49A, 79539 Lörrach. Als Versicherer fungiert die CHUBB INSURANCE COMPANY OF EUROPE SE, Direktion für Deutschland, Grafenberger Allee 295, 40237 Düsseldorf. Der zugehörige Versicherungsschein trägt die Nummer: 33224473.

Eine Kopie der Police und Allgemeine Versicherungsbedingungen befinden sich im Prüfarztordner. Eine Kopie der Allgemeine Versicherungsbedingungen kann auf Anfrage den Patienten ausgehändigt werden.

15.4 Publikationsvereinbarungen und Registrierung

Es wird angestrebt, die Ergebnisse dieser klinischen Prüfung in einer renommierten internationalen medizinischen Fachzeitschrift zu veröffentlichen. In diesem Zusammenhang wurde diese klinische Prüfung bei http://www.controlled-trials.com unter der Registrierungsnummer ISRCTN01067703 registriert.

Neben den Studienleitern und dem verantwortlichen Biometriker soll jedes Prüfzentrum mit zwei selbst genannten Autoren in der Reihenfolge entsprechend der Anzahl der eingeschlossenen Patienten pro Zentrum berücksichtigt werden. Die Namen aller der in den Prüfzentren an der klinischen Prüfung beteiligten Mitarbeiter sollen zusätzlich unter dem Stichwort "*RIPHeart Investigators*" gelistet werden.

16 LITERATURVERZEICHNIS

[1] Declaration of Helsinki: Guiding Physicians in Biomedical Research Involving Human Subjects. Adopted by the 18th World Medical Assembly, Helsinki (Finland), June 1964. Last amendment by the 48th General Assembly, Somerset West (Rep. of South Africa) 1996.

[2] International Conference on Harmonisation of Technical Requirements for the Registration of Pharmaceutical Products for Human Use: ICH Harmonized Tripartite Guideline, "Guideline for Good Clinical Practice". Recommended for Adoption at Step 4 of the ICH Process on 1 May 1996. www.ifpma.org/ich5e.html.

[3] Ali ZA, Callaghan CJ, Lim E, Ali AA, Nouraei SA, Akthar AM, Boyle JR, Varty K, Kharbanda RK, Dutka DP, Gaunt ME: Remote ischemic preconditioning reduces myocardial and renal injury after elective abdominal aortic aneurysm repair: a randomized controlled trial. Circulation 2007; 116: I98-105

[4] Arrowsmith JE, Grocott HP, Reves JG, Newman MF: Central nervous system complications of cardiac surgery. Br J Anaesth 2000; 84: 378-93

[5] Bein B, Renner J, Caliebe D, Scholz J, Paris A, Fraund S, Zaehle W, Tonner PH: Sevoflurane but not propofol preserves myocardial function during minimally invasive direct coronary artery bypass surgery. Anesth Analg 2005; 100: 610-6

[6] Biancari F, Mosorin M, Rasinaho E, Lahtinen J, Heikkinen J, Niemela E, Anttila V, Lepojarvi M, Juvonen T: Postoperative stroke after off-pump versus on-pump coronary artery bypass surgery. J Thorac Cardiovasc Surg 2007; 133: 169-73

[7] Boodhwani M, Rubens FD, Wozny D, Rodriguez R, Alsefaou A, Hendry PJ, Nathan HJ: Predictors of early neurocognitive deficits in low-risk patients undergoing on-pump coronary artery bypass surgery. Circulation 2006; 114: I461-6

[8] Cheung MM, Kharbanda RK, Konstantinov IE, Shimizu M, Frndova H, Li J, Holtby HM, Cox PN, Smallhorn JF, Van Arsdell GS, Redington AN: Randomized controlled trial of the effects of remote ischemic preconditioning on children undergoing cardiac surgery: first clinical application in humans. J Am Coll Cardiol 2006; 47: 2277-82

[9] Collard CD, Body SC, Shernan SK, Wang S, Mangano DT: Preoperative statin therapy is associated with reduced cardiac mortality after coronary artery bypass graft surgery. J Thorac Cardiovasc Surg 2006; 132: 392-400

[10] Dave KR, Saul I, Prado R, Busto R, Perez-Pinzon MA: Remote organ ischemic preconditioning protect brain from ischemic damage following asphyxial cardiac arrest. Neurosci Lett 2006; 404: 170-5

[11] Doddakula K, Al-Sarraf N, Gately K, Hughes A, Tolan M, Young V, McGovern E: Predictors of acute renal failure requiring renal replacement therapy post cardiac surgery in patients with preoperatively normal renal function. Interact Cardiovasc Thorac Surg 2007; 6: 314-8

[12] Eagle KA, Guyton RA, Davidoff R, Edwards FH, Ewy GA, Gardner TJ, Hart JC, Herrmann HC, Hillis LD, Hutter AM, Jr., Lytle BW, Marlow RA, Nugent WC, Orszulak TA: ACC/AHA 2004 guideline update for coronary artery bypass graft surgery: a report of the American College of Cardiology/American Heart Association Task Force on Practice Guidelines. Circulation 2004; 110: e340-437

[13] Ely EW, Shintani A, Truman B, Speroff T, Gordon SM, Harrell FE, Jr., Inouye SK, Bernard GR, Dittus RS: Delirium as a predictor of mortality in mechanically ventilated patients in the intensive care unit. Jama 2004; 291: 1753-62

[14] Fellahi JL, Hedoire F, Le Manach Y, Monier E, Guillou L, Riou B: Determination of the threshold of cardiac troponin I associated with an adverse postoperative outcome after cardiac surgery: a comparative study between coronary artery bypass graft, valve surgery, and combined cardiac surgery. Crit Care 2007; 11: R106

[15] Gogbashian A, Sedrakyan A, Treasure T: EuroSCORE: a systematic review of international performance. Eur J Cardiothorac Surg 2004; 25: 695-700

[16] Haase-Fielitz A, Haase M, Bellomo R, Dragun D: Genetic polymorphisms in sepsis- and cardiopulmonary bypass-associated acute kidney injury. Contrib Nephrol 2007; 156: 75-91

[17] Hausenloy DJ, Mwamure PK, Venugopal V, Harris J, Barnard M, Grundy E, Ashley E, Vichare S, Di Salvo C, Kolvekar S, Hayward M, Keogh B, MacAllister RJ, Yellon DM: Effect of remote ischaemic preconditioning on myocardial injury in patients undergoing coronary artery bypass graft surgery: a randomised controlled trial. Lancet 2007; 370: 575-9

[18] Hausenloy DJ, Yellon DM: Remote ischaemic preconditioning: underlying mechanisms and clinical application. Cardiovasc Res 2008

[19] Heidbreder M, Naumann A, Tempel K, Dominiak P, Dendorfer A: Remote vs.

ischaemic preconditioning: the differential role of mitogen-activated protein kinase pathways. Cardiovasc Res 2008; 78: 108-15

[20] Hoole SP, Heck PM, Sharples L, Khan SN, Duehmke R, Densem CG, Clarke SC, Shapiro LM, Schofield PM, O'Sullivan M, Dutka DP: Cardiac Remote Ischemic Preconditioning in Coronary Stenting (CRISP Stent) Study: a prospective, randomized control trial. Circulation 2009; 119: 820-7

[21] Iglesias-Garriz I, Corral F, Rodriguez MA, Garrote C, Montes M, Sevillano E: Pre-infarction angina elicits greater myocardial viability on reperfusion after myocardial infarction: a dobutamine stress echocardiographic study. J Am Coll Cardiol 2001; 37: 1846-50

[22] Kaireviciute D, Aidietis A, Lip GY: Atrial fibrillation following cardiac surgery: clinical features and preventative strategies. Eur Heart J 2009; 30: 410-25

[23] Karkouti K, Wijeysundera DN, Beattie WS: Risk associated with preoperative anemia in cardiac surgery: a multicenter cohort study. Circulation 2008; 117: 478-84

[24] Kharbanda RK, Li J, Konstantinov IE, Cheung MM, White PA, Frndova H, Stokoe J, Cox P, Vogel M, Van Arsdell G, MacAllister R, Redington AN: Remote ischaemic preconditioning protects against cardiopulmonary bypass-induced tissue injury: a preclinical study. Heart 2006; 92: 1506-11

[25] Kharbanda RK, Mortensen UM, White PA, Kristiansen SB, Schmidt MR, Hoschtitzky JA, Vogel M, Sorensen K, Redington AN, MacAllister R: Transient limb ischemia induces remote ischemic preconditioning in vivo. Circulation 2002; 106: 2881-3

[26] Kloner RA, Jennings RB: Consequences of brief ischemia: stunning, preconditioning, and their clinical implications: part 1. Circulation 2001; 104: 2981-9

[27] Kloner RA, Jennings RB: Consequences of brief ischemia: stunning, preconditioning, and their clinical implications: part 2. Circulation 2001; 104: 3158-67

[28] Klugkist M, Sedemund-Adib B, Schmidtke C, Schmucker P, Sievers HH, Huppe M: [Confusion Assessment Method for the Intensive Care Unit (CAM-ICU): diagnosis of postoperative delirium in cardiac surgery]. Anaesthesist 2008; 57: 464-74

[29] Landoni G, Biondi-Zoccai GG, Zangrillo A, Bignami E, D'Avolio S, Marchetti C, Calabro MG, Fochi O, Guarracino F, Tritapepe L, De Hert S, Torri G: Desflurane and sevoflurane in cardiac surgery: a meta-analysis of randomized clinical trials. J Cardiothorac

Vasc Anesth 2007; 21: 502-11

[30] Lassnigg A, Schmid ER, Hiesmayr M, Falk C, Druml W, Bauer P, Schmidlin D: Impact of minimal increases in serum creatinine on outcome in patients after cardiothoracic surgery: do we have to revise current definitions of acute renal failure? Crit Care Med 2008; 36: 1129-37

[31] Liakopoulos OJ, Choi YH, Haldenwang PL, Strauch J, Wittwer T, Dorge H, Stamm C, Wassmer G, Wahlers T: Impact of preoperative statin therapy on adverse postoperative outcomes in patients undergoing cardiac surgery: a meta-analysis of over 30,000 patients. Eur Heart J 2008; 29: 1548-59

[32] Linden MD, Whittaker P, Frelinger AL, 3rd, Barnard MR, Michelson AD, Przyklenk K: Preconditioning ischemia attenuates molecular indices of platelet activation-aggregation. J Thromb Haemost 2006; 4: 2670-7

[33] Liu KY, Muehlschlegel JD, Perry TE, Fox AA, Collard CD, Body SC, Shernan SK: Common genetic variants on chromosome 9p21 predict perioperative myocardial injury after coronary artery bypass graft surgery. J Thorac Cardiovasc Surg; 139: 483-8, 488 e1-2

[34] Lu JC, Shaw M, Grayson AD, Poullis M, Pullan M, Fabri BM: Do beating heart techniques applied to combined valve and graft operations reduce myocardial damage? Interact Cardiovasc Thorac Surg 2008; 7: 111-5

[35] Magee MJ, Alexander JH, Hafley G, Ferguson TB, Jr., Gibson CM, Harrington RA, Peterson ED, Califf RM, Kouchoukos NT, Herbert MA, Mack MJ: Coronary artery bypass graft failure after on-pump and off-pump coronary artery bypass: findings from PREVENT IV. Ann Thorac Surg 2008; 85: 494-9

[36] Mangano DT: Aspirin and mortality from coronary bypass surgery. N Engl J Med 2002; 347: 1309-17

[37] Murkin JM, Newman SP, Stump DA, Blumenthal JA: Statement of consensus on assessment of neurobehavioral outcomes after cardiac surgery. Ann Thorac Surg 1995; 59: 1289-95

[38] Nashef SA, Roques F, Michel P, Gauducheau E, Lemeshow S, Salamon R: European system for cardiac operative risk evaluation (EuroSCORE). Eur J Cardiothorac Surg 1999; 16: 9-13

[39] Newman MF, Kirchner JL, Phillips-Bute B, Gaver V, Grocott H, Jones RH, Mark DB, Reves JG, Blumenthal JA: Longitudinal assessment of neurocognitive function after coronary-artery bypass surgery. N Engl J Med 2001; 344: 395-402

[40] Newman MF, Mathew JP, Grocott HP, Mackensen GB, Monk T, Welsh-Bohmer KA, Blumenthal JA, Laskowitz DT, Mark DB: Central nervous system injury associated with cardiac surgery. Lancet 2006; 368: 694-703

[41] Newman MF, Wolman R, Kanchuger M, Marschall K, Mora-Mangano C, Roach G, Smith LR, Aggarwal A, Nussmeier N, Herskowitz A, Mangano DT: Multicenter preoperative stroke risk index for patients undergoing coronary artery bypass graft surgery. Multicenter Study of Perioperative Ischemia (McSPI) Research Group. Circulation 1996; 94: II74-80

[42] Ouimet S, Kavanagh BP, Gottfried SB, Skrobik Y: Incidence, risk factors and consequences of ICU delirium. Intensive Care Med 2007; 33: 66-73

[43] Pan W, Pintar T, Anton J, Lee VV, Vaughn WK, Collard CD: Statins are associated with a reduced incidence of perioperative mortality after coronary artery bypass graft surgery. Circulation 2004; 110: II45-9

[44] Pandharipande P, Cotton BA, Shintani A, Thompson J, Costabile S, Truman Pun B, Dittus R, Ely EW: Motoric subtypes of delirium in mechanically ventilated surgical and trauma intensive care unit patients. Intensive Care Med 2007; 33: 1726-31

[45] Paparella D, Mickleborough LL, Carson S, Ivanov J: Mild to moderate mitral regurgitation in patients undergoing coronary bypass grafting: effects on operative mortality and long-term significance. Ann Thorac Surg 2003; 76: 1094-100

[46] Pegg TJ, Selvanayagam JB, Karamitsos TD, Arnold RJ, Francis JM, Neubauer S, Taggart DP: Effects of off-pump versus on-pump coronary artery bypass grafting on early and late right ventricular function. Circulation 2008; 117: 2202-10

[47] Perry TE, Muehlschlegel JD, Liu KY, Fox AA, Collard CD, Body SC, Shernan SK: C-Reactive protein gene variants are associated with postoperative C-reactive protein levels after coronary artery bypass surgery. BMC Med Genet 2009; 10: 38

[48] Pocock SJ: Methods of Randomization, Clinical trials: a practical approach. Chichester, John Wiley & Sons 1983, pp 66-89

[49] Przyklenk K, Bauer B, Ovize M, Kloner RA, Whittaker P: Regional ischemic

'preconditioning' protects remote virgin myocardium from subsequent sustained coronary occlusion. Circulation 1993; 87: 893-9

[50] Rahman IA, Mascaro JG, Steeds RP, Frenneaux MP, Nightingale P, Gosling P, Townsend P, Townend JN, Green D, Bonser RS: Remote ischemic preconditioning in human coronary artery bypass surgery: from promise to disappointment? Circulation 2010; 122: S53-9

[51] Ren C, Gao X, Steinberg GK, Zhao H: Limb remote-preconditioning protects against focal ischemia in rats and contradicts the dogma of therapeutic time windows for preconditioning. Neuroscience 2008; 151: 1099-103

[52] Rosner MH, Okusa MD: Acute kidney injury associated with cardiac surgery. Clin J Am Soc Nephrol 2006; 1: 19-32

[53] Rubens FD, Boodhwani M, Mesana T, Wozny D, Wells G, Nathan HJ: The cardiotomy trial: a randomized, double-blind study to assess the effect of processing of shed blood during cardiopulmonary bypass on transfusion and neurocognitive function. Circulation 2007; 116: I89-97

[54] Schmidt MR, Smerup M, Konstantinov IE, Shimizu M, Li J, Cheung M, White PA, Kristiansen SB, Sorensen K, Dzavik V, Redington AN, Kharbanda RK: Intermittent peripheral tissue ischemia during coronary ischemia reduces myocardial infarction through a KATP-dependent mechanism: first demonstration of remote ischemic perconditioning. Am J Physiol Heart Circ Physiol 2007; 292: H1883-90

[55] Sellke FW, DiMaio JM, Caplan LR, Ferguson TB, Gardner TJ, Hiratzka LF, Isselbacher EM, Lytle BW, Mack MJ, Murkin JM, Robbins RC: Comparing on-pump and off-pump coronary artery bypass grafting: numerous studies but few conclusions: a scientific statement from the American Heart Association council on cardiovascular surgery and anesthesia in collaboration with the interdisciplinary working group on quality of care and outcomes research. Circulation 2005; 111: 2858-64

[56] Selvanayagam JB, Petersen SE, Francis JM, Robson MD, Kardos A, Neubauer S, Taggart DP: Effects of off-pump versus on-pump coronary surgery on reversible and irreversible myocardial injury: a randomized trial using cardiovascular magnetic resonance imaging and biochemical markers. Circulation 2004; 109: 345-50

[57] Solomon SD, Anavekar NS, Greaves S, Rouleau JL, Hennekens C, Pfeffer MA:

Angina pectoris prior to myocardial infarction protects against subsequent left ventricular remodeling. J Am Coll Cardiol 2004; 43: 1511-4

[58] Stallwood MI, Grayson AD, Mills K, Scawn ND: Acute renal failure in coronary artery bypass surgery: independent effect of cardiopulmonary bypass. Ann Thorac Surg 2004; 77: 968-72

[59] Symons JA, Myles PS: Myocardial protection with volatile anaesthetic agents during coronary artery bypass surgery: a meta-analysis. Br J Anaesth 2006; 97: 127-36

[60] Tomai F, Crea F, Chiariello L, Gioffre PA: Ischemic preconditioning in humans: models, mediators, and clinical relevance. Circulation 1999; 100: 559-63

[61] Wolfrum S, Schneider K, Heidbreder M, Nienstedt J, Dominiak P, Dendorfer A: Remote preconditioning protects the heart by activating myocardial PKCepsilon-isoform. Cardiovasc Res 2002; 55: 583-9

[62] Yehia M, Collins JF, Beca J: Acute renal failure in patients with pre-existing renal dysfunction following coronary artery bypass grafting. Nephrology (Carlton) 2005; 10: 541-3

[63] Zhao HG, Sun XC, Xian XH, Li WB, Zhang M, Li QJ: The role of nitric oxide in the neuroprotection of limb ischemic preconditioning in rats. Neurochem Res 2007; 32: 1919-26

[64] Ziegeler S, Kleinschmidt S, Collard CD: [Gene polymorphism in intensive care patients. Is the course of disease predetermined?]. Anaesthesist 2004; 53: 213-27

17 UNTERSCHRIFTEN ZUM PRÜFPLAN

Bestätigung des Prüfplans

Der Prüfplan wird hiermit in seiner endgültigen Fassung bestätigt:

PD Dr. P. Meybohm<br>Studienleitung	Datum	Unterschrift
Prof. Dr. B. Bein<br>Studienleitung	Datum	Unterschrift
Prof. Dr. J. Cremer<br>Studienleitung	Datum	Unterschrift
Prof. Dr. Dr. K. Zacharowski<br>Studienleitung	Datum	Unterschrift
Dr. D. Hasenclever<br>Biometriker	Datum	Unterschrift

18 ANERKENNUNG DES PRÜFPLANS

(vom Hauptprüfer des jeweiligen Zentrums vor Eintritt des Zentrums in die Studie bzw. vor Behandlung von Studienteilnehmern zu unterzeichnen)

Hiermit bestätige ich, dass ich den vorliegenden Prüfplan gelesen und verstanden habe und in allen Teilen anerkenne. Ich verpflichte mich, dafür zu sorgen, dass die von meinem Zentrum in die Studie eingebrachten Patienten nach den Festlegungen dieses Prüfplans behandelt, beobachtet und dokumentiert werden. Ich verpflichte mich, dafür Sorge zu tragen, dass alle an der klinischen Prüfung beteiligten Personen über den Inhalt des Prüfplans informiert sind.

Datum: ____________________________

Unterschrift Hauptprüfer (Prüfzentrum): ____________________________

Adresse Prüfzentrum (Stempel): ____________________________

____

____

19 ANHANG

19.1 Confusion Assessment Method for the Intensive Care Unit (CAM-ICU)

Bei tiefer Sedierung (Richmond Agitation und Sedation Scale (RASS) = -4 und -5) ist die CAM-ICU nicht durchführbar.

<table>
<tr><td colspan="2">Merkmal 1: Psychische Veränderungen < 24h
Positiv, wenn Antwort JA entweder in Frage 1A oder 1B</td><td>Positiv</td><td>Negativ</td></tr>
<tr><td colspan="2">1A) Akute Veränderung: Ist der mentale Zustand des Pat. anders als präoperativ?
ODER
1B) Fluktuierender Verlauf: Zeigt der Pat. innerhalb der letzten 24 h fluktuierende Veränderungen in seinem Bewusstseinszustand, z.B. anhand der RASS oder vorausgegangener Delir-Einstufung?
(z.B. positiv, wenn Pat. zwar im Moment einen RASS von 0 hat, aber in den letzten 24 h RASS von –1, –3 oder +2 hatte)</td><td></td><td></td></tr>
<tr><td colspan="2">Merkmal 2: Aufmerksamkeitsstörung
Positiv, wenn ≥ 3 Fehler</td><td>Positiv</td><td>Negativ</td></tr>
<tr><td colspan="2">„Ich lese Ihnen jetzt hintereinander einige Buchstaben vor. Wenn Sie ein „A“ hören, drücken Sie meine Hand.“ Dann die folgenden Buchstaben in normaler Lautstärke vorlesen:
A-N-A-N-A-S-B-A-U-M (alternativ z.B. A-B-R-A-K-A-D-A-B-R)
Als Fehler zählen:
1) Pat. drückt bei „A“ nicht die Hand.
2) Pat. drückt bei einem anderen Buchstaben als „A“ die Hand.</td><td colspan="2">Fehler:</td></tr>
<tr><td colspan="2">Merkmal 3: Unorganisiertes Denken
Positiv, wenn in Aufgaben 1-5 insgesamt ≥ 2 Fehler.</td><td>Positiv</td><td>Negativ</td></tr>
<tr><td colspan="2">Entweder Set 1 oder Set 2 verwenden, ggf. tageweise abwechseln</td><td colspan="2">Fehler:</td></tr>
<tr><td>Set 1
1. Schwimmt ein Stein auf dem Wasser?
2. Gibt es Fische im Meer?
3. Wiegt ein Kilo mehr als 2 Kilo?
4. Kann man mit einem Hammer Nägel in die Wand schlagen?</td><td>Set 2
1. Schwimmt eine Ente auf dem Wasser?
2. Leben Elefanten im Meer?
3. Wiegen zwei Kilo mehr als ein Kilo?
4. Kann man mit einem Hammer Holz sägen?</td><td colspan="2" rowspan="2"></td></tr>
<tr><td colspan="2">5. Aufforderung
Sagen Sie dem Pat.: „Halten Sie so viele Finger hoch“, (Untersucher hält 2 Finger hoch), „jetzt machen Sie dasselbe mit der anderen Hand“ (ohne dass erneut die Anzahl der gewünschten Finger genannt wird). Falls Pat. nicht beide Arme bewegen kann, wird für den 2. Teil der Frage die Anleitung „fügen Sie einen Finger hinzu“ gegeben.</td></tr>
<tr><td colspan="2">Merkmal 4: Bewusstseinsstörung (zum Untersuchungszeitpunkt)
Positiv, wenn der aktuelle RASS zum Untersuchungszeitpunkt von Null verschieden ist.
(z.B. positiv, wenn Pat. häufige, ungerichtete Bewegungen ausführt und gegen den Ventilator kämpft; d.h. RASS= +2)</td><td>Positiv</td><td>Negativ</td></tr>
<tr><td colspan="2"></td><td></td><td></td></tr>
<tr><td colspan="2">Gesamt CAM-ICU
Merkmal 1 positiv
UND
Merkmal 2 positiv
UND
entweder Merkmal 3 ODER Merkmal 4 positiv</td><td>Positiv</td><td>Negativ</td></tr>
</table>

Copyright © 2002, E. Wesley Ely, MD, MPH und Vanderbilt University, all rights reserved

19.2 EKG-Veränderungen

EKG Veränderungen bei akuter Myokardischämie:

(nur bei Fehlen von Linksschenkelblock)

ST-Hebung	neue ST-Hebung am J-Punkt in 2 benachbarten Ableitungen: ≥0,2 mV bei Männern und ≥0,15 mV bei Frauen in den Ableitungen V_{2-3} und/oder ≥0,1 mV in den übrigen Ableitungen
ST-Senkung	neue horizontale ST-Senkung ≥0,05 mV in 2 benachbarten Ableitungen; und/oder negatives T ≥0,1 mV in 2 benachbarten Ableitungen mit prominenter R-Zacke oder R/S-Verhältnis >1

EKG Veränderungen bei zurückliegender Myokardischämie:

- jede Q-Zacke in V_2 und V_3 ≥ 0,02 s oder QS-Komplex (=R-Verlust) in V_2 und V_3
- Q-Zacke ≥ 0,03 s breit und ≥ 0,1 mV tief oder QS-Komplex in 2 benachbarten Ableitungen innerhalb der Gruppen i) I, aVL, V_6, ii) V_4-V_6 oder iii) II, III und aVF.
- R-Zacke ≥ 0,04 s in V_1-V_2 und R/S ≥ 1 mit konkordant positiver T-Welle

Referenz: Thygesen K, et al. „Joint Task Force of the European Society of Cardiology/ American College of Cardiology/ American Heart Association for the Redefinition of Myocardial Infarction" Eur Heart Journal 2007; 28:2525-2538

19.3 Abkürzungen

ACB	Aortokoronarer Bypass
AE	unerwünschtes Ereignis (*adverse event*)
BQS	Bundesgeschäftsstelle Qualitätssicherung
CAM-ICU	Confusion Assessment Method for the Intensive Care Unit
CPAP	Continuous positive airway pressure
CRF	Case Report Form
ECLS	Extrakorporale Kreislaufunterstützung
EF	Ejektionsfraktion
EK	Ethikkommission
EuroSCORE	European System for Cardiac Operative Risk Evaluation
GCP	Good Clinical Practice
HLM	Herz-Lungen-Maschine
IABP	Intraaortale Ballonpumpe
ICH	International Conference on Harmonisation
IDMC	Independent Data Monitoring and Safety Committee
IMC	Intermediate Care Unit
INR	International Normalized Ratio
ITS	Intensivstation
ITT	Intention-to-treat
KH	Krankenhaus
NIHSS	National Institute of Health Stroke Scale
POCD	Postoperative neurokognitive Dysfunktion
PPS	Per-Protocol-Set
PTT	Partielle Thromboplastinzeit
RIFLE	Risk, Injury, Failure, Loss of kidney function, End-stage kidney disease
RIPC	Remote ischemic preconditioning
SAE	schwerwiegendes unerwünschtes Ereignis (*serious adverse event*)
SNP	single nucleotide polymorphisms
ZKS	Zentrum für Klinische Studien Leipzig

19.4 Teilnehmende Prüfzentren

1. Universitätsklinikum Aachen, Klinik für Anästhesiologie, Pauwelstr. 30, 52074 Aachen
   Prof. Dr. med. Rolf Roissant, PD Dr. med. Steffen Rex

2. Universitätsklinikum Bonn, Klinik und Poliklinik für Anästhesiologie und Operative Intensivmedizin, Sigmund-Freud-Str. 25, 53105 Bonn
   PD Dr. med. Pascal Knüfermann, Dr. med. Maria Wittmann

3. Universitätsklinikum Düsseldorf, Klinik für Anästhesiologie, Moorenstr. 5, 40225 Düsseldorf
   PD Dr. med. Michael Wintherhalter

4. Klinikum der Johann Wolfgang Goethe-Universität, Klinik für Anästhesiologie, Intensivmedizin und Schmerztherapie, Theodor-Stern-Kai 7, 60590 Frankfurt/Main
   Prof. Dr. Dr. med. Kai Zacharowski, Dr. med. Ulrich Strouhal

5. Universitätsklinikum Göttingen, Zentrum Anaesthesiologie, Rettungs- und Intensivmedizin, Robert-Koch-Str. 40, 37099 Göttingen
   Prof. Dr. Dr. med. Martin Bauer, Dr. Ivo Brandes

6. Universitätsklinikum Magdeburg, Universitätsklinik für Anaesthesiologie und Intensivtherapie, Leipziger Str. 44, 39120 Magdeburg
   Prof. Dr. Dr. med. Thomas Hachenberg, Dr. Dr. med. Thomas Schilling

7. Universitätsklinikum Rostock, Klinik & Poliklinik für Anästhesiologie und Intensivtherapie, Schillingallee 35, 18057 Rostock
   PD Dr. med. Jan-Patrick Roesner, Dr. med. Stefan Bergt, Dr. med. Frank Kletzin

8. Universitätsklinikum Schleswig-Holstein, Campus Kiel, Klinik für Anästhesiologie und Operative Intensivmedizin, Schwanenweg 21, 24105 Kiel
   PD Dr. med. Patrick Meybohm (KAI), Prof. Dr. med. Berthold Bein

   Universitätsklinikum Schleswig-Holstein, Campus Kiel, Klinik für Herz- und Gefäßchirurgie, Arnold-Heller-Str. 3, Haus 18, 24105 Kiel,
   Prof. Dr. med. Jochen Cremer

9. Universitätsklinikum Schleswig-Holstein, Campus Lübeck, Klinik für Anästhesiologie und Intensivmedizin, Ratzeburger Allee 160, Haus 13, 23538 Lübeck
   Prof. Dr. med. Matthias Heringlacke

10. Universitätsklinikum Würzburg, Klinik und Poliklinik für Anaesthesiologie, Oberdürrbacher Str. 6, 97080 Würzburg
    PD Dr. med. Markus Lange, Dr. med. Andreas Redel

19.5 European System for Cardiac Operative Risk Evaluation (EuroSCORE)

**Patient**	Alter [pro 1-5 Jahre > 60. Lebensjahr = 1 Punkt]	
	Weibliches Geschlecht [= 1 Punkt]	
	Chronische Lungenerkrankung mit Bronchodilatatoren/ Steroide [= 1 Punkt]	
	Extrakardiale arterielle Gefäßerkrankung [= 2 Punkte]	
	neurologische Erkrankungen mit täglicher Einschränkung [= 2 Punkte]	
	Z.n. Herz-Operation [= 3 Punkte]	
	Serum Kreatinin > 2,27 mg/dl (>200µmol/L) [= 2 Punkte]	
	aktive Endokarditis unter Antibiose [= 3 Punkte]	
	präoperativ instabiler Zustand, z.B. Beatmung, Kammertachykardie, Katecholamine, kardiale Unterstützungssysteme [= 3 Punkte]	
**Herz**	instabile Angina pectoris mit iv Nitraten bis zur Operation [= 2 Punkte]	
	LV-Funktionsstörung moderate (EF 30-50%) [= 1 Punkt]	
	LV-Funktionsstörung schlecht (EF < 30%) [= 3 Punkte]	
	Z.n. Myokardinfarkt < 90 Tagen [= 2 Punkte]	
	Pulmonaler Hypertonus mit PA-Druck$_{syst}$ > 60 mmHg [= 2 Punkte][1]	
**Operation**	Notfall [= 2 Punkte]	
	Kombinationseingriff oder andere Eingriffe außer ACB [= 2 Punkte]	
	Eingriff an thorakaler Aorta, z.B. Aorta ascendens Ersatz [= 3 Punkte]	
	Postinfarkt Septumdefekt/ -ruptur [= 4 Punkte]	

[1]Bei präoperativ nicht gemessenem Pulmonalisdruck und fehlender klinischer Symptomatik des Patienten sind 0 Punkte zu dokumentieren.

19.6 Transösophageale Echokardiographie

Transösophageale Echokardiographie (TEE)

LVOT (PW-Doppler im LVOT)

1. **LVOT Durchmesser (cm)** (Längsachsenblick mitt-ösophageal)
2. **LVOT V_{max} (m/s)** = **V**$_{max}$ im **L**eft **V**entricular **O**utflow **T**ract
3. **LVET (ms)** = **L**eft **V**entricular **E**jection **T**ime

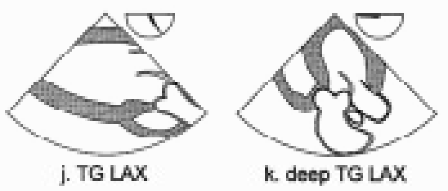

Diastolische Funktion (PW-Doppler in Höhe Mitralklappe)

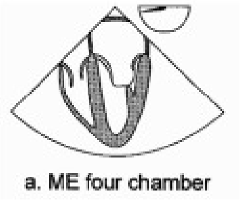

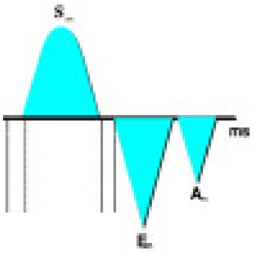

Berechnung vom MPI

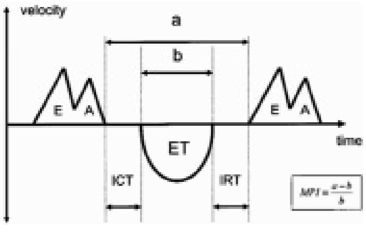

Figure 1. Schematic of the myocardial performance index (MPI). ET = ejection time (aortic valve); E = early diastolic wave (transmitral inflow); A = atrial contraction wave (transmitral inflow); ICT = isovolemic contraction time; IRT = isovolemic relaxation time.

1. **MCO (ms)** = **M**itral **C**lose-to-**O**pen time (Ende A bis Beginn E)
2. **DT_{Early} (ms)** = **D**eceleration **T**ime (Zeit von E_{max}-> Nulllinie)
3. **E/A Ratio** = **E**arly$_{max}$ / **A**trial$_{max}$ **Ratio**
4. **MPI** = **M**yocardial **P**erformance **I**ndex

Ejektionsfraktion (modifiziert nach Simpson)

1. **LVEDV (ml)** = **L**eft **V**entricular **E**nd-**D**iastolic **V**olume
2. **LVESV (ml)** = **L**eft **V**entricular **E**nd-**S**ystolic **V**olume
3. **LVEF (%)** = **L**eft **V**entricular **E**jection **F**raction

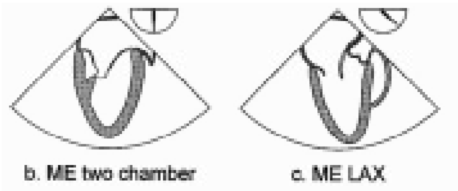

Ejektionsfraktion im M-Mode (Teichholz)

1. **FS (Teich; %)** = **F**ractional **S**hortening
2. **LVEF (Teich; %)** = **L**eft **V**entricular **E**jection **F**raction

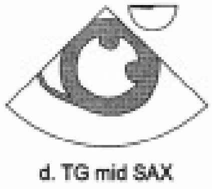

© PD Dr. Patrick Meybohm / E-mail: meybohm@anaesthesie.uni-kiel.de

19.7 Klassifikation unerwünschter Ereignisse (AE/ SAE)

19.7.1 Beurteilung der Intensität

Die Beurteilung der Intensität erfolgt entsprechend der CTCAE V3.0

**Mild Adverse Event**	**Leichtes Ereignis**<br>• keine spezielle medizinische Intervention notwendig,<br>• ausschließlich asymptomatische Laborergebnisse oder Röntgenbefunde,<br>• geringe medizinische Relevanz
**Moderate Adverse Event**	**Moderates/mittelschweres Ereignis**<br>• minimale medizinische Intervention erforderlich bzw. Intervention lokal begrenzt,<br>• ausschließlich nichtinvasive Maßnahmen (z.B. Wickel) notwendig
**Severe and undesirable Adverse Event**	**Schweres und nicht erwünschtes Ereignis**<br>• signifikante Symptome, die einen Krankenhausaufenthalt oder invasive Interventionen erfordern,<br>• z.B. Transfusionen, elektive interventionelle radiologische Maßnahmen, therapeutische Endoskopie oder Operation
**Life-threatening or disabling Adverse Event**	**Lebensbedrohliches oder zur Behinderung führendes Ereignis**<br>• verschärft durch akute, lebensbedrohliche Komplikationen des Stoffwechsels oder des Kreislaufsystems, z.B. Kreislaufkollaps, Hämorrhagie, Sepsis<br>• lebensbedrohliche physiologische Konsequenzen,<br>Notwendigkeit intensiver Betreuung, umgehender invasiver, interventioneller oder radiologischer Maßnahmen, therapeutische Endoskopie oder Operation
**Death related to Adverse Event**	**Tod als Folge des AE**

19.7.2 Beurteilung des Kausalzusammenhanges

Der Prüfer muss beurteilen, ob seiner Meinung nach das Auftreten des unerwünschten Ereignisses in einem kausalen Zusammenhang mit der Studienintervention steht. Dabei ist die unten genannte Klassifikation anzuwenden.

Als **Möglich** wird der Zusammenhang bewertet, wenn eines der folgenden Kriterien nach WHO-UMC erfüllt ist:

- plausibler zeitlicher Zusammenhang, **und** kann nicht durch Begleiterkrankung oder andere Produkte erklärt werden, **und** positive Reaktion auf Absetzen, **und**

bekannte pharmakologische oder phänomenologische Reaktion **und** positive Wiedergabe des Produktes, wenn notwendig

- Begründeter zeitlicher Zusammenhang, **und** Erklärung durch Begleiterkrankung oder andere Produkte unwahrscheinlich, **und** Positive Reaktion auf Absetzen
- Begründeter zeitlicher Zusammenhang, **aber** Erklärung durch andere Produkte oder Begleiterkrankung möglich, Informationen über Absetzen sind unvollständig oder unklar
- Weitere Informationen notwendig, um Beurteilung exakt vorzunehmen
- Beurteilung nicht möglich, da die Informationen unzureichend oder widersprüchlich sind

Als **Nicht Möglich** wird der Zusammenhang bewertet, wenn das folgende Kriterium nach WHO-UMC erfüllt ist:

Zeitlicher Zusammenhang macht den kausalen Zusammenhang unwahrscheinlich, **und** andere Produkte oder zugrunde liegende Erkrankungen liefern plausible Erklärung

19.7.3 Ausgang des unerwünschten Ereignisses

Der Ausgang eines unerwünschten Ereignisses wird wie folgt klassifiziert:

- wiederhergestellt
- Besserung
- noch nicht wiederhergestellt
- wiederhergestellt mit Folgeschäden
- tödlicher Ausgang*
- unbekannter Ausgang

*Achtung: Der Tod eines Patienten ist an sich kein Ereignis, sondern dessen Ergebnis. Das Ereignis, welches zum Tod des Patienten geführt hat, muss vollständig dokumentiert und gemeldet werden, auch wenn der Tod erst vier Wochen nach Ende der Studienbehandlung aufgetreten ist und unabhängig davon, ob es einen Zusammenhang mit der Therapie gibt oder nicht.

Remote ischaemic preconditioning for heart surgery

The study design for a multi-center randomized double-blinded controlled clinical trial- the RIPHeart-Study

Aims Transient ischaemia of non-vital tissue has been shown to enhance the tolerance of remote organs to cope with a subsequent prolonged ischaemic event in a number of clinical conditions, a phenomenon known as remote ischaemic preconditioning (RIPC). However, there remains uncertainty about the efficacy of RIPC in patients undergoing cardiac surgery. The purpose of this report is to describe the design and methods used in the 'Remote Ischaemic Preconditioning for Heart Surgery (RIPHeart)-Study'.

Methods We are conducting a prospective, randomized, double-blind, multicentre, controlled trial including 2070 adult cardiac surgical patients. All types of surgery in which cardiopulmonary bypass is used will be included. Patients will be randomized either to the RIPC group receiving four 5 min cycles of transient upper limb ischaemia/reperfusion or to the control group receiving four cycles of blood pressure cuff inflation/deflation at a dummy arm. The primary endpoint is a composite outcome (all-cause mortality, non-fatal myocardial infarction, any new stroke, and/or acute renal failure) until hospital discharge.

Conclusion The RIPHeart-Study is a multicentre trial to determine whether RIPC may improve clinical outcome in cardiac surgical patients.
This trial is registered at www.clinicaltrials.gov (Identifier: NCT01067703).

Keywords ischemic preconditioning, clinical study.

Introduction

Transient ischaemia of non-vital tissue (e.g. skeletal muscles) has been shown to enhance the tolerance of remote vital organs (heart, brain, and kidney) to cope with a subsequent prolonged ischaemic event in a number of clinical conditions, a phenomenon known as remote ischaemic preconditioning (RIPC).

In previous pilot studies, RIPC has been shown to reduce myocardial injury in coronary artery surgery,[1,2] congenital heart surgery,[3] and non-cardiac surgery of high-risk patients.[4] Nevertheless, this may not necessarily translate into a readily demonstrable clinical benefit. Thus, a large randomized study with a robust and relevant clinical endpoint is required.[5]

Methods

This is a prospective, randomized, double-blind, multicentre, parallel group controlled trial in 2070 adult cardiac surgical patients. All types of elective surgery in which cardiopulmonary bypass is used will be included. The trial work flow is presented in Supplementary material online, *Figure S1*.

Study population

Patients (age $\geq$18 years) after written informed consent scheduled for all types of elective cardiovascular surgery in which cardiopulmonary bypass is used are included. Exclusion criteria comprise surgery-related criteria (off-pump heart surgery, concomitant carotid surgery, minimal-invasive surgery, selective antegrade cerebral perfusion, previous heart surgery, aorta descendent surgery), cardiac conditions (myocardial infarction up to 7 days, ejection fraction $<$30%, previous atrial fibrillation up to 6 months, drug therapy with antiarrhythmic agents, implanted pacemaker or defibrillator, instable angina pectoris), and others [stroke up to 2 months, renal failure, liver failure, severe alcohol abuse, severe chronic obstructive pulmonary disease, drug therapy with sulfonylureas and nicorandil (preconditioning-blocking and preconditioning-mimetic medication, respectively), acute infection with antibiotic therapy, severe peripheral artery occlusive disease (Fontaine stages 3 and 4), previous serious neurological illness, and/or heparin-induced thrombocytopenia Type II].

Anaesthesia and management of cardiopulmonary bypass

Total intravenous anaesthesia will be performed in all patients, as volatile anaesthetic agents have been shown to induce preconditioning-like effects, thereby reducing myocardial ischaemia.[6,7] As only 7 out of 14 recruiting centres are able to run a vaporizer attached to the cardiopulmonary bypass circuit to administer volatile anaesthetics, we use a total intravenous anaesthesia technique to avoid a potential impact on generalizability of our data. According to a recent review,[8] we standardize the management of cardiopulmonary bypass as follows: the use of non-pulsatile cardiopulmonary bypass, the mean arterial blood pressure of 60–70 mmHg, haematocrit values 25–30%, α-stat acid–base management to regulate carbon dioxide tension, the use of arterial line filters, and blood glucose levels $<$200 mg/dL.

Downloaded from http://eurheartj.oxfordjournals.org/ at Bibliothek des Mathematischen Seminars, Universitaet Frankfurt on June 16, 2012

Randomization

Patients are randomized to the RIPC or the control group. Randomization is performed centrally by the Clinical Trial Centre Leipzig. The randomization is stratified for (i) centres and (ii) individual risk for perioperative mortality using the European System for Cardiac Operative Risk Evaluation (EuroSCORE).[9] We use a cut-off value of higher than 5% for a 'Predicted mortality by logistic EuroSCORE' to stratify randomization of so-called 'high-risk patients'.[10] An online checklist for individual risk factors is used to calculate the logistic EuroSCORE at the time of registration. After written informed consent, the Data Management (Clinical Trial Centre Leipzig) is contacted via an Internet-based randomization tool.

Blinding/intervention

To ensure blinding, surgical drapes are used to cover both blood pressure cuffs of the patient's arm and the dummy arm. Blinding concerns (i) the individual patient, (ii) staff involved in intraoperative (anaesthesia and cardiac surgery team) and perioperative care (intensive care unit), (iii) investigators obtaining data, performing neurocognitive assessment, follow-up visits, and documentation, and (iv) the endpoint committee. Study intervention (RIPC or control) is performed by un-blinded staff. RIPC will be induced by four cycles of upper limb ischaemia (5 min blood pressure cuff inflation to 200 mmHg, at least a pressure 15 mmHg higher than the systolic arterial pressure and 5 min cuff deflation). In patients assigned to the control group, a dummy arm will be used for four cycles of 5 min blood pressure cuff inflation to 200 mmHg and 5 min cuff deflation.

Endpoints

Primary endpoints

The primary endpoint is defined as a composite of all-cause mortality, non-fatal myocardial infarction, any new stroke, and/or acute renal failure until hospital discharge (with a maximum period of 14 days after surgery).

(i) All-cause mortality.
(ii) According to the ESC/ACC/AHA/WHF Task Force for the Redefinition of Myocardial Infarction,[11] non-fatal myocardial infarction is considered according to a Type V myocardial infarction as follows: biomarker values more than five times the 99th percentile of the normal reference range combined with new pathological Q-waves or new left bundle branch block within the first 72 h, standard clinical criteria for myocardial infarction from 72 h on, or myocardial infarction diagnosed at autopsy.
(iii) Stroke is defined by any new, temporary or permanent, focal or global neurological deficit,[12] or evidence of stroke on autopsy and will be evaluated according to the NIH stroke scale ($\geq$4 points) documented preoperatively and at hospital discharge.
(iv) Acute renal failure is defined by any serum creatinine greater than or equal to two-fold increase from baseline within the first 48 h, urine output $<$0.5 mL/kg/h for $>$12 h within the first 48 h (RIFLE injury),[13] use of renal replacement therapy, or evidence of renal failure on autopsy.

Secondary endpoints

- The occurrence of any individual component of the composite at 30 days and 3 and 12 months after surgery (phone interview).
- The duration of ventilator support, length of stay on the intensive care unit, and total hospital stay.
- Troponin T and serum creatinine (preoperative, 6, 12, 24, and 48 h after surgery).
- Cumulative vasopressor dose and inotropic support (within max. 7 days after surgery).
- New onset of atrial fibrillation (within 4 days after surgery).
- Incidence of postoperative delirium will be assessed with the CAM-ICU score[14] (preoperative, 24, 48, 72, and 96 h after surgery).
- Optional: in a subgroup of patients, myocardial function will be assessed by intraoperative transoesophageal echocardiography 30 $\pm$ 5 min before and 30 $\pm$ 5 min after cardiopulmonary bypass.[15]
- Optional: neurocognitive function—a subgroup of patients will undergo robust neuropsychological tests preoperative, 5–7 days, and 3 months after surgery.[16] A 'core battery' of 10 tests will include four main domains: (i) memory, (ii) attention, (iii) motor skills, and (iv) executive function.

Ethics

Study protocol, patient information, and informed consent were approved by the Ethics Committees of the University of Kiel and of all participating centres. Each patient must give written informed consent to participate in the study. Data collection is performed pseudonymously.

Statistical analysis

Assumed event rates in the control group are based on the German data by the 'Bundesgeschäftsstelle für Qualitätssicherung GmbH' (www.bqs-outcome.de/2007/ergebnisse/leistungsbereiche; assessed 3 May 2009) for all-cause mortality (ranging from 3.2 to 6.1%), any new stroke (1.3–2.3%), and acute renal failure (3.8–6.9%) depending on the type of surgery. Assuming a case-mix in the registry (72% isolated coronary artery graft surgery, 17% isolated aortic valve replacement, and 12% combined operations), we calculated a weighted mean of 3.7% for all-cause mortality, 1.5% for any new stroke, and 4.2% for acute renal failure. The non-fatal myocardial infarction event rate was not reported in the above data registry. A compilation of relevant studies that reported myocardial infarction event rates from 2.4 to 5.1,[7] 2.8 to 5.4,[17] 3.9 to 4.2,[18] and 6.2 to 7.9[19] results in a median rate of 4.7%. The sum of the four components for the composite endpoint is 14.1%. Considering the fact that a small proportion of patients dying after stroke or myocardial infarction count more than once in these figures and that study populations tend to be at lower risk than unselected patient populations, we conservatively assume a baseline rate of 12% for the primary endpoint in the control group. Pharmacological studies in the field of cardiac surgery reported risk reductions ranging from 25 to 70%.[17,20–22] As a cheap, simple, and safe intervention is investigated in our study, we target at 33% risk reduction to an event rate of 8% in the RIPC group.

Group sample sizes of 931 and 931 achieve 80% power to detect a difference of 4% using a two-sided χ^2 test with continuity correction and with a significance level of 0.05. We used a commercial available statistic software (NCSS 2007 and Power Analysis and Sample Size 2008; Kaysville, UT, USA) for sample size and power calculation. To account for 10% follow-up loss, 104 patients per group will be additionally enrolled, resulting in a total of 1.035 patients per group.

Logistic regression adjusting for EuroSCORE, diabetes mellitus status, concomitant treatment with statins, and incorporating centres as random effect will be used to estimate the treatment effect on the odds ratio scale with two-sided 95% confidence intervals provided. Additionally, the endpoints of the study will be evaluated by multiple regression modelling techniques to assess/adjust associations with other baseline clinical factors. No formal interim analyses are planned.

Downloaded from http://eurheartj.oxfordjournals.org/ at Bibliothek des Mathematischen Seminars, Universitaet Frankfurt on June 16, 2012

Clinical study monitoring

Pre-study, initiation, and close-out visits will be performed in all trial sites. The trial sites will be regularly visited approximately every 3 months during the recruitment and follow-up. The scope of these visits is to check compliance of the trial sites with the study protocol and good clinical practice rules. In addition, source data verification of the key data (eligibility criteria, intervention, outcome measures) will be performed routinely in a random sample of 50% of the patients. Central monitoring will include a timely query management process based on consistency and plausibility checks supervised by the trial biometrician.

Discussion

Cardiac surgery with cardiopulmonary bypass is generally associated with a predictable incidence of myocardial, neurological, and renal ischaemia/reperfusion injury leading to an increased risk of post-operative myocardial stunning, neurological deficits, and acute renal failure.

In the last 6 years, RIPC has been rapidly translated from experimental discovery to encouraging proof-of-principle human studies demonstrating the protection of the heart, lung, and kidney by using surrogate endpoints.[1,3–5,23–33] Despite these early promises, two recent trials of RIPC in cardiac surgery failed to demonstrate a significant effect of RIPC on troponin release.[34,35] So far, there remains uncertainty about the efficacy of RIPC in cardiovascular surgical patients. Importantly, most of the previous clinical studies that showed beneficial effects of RIPC were single-blinded (lacking blinding members of the anaesthesia and cardiac surgery team), included mainly patients with isolated coronary artery bypass surgery, and were not standardized in terms of the anaesthesia regime. In contrast, in our study, we use a double-blind design with a dummy arm for control intervention, allow a much more generalizability to patients with isolated coronary surgery and complex heart surgery, and use total intravenous anaesthesia to avoid a potential impact of volatile anaesthetic.

In conclusion, the RIPHeart-Study is a prospective, randomized, double-blind, multicentre trial to determine whether RIPC may improve clinical outcome in more than 2000 cardiovascular surgical patients.

Supplementary material

Supplementary material is available at *European Heart Journal* online.

Funding

This work is supported by the German Research Foundation (grant no. ME 3559/1-1).

Conflict of interest: none declared.

Authors:
Patrick Meybohm[1, 18], Kai Zacharowski[1], Jochen Cremer[2], Jan Roesner[3], Frank Kletzin[3], Gereon Schaelte[4], Marc Felzen[4], Ulrich Strouhal[5], Christian Reyher[5], Matthias Heringlake[6], Julika Schön[6], Ivo Brandes[7], Martin Bauer[7], Pascal Knuefermann[8], Maria Wittmann[8], Thomas Hachenberg[9], Thomas Schilling[9], Thorsten Smul[10], Sonja Maisch[10], Michael Sander[11], Tobias Moormann[11], Andreas Boening[12], Markus A. Weigand[12], Rita Laufenberg[13], Christian Werner[13], Michael Winterhalter[14], Tanja Treschan[14], Sebastian N. Stehr[15], Konrad Reinhart[15], Dirk Hasenclever[17], Oana Brosteanu[17], Berthold Bein[18], and the RIP Heart-Study Investigator Group*

Authors' Affiliations:
[1]Clinic of Anaesthesiology, Intensive Care Medicine and Pain Therapy, University Hospital Frankfurt, Frankfurt am Main, Germany
[2]Department of Cardiovascular Surgery, University Hospital Schleswig-Holstein, Campus Kiel, Germany
[3]Clinic of Anaesthesiology and Intensive Care Medicine, University Hospital Rostock, Rostock, Germany
[4]Department of Anaesthesiology, University Hospital Aachen, Aachen, Germany
[5]Department of Anaesthesiology, University Hospital Luebeck, Luebeck, Germany
[6]Department of Anaesthesiology and Intensive Care Medicine, University Hospital Bonn, Bonn, Germany
[7]Department of Anaesthesiology and Intensive Care Medicine, University Hospital Goettingen, Germany
[8]Department of Anaesthesiology and Intensive Care Medicine, University Hospital Duesseldorf, Germany
[9]Department of Anaesthesiology, University Hospital Magdeburg, Germany
[10]Department of Anaesthesiology, University Hospital Wuerzburg, Wuerzburg, Germany
[11]Department of Anaesthesiology and Intensive Care Medicine, Charité-Universitätsmedizin Berlin, Campus Charité Mitte, Berlin, Germany
[12]Department of Cardiovascular Surgery, University of Giessen, Germany
[13]Department of Anesthesiology, Medical Center of Johannes Gutenberg-University, Mainz, Germany
[14]Department of Anesthesiology University Düsseldorf, Germany
[15]Department of Anesthesiology and Intensive Care Medicine, Jena University Hospital, Jena, Germany
[16]Institute for Medical Informatics, Statistics and Epidemiology, University Leipzig, Germany
[17]Clinical Trial Center, University Leipzig, Leipzig, Germany
[18]Department of Anaesthesiology and Intensive Care Medicine, University Hospital Schleswig-Holstein, Campus Kiel, Germany

*__RIPHeart-Study Investigators:__ Christian Stoppe, Bernadette Krauss, Georg-Friedrich Eberhardt, Marc Coburn (Aachen), Katharina Chalk (Berlin), Georg Baumgarten, Olaf Boehm, Thomas Recht, Kerstin Köhlhoff, Florian Kessler, Thorsten Bähner, Eli Awlakpui (Bonn), Bertram Scheller, Alexander Koch, Tobias Bingold, Gerda Fingerhut (Frankfurt), Gerold Goerlach, Matthias Wollbrueck (Giessen), Julia Strauchmann (Goettingen), Kai U. Morsbach (Jena), Jens Scholz, Jochen Renner, Ole Broch, Matthias Gruenewald, Martin Albrecht, Martina Green, Carola Wehlen, Corina Messmer, Christina Schuldt (Kiel), Vera Schleicher, Silke Hauer, Holger Bogatsch, Bianca Scholze (Leipzig), Hans-Hinrich Sievers, Thorsten Hanke, Hermann Heinze, Silke Olsson, Joachym Ettel, Michael von der Ecken, Pascal Stitz, Joachim Bremer (Luebeck), Susanne Mauff (Mainz), Angela Alms, Stefan Bergt (Rostock).

Corresponding author: Patrick Meybohm, Clinic of Anaesthesiology, Intensive Care Medicine and Pain Therapy, University Hospital Frankfurt, Theodor-Stern-Kai 7, 60590 Frankfurt am Main, Germany. Tel: +49 69 6301 5998, Fax: +49 69 6301 5881, Email: patrick.meybohm@kgu.de

References

1. Hausenloy DJ, Mwamure PK, Venugopal V, Harris J, Barnard M, Grundy E, Ashley E, Vichare S, Di Salvo C, Kolvekar S, Hayward M, Keogh B,

Downloaded from http://eurheartj.oxfordjournals.org/ at Bibliothek des Mathematischen Seminars, Universitaet Frankfurt on June 16, 2012

MacAllister RJ, Yellon DM. Effect of remote ischaemic preconditioning on myocardial injury in patients undergoing coronary artery bypass graft surgery: a randomised controlled trial. *Lancet* 2007;**370**:575–579.
2. Gunaydin B, Cakici I, Soncul H, Kalaycioglu S, Cevik C, Sancak B, Kanzik I, Karadenizli Y. Does remote organ ischaemia trigger cardiac preconditioning during coronary artery surgery? *Pharmacol Res* 2000;**41**:493–496.
3. Cheung MM, Kharbanda RK, Konstantinov IE, Shimizu M, Frndova H, Li J, Holtby HM, Cox PN, Smallhorn JF, Van Arsdell GS, Redington AN. Randomized controlled trial of the effects of remote ischemic preconditioning on children undergoing cardiac surgery: first clinical application in humans. *J Am Coll Cardiol* 2006;**47**:2277–2282.
4. Ali ZA, Callaghan CJ, Lim E, Ali AA, Nouraei SA, Akthar AM, Boyle JR, Varty K, Kharbanda RK, Dutka DP, Gaunt ME. Remote ischemic preconditioning reduces myocardial and renal injury after elective abdominal aortic aneurysm repair: a randomized controlled trial. *Circulation* 2007;**116**:I98–105.
5. Ludman AJ, Hausenloy DJ, Venugopal V, Yellon DM. Premature meta-analysis of remote ischemic preconditioning. *Am J Cardiol* 2009;**103**:893–894.
6. De Hert SG, Van der Linden PJ, Cromheecke S, Meeus R, ten Broecke PW, De Blier IG, Stockman BA, Rodrigus IE. Choice of primary anesthetic regimen can influence intensive care unit length of stay after coronary surgery with cardiopulmonary bypass. *Anesthesiology* 2004;**101**:9–20.
7. Landoni G, Biondi-Zoccai GG, Zangrillo A, Bignami E, D'Avolio S, Marchetti C, Calabro MG, Fochi O, Guarracino F, Tritapepe L, De Hert S, Torri G. Desflurane and sevoflurane in cardiac surgery: a meta-analysis of randomized clinical trials. *J Cardiothorac Vasc Anesth* 2007;**21**:502–511.
8. Murphy GS, Hessel EA 2nd, Groom RC. Optimal perfusion during cardiopulmonary bypass: an evidence-based approach. *Anesth Analg* 2009;**108**:1394–1417.
9. Nashef SA, Roques F, Michel P, Gauducheau E, Lemeshow S, Salamon R. European system for cardiac operative risk evaluation (EuroSCORE). *Eur J Cardiothorac Surg* 1999;**16**:9–13.
10. Gogbashian A, Sedrakyan A, Treasure T. EuroSCORE: a systematic review of international performance. *Eur J Cardiothorac Surg* 2004;**25**:695–700.
11. Thygesen K, Alpert JS, White HD, Jaffe AS, Apple FS, Galvani M, Katus HA, Newby LK, Ravkilde J, Chaitman B, Clemmensen PM, Dellborg M, Hod H, Porela P, Underwood R, Bax JJ, Beller GA, Bonow R, Van der Wall EE, Bassand JP, Wijns W, Ferguson TB, Steg PG, Uretsky BF, Williams DO, Armstrong PW, Antman EM, Fox KA, Hamm CW, Ohman EM, Simoons ML, Poole-Wilson PA, Gurfinkel EP, Lopez-Sendon JL, Pais P, Mendis S, Zhu JR, Wallentin LC, Fernandez-Aviles F, Fox KM, Parkhomenko AN, Priori SG, Tendera M, Voipio-Pulkki LM, Vahanian A, Camm AJ, De Caterina R, Dean V, Dickstein K, Filippatos G, Funck-Brentano C, Hellemans I, Kristensen SD, McGregor K, Sechtem U, Silber S, Tendera M, Widimsky P, Zamorano JL, Morais J, Brener S, Harrington R, Morrow D, Lim M, Martinez-Rios MA, Steinhubl S, Levine GN, Gibler WB, Goff D, Tubaro M, Dudek D, Al-Attar N. Universal definition of myocardial infarction. *Circulation* 2007;**116**:2634–2653.
12. Sacco RL, Adams R, Albers G, Alberts MJ, Benavente O, Furie K, Goldstein LB, Gorelick P, Halperin J, Harbaugh R, Johnston SC, Katzan I, Kelly-Hayes M, Kenton EJ, Marks M, Schwamm LH, Tomsick T. Guidelines for prevention of stroke in patients with ischemic stroke or transient ischemic attack: a statement for healthcare professionals from the American Heart Association/American Stroke Association Council on Stroke: co-sponsored by the Council on Cardiovascular Radiology and Intervention: the American Academy of Neurology affirms the value of this guideline. *Circulation* 2006;**113**:e409–449.
13. Bellomo R, Ronco C, Kellum JA, Mehta RL, Palevsky P. Acute renal failure—definition, outcome measures, animal models, fluid therapy and information technology needs: the Second International Consensus Conference of the Acute Dialysis Quality Initiative (ADQI) Group. *Crit Care* 2004;**8**:R204–212.
14. Ely EW, Inouye SK, Bernard GR, Gordon S, Francis J, May L, Truman B, Speroff T, Gautam S, Margolin R, Hart RP, Dittus R. Delirium in mechanically ventilated patients: validity and reliability of the confusion assessment method for the intensive care unit (CAM-ICU). *J Am Med Assoc* 2001;**286**:2703–2710.
15. Shanewise JS, Cheung AT, Aronson S, Stewart WJ, Weiss RL, Mark JB, Savage RM, Sears-Rogan P, Mathew JP, Quinones MA, Cahalan MK, Savino JS. ASE/SCA guidelines for performing a comprehensive intraoperative multiplane transesophageal echocardiography examination: recommendations of the American Society of Echocardiography Council for Intraoperative Echocardiography and the Society of Cardiovascular Anesthesiologists Task Force for Certification in Perioperative Transesophageal Echocardiography. *J Am Soc Echocardiogr* 1999;**12**:884–900.
16. Murkin JM, Newman SP, Stump DA, Blumenthal JA. Statement of consensus on assessment of neurobehavioral outcomes after cardiac surgery. *Ann Thorac Surg* 1995;**59**:1289–1295.
17. Mangano DT. Aspirin and mortality from coronary bypass surgery. *N Engl J Med* 2002;**347**:1309–1317.
18. Liakopoulos OJ, Choi YH, Haldenwang PL, Strauch J, Wittwer T, Dorge H, Stamm C, Wassmer G, Wahlers T. Impact of preoperative statin therapy on adverse postoperative outcomes in patients undergoing cardiac surgery: a meta-analysis of over 30,000 patients. *Eur Heart J* 2008;**29**:1548–1559.
19. Collard CD, Body SC, Shernan SK, Wang S, Mangano DT. Preoperative statin therapy is associated with reduced cardiac mortality after coronary artery bypass graft surgery. *J Thorac Cardiovasc Surg* 2006;**132**:392–400.
20. Magee MJ, Alexander JH, Hafley G, Ferguson TB Jr, Gibson CM, Harrington RA, Peterson ED, Califf RM, Kouchoukos NT, Herbert MA, Mack MJ. Coronary artery bypass graft failure after on-pump and off-pump coronary artery bypass: findings from PREVENT IV. *Ann Thorac Surg* 2008;**85**:494–499.
21. Pan W, Pintar T, Anton J, Lee VV, Vaughn WK, Collard CD. Statins are associated with a reduced incidence of perioperative mortality after coronary artery bypass graft surgery. *Circulation* 2004;**110**:II45–49.
22. Karkouti K, Wijeysundera DN, Beattie WS. Risk associated with preoperative anemia in cardiac surgery: a multicenter cohort study. *Circulation* 2008;**117**:478–484.
23. Loukogeorgakis SP, Williams R, Panagiotidou AT, Kolvekar SK, Donald A, Cole TJ, Yellon DM, Deanfield JE, MacAllister RJ. Transient limb ischemia induces remote preconditioning and remote postconditioning in humans by a K(ATP)-channel dependent mechanism. *Circulation* 2007;**116**:1386–1395.
24. Wang L, Oka N, Tropak M, Callahan J, Lee J, Wilson G, Redington A, Caldarone CA. Remote ischemic preconditioning elaborates a transferable blood-borne effector that protects mitochondrial structure and function and preserves myocardial performance after neonatal cardioplegic arrest. *J Thorac Cardiovasc Surg* 2008;**136**:335–342.
25. Hoole SP, Heck PM, Sharples L, Khan SN, Duehmke R, Densem CG, Clarke SC, Shapiro LM, Schofield PM, O'Sullivan M, Dutka DP. Cardiac Remote Ischemic Preconditioning in Coronary Stenting (CRISP Stent) Study: a prospective, randomized control trial. *Circulation* 2009;**119**:820–827.
26. Botker HE, Kharbanda R, Schmidt MR, Bottcher M, Kaltoft AK, Terkelsen CJ, Munk K, Andersen NH, Hansen TM, Trautner S, Lassen JF, Christiansen EH, Krusell LR, Kristensen SD, Thuesen L, Nielsen SS, Rehling M, Sorensen HT, Redington AN, Nielsen TT. Remote ischaemic conditioning before hospital admission, as a complement to angioplasty, and effect on myocardial salvage in patients with acute myocardial infarction: a randomised trial. *Lancet* 2010;**375**:727–734.
27. Venugopal V, Hausenloy DJ, Ludman A, Di Salvo C, Kolvekar S, Yap J, Lawrence D, Bognolo J, Yellon DM. Remote ischaemic preconditioning reduces myocardial injury in patients undergoing cardiac surgery with cold-blood cardioplegia: a randomised controlled trial. *Heart* 2009;**95**:1567–1571.
28. Thielmann M, Kottenberg E, Boengler K, Raffelsieper C, Neuhaeuser M, Peters J, Jakob H, Heusch G. Remote ischemic preconditioning reduces myocardial injury after coronary artery bypass surgery with crystalloid cardioplegic arrest. *Basic Res Cardiol* 2010;**105**:657–664.
29. Walsh SR, Sadat U, Boyle JR, Tang TY, Lapsley M, Norden AG, Gaunt ME. Remote ischemic preconditioning for renal protection during elective open infrarenal abdominal aortic aneurysm repair: randomized controlled trial. *Vasc Endovasc Surg* 2010;**44**:334–340.
30. Walsh SR, Tang TY, Sadat U, Gaunt ME. Remote ischemic preconditioning in major vascular surgery. *J Vasc Surg* 2009;**49**:240–243.
31. Hu S, Dong HL, Li YZ, Luo ZJ, Sun L, Yang QZ, Yang LF, Xiong L. Effects of remote ischemic preconditioning on biochemical markers and neurologic outcomes in patients undergoing elective cervical decompression surgery: a prospective randomized controlled trial. *J Neurosurg Anesthesiol* 2010;**22**:46–52.
32. Dong HL, Zhang Y, Su BX, Zhu ZH, Gu QH, Sang HF, Xiong L. Limb remote ischemic preconditioning protects the spinal cord from ischemia-reperfusion injury: a newly identified nonneuronal but reactive oxygen species-dependent pathway. *Anesthesiology* 2010;**112**:881–891.
33. Hoole SP, Khan SN, White PA, Heck PM, Kharbanda RK, Densem CG, Clarke SC, Shapiro LM, Schofield PM, O'Sullivan M, Dutka DP. Remote ischaemic preconditioning does not attenuate ischaemic left ventricular dysfunction in humans. *Eur J Heart Fail* 2009;**11**:497–505.
34. Hong DM, Mint JJ, Kim JH, Sohn IS, Lim TW, Lim YJ, Bahk JH, Jeon Y. The effect of remote ischaemic preconditioning on myocardial injury in patients undergoing off-pump coronary artery bypass graft surgery. *Anaesth Intensive Care* 2010;**38**:924–929.
35. Rahman IA, Mascaro JG, Steeds RP, Frenneaux MP, Nightingale P, Gosling P, Townsend P, Townend JN, Green D, Bonser RS. Remote ischemic preconditioning in human coronary artery bypass surgery: from promise to disappointment? *Circulation* 2010;**122**:S53–S59.

Downloaded from http://eurheartj.oxfordjournals.org/ at Bibliothek des Mathematischen Seminars, Universitaet Frankfurt on June 16, 2012

CardioPulse contact: Andros Tofield, MD, FRCS, FACEP, Managing Editor CardioPulse, *EHJ*. Email: docandros@bluewin.ch

Zentrum für Klinische Studien Leipzig · KKS

Amendment Nr. 1

Datum der Fassung: 07.04.2014

Status der Fassung: Final 1.0

Zum Prüfplan:

Endfassung vom *17.10.2010*

Prospektive, randomisierte, doppelblinde, multizentrische klinische Prüfung zum Einfluss der ischämischen Fern- (Remote) Präkonditionierung bei herzchirurgischen Patienten

Kurztitel:

RIPHeart-Studie

Gefördert von der Deutschen Forschungsgemeinschaft (DFG)

Studienleitung	Biometrie
**PD Dr. med. Patrick Meybohm**<br>**Prof. Dr. Dr. med. Kai Zacharowski**<br>Klinik für Anästhesiologie, Intensivmedizin und Schmerztherapie<br>Klinikum der J.-W. von Goethe-Universität<br>Theodor-Stern-Kai 7<br>60590 Frankfurt am Main<br><br>**Prof. Dr. med. Berthold Bein**<br>Klinik für Anästhesiologie und Operative Intensivmedizin<br>**Prof. Dr. med. Jochen Cremer**<br>Klinik für Herz- und Gefäßchirurgie<br>Universitätsklinikum Schleswig-Holstein, Campus Kiel<br>Schwanenweg 21<br>24105 Kiel	**Dr. Dirk Hasenclever**<br>Institut für Medizinische Informatik, Statistik und Epidemiologie, Universität Leipzig<br>Härtelstraße 16-18, 04107 Leipzig<br><br>Koordination<br><br>**Holger Bogatsch**<br>Zentrum für Klinische Studien Leipzig - KKS<br>Universität Leipzig<br>Härtelstraße 16-18, 04107 Leipzig

Förderkennzeichen: ME 3559/1-1

1. Amendment

1. Änderung

Ergänzter Abschnitt in Kapitel 3 Studienbeschreibung: 3.5.3 Vorzeitige Beendigung der Rekrutierung

Neuer Text:

Die Studienrekrutierung für die RIPHeart-Studie wird mit Erreichen einer Fallzahl von N= 1400 randomisierten Patienten eingestellt:

- Sobald N=1400 Patienten randomisiert sind, werden die Prüfzentren zeitnah informiert, dass das Screening für RIPHeart beendet ist.
- Patienten, die zu diesem Zeitpunkt bereits gefragt worden sind, an der Studie teilzunehmen, können noch randomisiert werden.
- Die Randomisation wird daher zwei Wochen nach Benachrichtigung über das Rekrutierungsende geschlossen.

Grund der Änderung:

1. Die Rekrutierung verläuft langsamer als geplant.
    - Nach der geplanten Rekrutierungszeit von maximal 3 Jahren waren Anfang Januar 2014 nur 1227 Patienten randomisiert.
    - Die Rekrutierungsrate hat sich bei ca. 35 Patienten/Monat stabilisiert. Geplant waren ca. 60 Patienten /Monat.
    - Bei Fortschreibung der derzeitigen Rekrutierungsrate würde die Zielfallzahl von N=2070 erst nach insgesamt 5 Jahren Ende 2015 erreicht.

2. Die Finanzierung der 3 jährigen Rekrutierungsphase läuft nach bereits einmaliger kostenneutraler Verlängerung Ende Juni 2014 aus.

3. Der primäre zusammengesetzte Endpunkt ist die Komplikationsrate (Tod, Myokardinfarkt, Schlaganfall, Nierenversagen). Die nötige Fallzahl hängt stark von der Höhe der Gesamtkomplikationsrate ab.
    - In der Studienplanung geht ERICCA von einer deutlich höheren Komplikationsrate (20% statt 12% im Kontrollarm) als RIPHeart aus.
    - Unsere Protokollannahme von 12% Komplikationen im Kontrollarm beruhte im Wesentlichen auf Daten der Bundesgeschäftsstelle für Qualitätssicherung GmbH (BQS-Bundesauswertung 2008). Darin werden Meldungen aus der klinischen Routine verwendet.
    - Die Endpunktkomponente Myokardinfarkt im RIPHeart Protokoll basiert hingegen für die ersten 72h nach Operation auf der internationalen Definition der „*Joint Task Force of the European Society of Cardiology/ American College of Cardiology/ American Heart Association for the Redefinition of Myocardial Infarction*".

- Diese beruht im Wesentlichen auf dem Auftreten neuer Linksschenkelblöcke oder q-Wellen im EKG nach der Operation.
- Gemäß RIPHeart Protokoll werden alle EKGs vor OP sowie 24h und 72h nach OP referenzbegutachtet.
- Dies lässt eine Erhöhung der Myokardinfarktrate erwarten.

4. Daher wurde von der Studienleitung beschlossen, die Gesamtkomplikationsrate ohne Kenntnis der Armzuordnung zu analysieren.
   - Ziel der Analyse ist ausschließlich die Überprüfung der Annahmen, welche der Fallzahlkalkulation im Protokoll zugrunde liegen.
   - Bei N= 724 Patienten ist die Referenzbegutachtung der EKGs bereits erfolgt: Die Gesamtkomplikationsrate (Arme verblindet) mit Berücksichtigung der Referenzbegutachtung liegt bei 17.4%. Im RIPHeart Protokoll waren unter der Alternativhypothese nur 10% Gesamtkomplikationsrate der Fallzahlplanung zugrunde gelegt worden.
   - Zudem liegt die beobachtete Drop-out Rate mit 1.4% deutlich niedriger als die im Protokoll angenommene von 10%.

5. Eine Fallzahlanpassung aufgrund der beobachteten Gesamtrate nach einer verblindeten Zwischenanalyse beeinflusst nicht den Fehler I Art (vgl. Gould AL (1992), Statistics in Medicine 11,55-66).
   - Aufgrund der verblindeten Zwischenanalyse gehen wir konservativ davon aus, dass die Gesamtkomplikationsrate mehr als 12.5% betragen wird.
   - Zudem nehmen wir eine Drop-out rate von 2% statt 10% an.
   - Das Protokoll verlangt mindestens 80% Power mit einem zweiseitigen Test zum 5% Signifikanzniveau eine Reduktion der Komplikationsrate um 33% nachzuweisen.
   - Diese Powerforderung kann unter diesen neuen Annahmen bereits mit N=1400 erfüllen.
   - Sollte die Gesamtkomplikationsrate tatsächlich 17% betragen, so haben wir sogar eine Power von über 90%.

6. Die Rekrutierung der Studie kann daher ohne Verlust an Aussagekraft der Studie vorzeitig bei N=1400 beendet werden.

Auswirkungen und Risiken für Studienteilnehmer:

Die Änderung hat keine Auswirkungen auf die Studienteilnehmer und birgt keine Risiken.

Weiteres Vorgehen mit gemäß bisherigem Prüfplan behandelten Studienteilnehmer:

Studienteilnehmer, die zu diesem Zeitpunkt bereits gefragt worden sind, an der Studie teilzunehmen, können noch randomisiert werden. Für die Studienteilnehmer ergeben sich keine Änderungen.

Bewertungspflichtige Änderung:

Bewertungspflichtige Änderung nach GCP-V §10: wesentliche Veränderung Art der Leitung oder Durchführung der Studie wesentlich zu verändern.

Durch die Änderungen betroffenen Dokumente:

Keine weiteren Dokumente betroffen.

Weitere Änderungen

Ursprünglicher Text<br>(inkl. Angabe des Abschnitts im Prüfplan)	Änderung – neuer Text	Begründung und Auswirkungen
**Prüfplan-Synopse – Studienpopulation 4.2 Ausschlusskriterien**<br><br>Instabile Angina pectoris innerhalb der letzten 24 h präoperativ mit bereits präoperativen positiven Troponin T Anstieg	Instabile Angina pectoris innerhalb der letzten 24 h präoperativ mit bereits präoperativen Troponin T Anstieg ≥ 50 pg/ml	Nach der Protokollerstellung standen verbesserte Messverfahren zur Verfügung, wobei Troponin < 50 pg/ml klinisch nicht relevant ist.<br><br>Die Änderung präzisiert das Ausschlusskriterium und beinhaltet keine Risiken für potenzielle Studienteilnehmer.
**Verantwortliche Personen - Independent Data Monitoring and Safety Committee (IDMC)**<br><br>**Dipl. Inform. Jürgen Hedderich**<br>Institut für Medizinische Informatik und Statistik<br>Brunswiker Str. 10, 24105 Kiel<br>Telefon: 0431-597 3182; Fax: 0431-597 3193<br>Email: hedderich@medinfo.uni-kiel.de<br><br>**Prof. Dr. Georg Lutter**<br>Klinik für Herz- und Gefäßchirurgie<br>Universitätsklinikum Schleswig-Holstein, Campus Kiel<br>Arnold-Heller-Straße 3 (Haus 18), 24105 Kiel<br>Tel.: 0431-597 8036; Fax: 0431-597 4402<br>Email: georg.lutter@uk-sh.de	PD Dr. Andreas Zierer<br>Klinik für Thorax-, Herz- und Thorakale Gefäßchirurgie<br>Universitätsklinikum Frankfurt<br>Theodor-Stern-kai 7<br>60590 Frankfurt am Main<br>Email: andreas.zierer@kgu.de<br><br>Prof. Dr. Stephan Fichtlscherer<br>Medizinische Klinik III: Kardiologie, Angiologie, Nephrologie<br>Universitätsklinikum Frankfurt<br>Theodor-Stern-kai 7<br>60590 Frankfurt am Main<br>Email: stephan.fichtlscherer@kgu.de	Die Zusammensetzung des IDMC hat sich geändert. Die Änderung beinhaltet keine Risiken.

Ursprünglicher Text<br>(inkl. Angabe des Abschnitts im Prüfplan)	Änderung – neuer Text	Begründung und Auswirkungen
**Prof. Dr. Georg von Knobelsdorff**<br>St. Bernward Krankenhaus<br>Treibestr. 9, 31134 Hildesheim<br>Telefon: 05121-901425<br>Email: anaesthesie@bernward-khs.de		
**12.1 Endpunkt Komitee**<br><br>Das verblindete Endpunkt Komitee setzt sich aus Herr Professor Dr. med. Heribert Schunkert, Medizinische Klinik II – Kardiologie, Universitätsklinikum Schleswig-Holstein, Campus Lübeck und Herr Professor Dr. med. Norbert Frey, Medizinische Klinik III - Kardiologie, Universitätsklinikum Schleswig-Holstein, Campus Kiel zusammen.	Die verblindete Bewertung des Endpunktes „Myokardinfarkt" übernimmt das Herzzentrum Leipzig – Klinik für Innere Medizin / Kardiologie, vertreten durch Herrn Prof. Dr. med. Holger Thiele (bis 2013) und aktuell Herrn Dr. med. univ. Georg Fürnau.	Die Zusammensetzung des Endpunkt Komitees hat sich geändert. Die Änderung beinhaltet keine Risiken.
**Verantwortliche Personen - Endpunkt Komitee**<br><br>**Prof. Dr. med. Heribert Schunkert**<br>Medizinische Klinik II - Kardiologie<br>Universitätsklinikum Schleswig-Holstein, Campus Lübeck<br>Ratzeburger Allee 160, 23538 Lübeck<br>Tel.: 0451-500 2501; Fax: 0451-500 6437<br>Email: heribert.schunkert@uk-sh.de<br><br>**Prof. Dr. med. Norbert Frey**<br>Medizinische Klinik III - Kardiologie	**Prof. Dr. med. Holger Thiele/**<br>**Dr.med. univ. Georg Fürnau**<br>Universität Leipzig – Herzzentrum<br>Klinik für Innere Medizin/Kardiologie<br>Strümpellstr. 39<br>04289 Leipzig<br>Tel: 0341-865 1426/1428<br>Fax: 0341-865 1461<br>Email: georg.fuernau@medizin.uni-leipzig.de	

Ursprünglicher Text<br>(inkl. Angabe des Abschnitts im Prüfplan)	Änderung – neuer Text	Begründung und Auswirkungen
Universitätsklinikum Schleswig-Holstein, Campus Kiel<br>Arnold-Heller-Straße 3 (Haus 6), 24105 Kiel<br>Tel.: 0431-597 1440; Fax: 0431 597-1470<br>Email: norbert.frey@uk-sh.de	Prof Thiele: seit 1.12.2013 neue Adresse:<br><br>Medizinische Klinik II – Kardiologie, Angiologie, Intensivmedizin<br>Universitätsklinikum Schleswig-Holstein, Campus Lübeck<br>Ratzeburger Allee 160, 23538 Lübeck<br>Tel.: 0451-500 2501; Fax: 0451-500 6437<br>Email: holger.thiele@uksh.de	

2. Unterschriften zum Amendment

Bestätigung des Amendments

Das Amendment wird hiermit in seiner endgültigen Fassung bestätigt:

PD Dr. P. Meybohm<br>Studienleitung	Datum	Unterschrift
Prof. Dr. B. Bein<br>Studienleitung	Datum	Unterschrift
Prof. Dr. J. Cremer<br>Studienleitung	Datum	Unterschrift
Prof. Dr. Dr. K. Zacharowski<br>Studienleitung	Datum	Unterschrift
Dr. D. Hasenclever<br>Biometriker	Datum	Unterschrift

3. Anerkennung des Amendments

(vom Hauptprüfer des jeweiligen Zentrums zu unterzeichnen)

Hiermit bestätige ich, dass ich das vorliegende Amendment gelesen und verstanden habe und in allen Teilen anerkenne. Ich verpflichte mich, dafür zu sorgen, dass die von meinem Zentrum in die Studie eingebrachten Patienten nach den durch das Amendment geänderten Festlegungen des Prüfplans beobachtet und dokumentiert werden. Ich verpflichte mich, dafür Sorge zu tragen, dass alle an der klinischen Prüfung beteiligten Personen über den Inhalt des Amendments informiert sind.

Datum: ______________________________

Unterschrift Hauptprüfer: ______________________________

Adresse Prüfzentrum (Stempel): ______________________________

____

____

____

1. Study protocol - summary of changes

- Recruitment of new centers: The study was started at 10 university hospitals (please refer to study protocol version 1.0). To increase enrollment four additional university hospitals were subsequently recruited in 2011 (Charité-Universitätsmedizin Berlin, Campus Charité Mitte, Berlin; University Hospital Giessen, Giessen; Jena University Hospital, Jena; Medical Center of Johannes Gutenberg-University, Mainz)
- Sample size reduction to N=1,400 patients: Anticipating a pooled overall complication rate of 10% and assuming a 10% drop-out rate, we originally determined that the enrolment of 2,070 patients would be required to show a 33% risk reduction from 12% in the sham-RIPC group to 8% in the RIPC group using a two-sided Chi-square test with continuity correction with a significance level of 0.05 and providing a power of 80%. No formal interim analysis was planned. As patient accrual was lower than expected after the first 3 years, we performed a blinded sample size recalculation based on pooled data of the first 724 evaluable patients not affecting the type I error. We observed a higher than envisaged pooled complication rate (17.4% instead of 10%) and a much lower drop out rate (1.4% instead of 10%). Conservatively assuming a complication rate of 17% in the sham-RIPC arm and a drop out rate of 2% in the total study cohort, a total sample size of N=1,400 suffices to achieve 90% power to detect 33% risk reduction. After consent of the Independent Data Monitoring and Safety Committee and Steering Committee accrual stopped after 1,400 patients.
- Definition of an exclusion criteria 'instable angina' was more specified: 'instable angina pectoris (e.g. defined as elevated troponin T $\geq$ 0.05 µg/l up to 24 h)'
- New members of the Independent Data Monitoring and Safety Committee: Prof. A. Zierer, Prof. S. Fichtlscherer
- New members of the Clinical endpoint committee: Prof. H. Thiele, Dr. G. Fuernau

Statistical Analysis Plan
(SAP)

for the trial

Remote Ischaemic Preconditioning for Heart Surgery: a multi-centre randomized double-blinded controlled clinical trial
(RIPHeart)

NCT01067703

Coordinating Investigator

Prof. Dr. Patrick Meybohm

Klinik für Anästhesiologie, Intensivmedizin und Schmerztherapie

Klinikum der J.-W. von Goethe-Universität

Theodor-Stern-Kai 7, D - 60590 Frankfurt am Main

Responsible trial biometrician

Dr. Dirk Hasenclever

Institut für Medizinische Informatik, Statistik und Epidemiologie, Universität Leipzig

Härtelstrasse 16-18, D - 04107 Leipzig

dirk.hasenclever@imise.uni-leipzig.de

Date of the document: 08.12.2014

Version of the document: Final 1.0

We hereby approve the Statistical Analysis Plan in its final version:

Coordinating Investigator		
	Date	Signature
Biometrician		
	Date	Signature

Table of contents

1 Introduction 5

2 Changes in the planned analysis and sample size calculation 5

3 Endpoints and further variables 5

3.1 Primary Endpoint 5

3.1.1 Time period 6

3.1.2 Non-fatal myocardial infarction 6

3.1.3 Stroke 8

3.1.4 Acute renal failure 8

3.2 Secondary Endpoints 8

3.3 Covariates for confirmatory analyses 11

3.4 Further Variables 12

3.4.1 Baseline characteristics 12

3.4.2 Trial intervention 13

3.4.3 Intra- and postoperative assessments 13

4 General Analysis Definitions 16

4.1 Study periods 16

4.2 Analysis Populations 16

4.3 Major protocol violations 16

4.4 Subgroups 17

4.5 Handling of center effects 17

5 Planned analysis 18

5.1 Demographic and other baseline parameter 18

5.2 Concomitant Diseases and Medication 18

5.3 Compliance with regard to the Study Intervention 18

5.4 Pattern of Components of the primary endpoint 18

5.5 Primary endpoint 18

5.6 Secondary endpoints 19

5.6.1 Analysis of binary components of the composite endpoint 19

5.6.2 Analysis of time to composite endpoint and Overall survival 19

5.6.3 Analysis of time to occurrence of non-fatal endpoint components 19

5.6.4 Analysis of Troponin 20

5.6.5 Analysis of Creatinine 20

5.6.6 Analysis of durations....20
5.7 Further outcomes 21
5.8 Safety aspects 21
6 References 21
7 Appendix 22
7.1 Abbreviations....22

1 Introduction

The purpose of this document is to provide a detailed elaboration of the statistical analysis described in the protocol, including detailed procedures for the confirmatory analysis of the primary and secondary endpoints and other variables.

The Statistical Analysis Plan (SAP) assumes familiarity with the Study Protocol (Version final 0.1 dated 17.10.2010), including Protocol Amendments. The study design was also published in English (Meybohm et al.)[1]. If in doubt the study protocol formulation takes precedence.

The SAP is based on the planned analysis specification as written in the study protocol Section 7 “Biometrical Aspects of the Trial”. SAP readers may consult the study protocol for more background information on the study, e.g., on study objectives, study design and population, trial intervention, definition of measurements and variables, planning of sample size, and randomization.

IBM® SPSS ® Statistics Version 20 and R 3.1, (R packages named below) will be used for all statistical analyses.

2 Changes in the planned analysis and sample size calculation

Anticipating a pooled overall complication rate of 10% and assuming a 10% drop-out rate, we originally determined that the enrolment of 2,070 patients would be required to show a 33% risk reduction from 12% in the sham-RIPC group to 8% in the RIPC group using a two-sided Chi-square test with continuity correction with a significance level of 0.05 and providing a power of 80%. No formal interim analysis was planned. As patient accrual was lower than expected after the first 3 years, we performed a blinded sample size recalculation based on pooled data of the first 724 evaluable patients not affecting the type I error.11 We observed a higher than envisaged pooled complication rate (17.4% instead of 10%) and a much lower drop out rate (1.4% instead of 10%). Conservatively assuming a complication rate of 17% in the sham-RIPC arm and a drop out rate of 2% in the total study cohort, a total sample size of N=1,400 suffices to achieve 90% power to detect 33% risk reduction. After consent of the Independent Data Monitoring and Safety Committee and Steering Committee accrual stopped after 1,400 patients following ethical approval in May of 2014.

There were no changes to the planned analysis as outlined in the study protocol.

3 Endpoints and further variables

3.1 Primary Endpoint

The primary endpoint is defined as a composite of

- all-cause mortality,
- non-fatal myocardial infarction,
- any new stroke, and/or
- acute renal failure

until hospital discharge (with a maximum period of 14 days after surgery).

Operational definitions for the primary endpoint are detailed in the following sub-sections.

3.1.1 Time period

- We accepted documentation of the primary endpoint on the day before actual discharge.
    - Discharge typically takes place in the morning without further medical assessment on the day of discharge.
    - This rule should be uncritical since medical problems occurring on the day of planned discharge would prolong hospital stay.
- In case of prolonged hospital stay the primary endpoint was documented on day 14. A one day tolerance policy was applied.
- In cases in which a patient was discharged before day 14 from the primary hospital to a peripheral hospital, local investigators had to obtain written status information from the non-study center.
    - If not available take discharge from primary hospital.

3.1.2 Non-fatal myocardial infarction

Non-fatal myocardial infarction is defined (according to the ESC/ACC/AHA/WHF Task Force for the Redefinition of Myocardial Infarction) as

- Biomarker values more than five times the 99th percentile of the normal reference range

combined with

- new pathological Q-waves or
- new left bundle branch block (LBBB)
- acute signs of bypass ischemia using echocardiography or coronary angiography within the first 72 h,
- standard clinical criteria for myocardial infarction from 72 h on, or
- myocardial infarction diagnosed at autopsy.

Problems in biomarker criterion

- In the protocol only Troponin T is named as biomarker.
    - Later use of Troponin I was allowed, because some centers did not measure Troponin T routinely. The protocol was not changed respectively.
- After the start of the trial the biomarker requirement was raised to 10 fold by the task force (Thygesen et al.)[2]. As our trial was already ongoing, we kept the protocol definition of 5 fold.
- We did not require measurement of more specific biomarkers.
- Since practically all patients show a 10 fold Troponin increase after surgery the biomarker criterion is unspecific and non-informative.

Assessment of all available electrocardiograms by blinded endpoint committee

- There were two reference reader as named in the protocol amendment
- Assessment was based on criteria published by Thygesen et al.[3]

- All available ECGs of a patient were reviewed as a sequence by one reviewer.
    - ECGs were required at baseline, 24h and 72h
    - If additional ECGs were available these were also reviewed.
- The reference reader only documented presence of a Q-wave or a left bundle branch block for each individual ECG.
- The whole ECG sequence of the patient was resubmitted for a second review if in any ECG at any time point
    - a first review assessment differed from the assessment of the local investigator or
    - there was a positive reference finding and the local assessment was missing,
- If the first reference reader did not detect an abnormality and the local assessment was missing, no second reading was performed.
    - Complications are rare
        - The reference reading was generally more sensitive than the local assessment.
- The second reader
    - was aware that this was a discrepant case.
    - was not informed about what the discrepancy was.

The endpoint was determined based on the last reference assessment.

The algorithm to compute presence of a **new** abnormality has to deal

- with occasional transient abnormalities
- with rare missing or not evaluable ECGs
- with abnormalities already present at baseline.

A new abnormality counts for the composite primary endpoint

- if the abnormality is confirmed on the **last available** post surgery ECG
    - Transient abnormalities seen at 24h, but not at 72h are ignored.
    - If the 72h ECG is missing or not evaluable, the 24h ECG is used.
- **unless** the abnormality is positively confirmed to have been present at baseline.
    - In cases of missing or not evaluable baseline ECGs and no further information it is assumed that no abnormality was present at baseline.
    - Abnormalities at baseline were reported in 16% of the cases.

Handling of missing ECGs:

- There is a non negligible proportion of patients in whom either no electrocardiograms at all (1%) or only a baseline ECG, but no post surgery ECG (6%) was available, despite multiple queries and training by monitors.
    - In these cases the local assessment on the PE summary CRF is used.

3.1.3 Stroke

Stroke is defined in the protocol as

- any new, temporary or permanent, focal or global neurological deficit (NIH stroke score ≥4 points)
    - NIH stroke scale was documented preoperatively, at discharge from ICU and at hospital discharge or in case of suspected stroke.
- or evidence of stroke on autopsy

Problematic cases:

- In very few cases NIH score assessment was not feasible due to sedation, but there was radiological evidence of stroke. These cases are counted as stroke.
- NIH score ≥4 points was reported but explained by prolonged sedation, delirium or prolonged intubation. No stroke according to local investigator. We apply the operational protocol definition and count these cases as stroke.

3.1.4 Acute renal failure

Acute renal failure is defined as

- any serum creatinine greater than or equal to two-fold increase from baseline any time until hospital discharge or day 14, or
- urine output ≤0.5 mL/kg/h for 12 h any time until hospital discharge or day 14 (RIFLE injury), or
- use of renal replacement therapy, or
- evidence of renal failure on autopsy.

Note: There is an inconsistency between study protocol and design paper: The design paper reads “within the first 48 h”.

Problematic cases:

- Serum creatinine greater than or equal to two-fold increase from baseline confirmed, but local investigator does not agree with diagnosis of renal failure. We stick to the protocol: Counts as renal failure.

3.2 Secondary Endpoints

The **occurrence of any individual component of the composite** endpoint at 30 days and 3 and 12 months after surgery (phone interview).

- Dates of FU-interviews may vary about scheduled days. To circumvent problem with missing values we determine cumulative incidence curves using the actual Dates of FU-interviews and report rate estimates from these curves at d30, d90, d365.

The **cumulative duration of invasive ventilator support** (up to d30 post operation).

- CRFs allowed for documentation of multiple episodes of ventilator support with date and time of start and end. For the first episode start time is end of operation (= time of cutaneous suture).
- If a patient dies before first extubation, time of death is used as end of the initial mechanic ventilation episode.
- Time differences will be calculated and added and reported in [h].

The **cumulative duration of noninvasive ventilation** (up to d30 post operation).

- CRFs allowed for documentation of multiple episodes of ventilator support with date and time of start and end.
- Time differences will be calculated and added and reported in [h].
- Not mentioned in design paper

Length of stay on the intensive care unit (ICU)

- CRFs allowed for documentation of multiple episodes of stay on ICU with date and time of start and end. For the first episode start time is end of operation (= time of cutaneous suture).
- Time differences will be calculates and added and reported in [h].
- If a patient is transferred directly to an ICU at a peripheral hospital, ICU time there is taken into the account.
    - If a patient is transferred to a non-ICU at a peripheral hospital and later requires ICU care, this ICU time is not documented.
- If a patient dies on ICU, time of death is used as end of the respective ICU episode.

Total hospital stay

- This duration is calculated as the time difference between hospital discharge and end of operation (= time of cutaneous suture).
- If a patient is transferred to a peripheral hospital, hospitalization time there is added.
- If a patient dies in hospital, time of death is used as end of hospital stay.

Troponin T (preoperative, 6, 12, 24, and 48 h after surgery).

- Measurements are converted to [pg/ml] as preferred unit.

Troponin I (preoperative, 6, 12, 24, and 48 h after surgery).

- Since upper normal levels vary by center, measurements are converted to ratios to their upper normal level.

Renal function

Evaluation of renal function is based on serum creatinine measurements. Preoperative, 24h and 48h postoperative, and maximum creatinine values until discharge (up to day 14) are documented in the CRF.

- Measurements are converted to [mg/dl] as preferred unit.
- Mistake in design paper: 6h, 12h postoperative were not documented.

The following variables are analyzed:

- **Creatinine values preoperative, 24h and 48 postoperative** as well as **maximum creatinine**.

Vasopressor and inotropic support (yes / no)

- The cumulative dose of **vasopressor** (Noradrenalin, Vasopressin, Akrinor), **inotropic** (Adrenalin, Dobutamin) and **inodilatator** (Levosimendan, Enoximon, Milrinon) agents was documented for each drug used intraoperatively and within 24h postoperative.
- Six variables indicating use of the class of agents (vasopressor, inotrope, inodilatator) in the respective time period (intraoperative or postoperative) will be generated. Agent is counted as given if for at least one drug of the agent class in the time period the cumulative dose is documented to be > 0.

Note: there is a contradiction between the design paper (secondary endpoint "cumulative vasopressor and inotropic support within max. 7 days after surgery") and study protocol (secondary endpoints: "vasopressor and inotropic support intraoperative resp. within 24h postoperative").

New onset of atrial fibrillation within 4 days after surgery (yes / no)

- Atrial fibrillation is documented at 48h and 96h as yes / no
- Rhythm of the heart is documented with the categories "sinus rhythm / atrial fibrillation / other rhythm" at baseline, 24h and 72h in local ECG assessment. No reference reading has been performed.
- Preoperative atrial fibrillation was an exclusion criterion
- New onset of atrial fibrillation is defined as any atrial fibrillation reported post surgery unless documented to be present as baseline.

Incidence of **postoperative delirium** within 4 days after surgery (yes / no)

- Presence yes / no was documented (preoperative, 24, 48, 72, and 96 h after surgery).
- Assessment was to be based on the CAM-ICU score but details were not collected.
- Preoperative delirium was an exclusion criterion
- Postoperative delirium is counted as present if delirium was documented as "yes" at least once postoperatively.

Use of any **delirium medication** within 4 days after surgery (yes / no)

- On day 4, cumulative dose of delirium medication (Haloperidol, Promethazin, Lorazepam, Risperidon, Clonidin, other medication) was documented for each drug used.
- Delirium medication is counted as given if for at least one drug the cumulative dose is documented to be > 0.

Use of any **cardiac assist devices** intraoperative and postoperative within 30 days (yes / no)

- Intraoperative use of cardiac assist devices was documented on the CRF with the categories “none / intra-aortic balloon pump (IABP) / Extracorporeal Life Support System (ECLS) / other”. An indicator variable will be generated indicating any use of cardiac assist devices.
- Postoperative use of cardiac assist devices was documented on the CRF as IABP (yes/no), and if yes with start and end date, and accordingly for ECLS. Cardiac assist device is counted as present, if either IABP or ECLS was documented as “yes”.

Analysis of optional additional data (intraoperative transoesophageal echocardiography, neurocognitive testing) collected in subgroups only is not part of this analysis plan and will be specified later in an addendum.

3.3 Covariates for confirmatory analyses

According to the protocol “Logistic regression adjusting for EuroSCORE, diabetes mellitus status, concomitant treatment with statins, and incorporating centres as random effect will be used to estimate the treatment effect on the odds ratio scale with two-sided 95% confidence intervals provided.”

EuroSCORE was documented used to stratify randomization. The score includes a component type of operation.

- In some cases the planned type of operation differs from the type of surgery actually performed.
- Therefore we define an additional post operation EuroSCORE updating the EuroSCORE documented at randomization with the actual type of operation.
- The original EuroSCORE documented at randomization will be used in the intent to treat analysis.
- The actual EuroSCORE will be used in the per-protocol analysis.

Diabetes mellitus (yes / no / unknown) is recorded on baseline CRF.

- We crosschecked with reported use of anti diabetes drugs triggering queries in case of inconsistency.

Statins (yes / no): Is recorded on baseline CRF as cholesterol / lipid lowering drugs.

- We use cholesterol / lipid lowering drugs (yes / no) as a proxy for statin use although there are Fibrates which may have been used occasionally.
- We will use the label “Use of cholesterol lipid lowering drugs” in the publication.

3.4 Further Variables

3.4.1 Baseline characteristics

Age is recorded on patient registration CRF.

Gender is recorded on patient registration CRF.

Preexisting conditions

Ischemic heart disease (yes / no) is recorded on baseline CRF as “yes / no / unknown” (patient history). In addition, results of any cardiac catheter examination performed within 3 months prior to enrolment are documented on the baseline CRF.

- Ischemic heart disease is counted as present, if either documented as “yes” in the medical history or if its diagnosis is confirmed by cardiac catheter examination.
- “Unknown” is counted as missing.

Previous myocardial infarction (yes / no) is recorded on baseline CRF as “yes / no / unknown” (patient history). In addition, baseline ECG findings indicating prior myocardial ischemia are documented on the baseline CRF.

- Previous myocardial infarction is counted as present, if either documented as “yes” in the medical history or if it is diagnosed by baseline ECG.
- “Unknown” is counted as missing.

Chronic heart failure (yes / no) is recorded on baseline CRF as “yes / no / unknown” (patient history). “Unknown” is counted as missing.

Chronic obstructive pulmonary disease (yes / no) is recorded on baseline CRF as “yes / no / unknown” (patient history). “Unknown” is counted as missing.

Current smoking (yes / no)

Smoking is recorded on baseline CRF (patient history) with the categories “never / >= 6 months clean / current”. The categories “never” and “>= 6 months clean” are merged to “no”

Peripheral vascular disease (yes / no) is recorded on baseline CRF as “yes / no / unknown” (patient history). “Unknown” is counted as missing.

Chronic kidney disease (yes / no) is recorded on baseline CRF as “yes / no / unknown” (patient history). “Unknown” is counted as missing.

Previous stroke (yes / no) is recorded on baseline CRF as “yes / no / unknown” (patient history). “Unknown” is counted as missing.

Chronic arterial hypertension (yes / no) is recorded on baseline CRF as “yes / no / unknown” (patient history). “Unknown” is counted as missing.

Drug history

Cholesterol / lipid lowering drugs (yes / no) is recorded on baseline CRF (concomitant medication)

Beta-blocker (yes / no) is recorded on baseline CRF (concomitant medication)

ACE inhibitors (yes / no) is recorded on baseline CRF (concomitant medication)

Other

Left ventricular ejection fraction (LVEF) (grade 1: >=55% / grade 2: 30 – 55% / grade 3: < 30%)

- LVEF is assessed at baseline by either echocardiography or by cardiac catheter examination. For both procedures, it was allowed to document either LVEF in %, or LVEF function by categories only " >=55% / 45 – 55% / 30 – 45% / < 30%"
- The continuous values are graded. If no continuous values are available, the documented grades are used. The categories "45 – 55%" and "30 – 45%" are combined.
- If LVEF measurements by both methods are available the worst grade is used.

NYHA class (I / II / III / IV) is recorded on baseline CRF.

3.4.2 Trial intervention

The following variables will be used to assess compliance with the trial intervention (RIPC / sham RIPC)

Intervention done (yes/no) as recorded on the CRF.

Intervention as randomized (yes/no) as recorded on the CRF.

Applied pressure according to protocol (yes/no). The patient's blood pressure before start of intervention as well as the applied pressure for each intervention cycle is recorded on the CRF. The indicator variable is defined as "yes" if the applied pressure was protocol conform for all intervention cycles.

Number of intervention cycles. Each intervention cycle is documented with start and end time. The number of cycles for which a start time is recorded is calculated.

Total duration of intervention [min], defined as sum of time differences from start to end of all intervention cycles applied.

Blinding successful (yes/no) as recorded on the CRF.

3.4.3 Intra- and postoperative assessments

Type of surgery

Type of surgery was recorded on the CRF and will be classified as

- CABG (isolated)
- Aortic valve replacement (isolated)
- Mitral valve replacement or reconstruction (isolated)
- CABG + Aortic valve replacement
- Aorta ascendens replacement (isolated)
- Aortic valve replacement + Aorta ascendens replacement
- OPCAB
- Other

Time of procedure

On the CRF, start and end of cardiopulmonary bypass, cross clamping and circulatory arrest (if applicable) were documented, allowing for the documentation of more than one episodes.

Duration of the respective procedure is calculated as the time difference between start and end of the respective procedure, summing up duration of multiple episodes.

<u>Cardiopulmonary bypass management</u>

Type of **Priming**

The priming substances are documented (named) on the CRF.

Indicator variables will be generated for the following classes of priming substances

- Crystalloid
- Colloid (including hydroxyethyl starch 6%, gelatin)
- Albumin
- Mannitol

defined as "yes" if a substance belonging to the respective class is documented.

Type of **Cardioplegia** is documented on the CRF with the categories "Blood Buckberg / Bretschneider / Calafior / St. Thomas Plegie"

Cardioplegia Temperature is recorded on the CRF with the categories "warm (32 – 37°C) / cold (4 – 10°C).

Type of **Anesthesia**

The anesthetics used for anesthesia resp. for anesthesia maintenance are documented (named) on the CRF.

Indicator variables will be generated for the following classes of anesthetics

- Propofol / midazolam/ etomidat / (S)-ketamine / volatile agent
- Remifentanil / sufentanil

separately for anesthesia induction and maintenance and defined as "yes" if a substance belonging to the respective class is documented.

Lowest hematocrit value (%) intraoperatively and within 48h after surgery.

- The lowest hematocrit value is documented on the CRF for the intraoperative period
- Hematocrit values are documented at 24h and 48h postoperatively. The minimum of these two values will be used.

<u>Fluid intake</u>

Fluid intake is documented intraoperatively and for 24h as well as for 7 days postoperatively.

The following variables will be evaluated separately for the three time periods:

Crystalloid volume [ml] as recorded on the CRF.

Colloid volume [ml]. The CRF allowed for the documentation of several types of colloids. The total volume will be calculated.

Use of colloids (yes/no) defined as “yes”, if at least one colloid was documented as given.

Packed red blood cell transfusion volume [ml] as recorded on the CRF.

Transfusion with packed red blood cells (yes/no) is defined as “yes”, if the documented volume of packed red blood cells and / or the documented number of packed red blood cell packs is >0.

Transfusion with fresh frozen plasma (yes/no) is defined as “yes”, if the documented volume of fresh frozen plasma and / or the documented number of fresh frozen plasma packs is >0.

Transfusion with thrombocytes (yes/no) is defined as “yes”, if the documented volume of thrombocyte preparations and / or the documented number of thrombocyte preparation packs is >0.

<u>Complications</u>

The following complications are recorded during ICU stay

Wound infection (yes/no)

Severe sepsis / septic shock (yes/no)

Cardiopulmonary resuscitation (yes/no)

Cardiac arrest (yes/no)

Tracheotomy (yes/no)

Reoperation (yes/no)

- For reoperations, date, reason and type are recorded on the CRF.
- Reoperation is counted as “yes” if any reoperation has been documented.
- In addition, the number of reoperations is calculated.

Re-thoracotomy (yes/no)

The documented type of reoperation for all reoperations performed will be used to generate an indicator variable for re-thoracotomy. The indicator variable is defined as “yes”, if any of the re-operations was a re-thoracotomy.

4 General Analysis Definitions

4.1 Study periods

- The following study periods are distinguished for analysis
- Registration
- Baseline Assessment
- Randomization and Intervention (RIPC or shamRIPC)
- Intraoperative period (up to cutaneous suture)
- Postoperative period (up to 96 h postoperative)
- ICU stay (all time episodes on ICU up to 30 days postoperative)
- Hospital stay (until discharge from primary hospital)
- Assessment of primary endpoint (up to hospital discharge, but for a maximum period of 14 days after surgery)
- Follow-up at d30, d90, d365

4.2 Analysis Populations

Analysis populations are defined according to the protocol of the RIPHeart-Study (see protocol chapter 7.5).

The **Full Analysis Set (FAS)** for the intent to treat analysis consists of all randomized patients with valid informed consent in whom heart surgery was started. Since the primary endpoint is a complication rate of heart surgery patients without surgery are non-informative. The confirmatory analysis based on the FAS utilizes the randomized arm independent of the true intervention.

The **Per-Protocol Set (PPS)** is a subset of the FAS consisting of patients without major protocol violations (see below 4.3).

The **Safety Analysis Set** consists of all patients who received the intervention (RIPC/shamRIPC).

4.3 Major protocol violations

The following protocol violations are considered major:

- Violation of inclusion or exclusion criteria
- Intervention (RIPC/shamRIPC) not done or not performed according to randomization result.
- Off pump heart surgery performed.
- Volatile anesthetics (forbidden by protocol) used.

4.4 Subgroups

No subgroup analyses were pre-specified in the study protocol.

Exploratory subgroup analyses will be clearly labeled as post hoc.

4.5 Handling of center effects

Random center effects will be included in analytic models if feasible.

5 Planned analysis

A flowchart according to the CONSORT statement will describe the disposition of all patients registered to the trial detailing screening failure before randomization, withdrawals, drop-outs and inclusion in the analyses sets defined above. Respective listing will be provided. In addition, patients with major protocol violations will be listed.

Standard methods of descriptive statistics will be used always indicating the number of valid and missing values. Summary statistic will be reasonably rounded to avoid pseudo-precision.

General on the planned confirmatory analyses:

Each treatment comparison will be reported as a point estimate of the intervention effect on a meaningful scale, its 95% confidence interval and a respective p-value.

For each treatment comparison in the primary and secondary endpoints both a simple and a model based analysis will be provided.

- The simple, easy to communicate analyses will use widely known standard methods like the chi2-test or t-test and the associated confidence intervals for the underlying measure of difference.
- Advanced analyses will use generalized linear mixed models to adjust the treatment comparison for relevant covariates and possible random center effects.

If a relevant discordance between simple and advanced analysis arises, the model based approach is given preference in general; but the conflict has to be explored and the statistical report and any publication will mention and discuss the discrepancy.

5.1 Demographic and other baseline parameter

Demographic and other baseline parameter will be described for the whole FAS and by randomization arm.

5.2 Concomitant Diseases and Medication

Frequencies of concomitant diseases and medication will be described for the whole FAS and by randomization arm.

5.3 Compliance with regard to the Study Intervention

Patients will be listed on whom no intervention or an intervention not corresponding to the randomization arm was performed.

5.4 Pattern of Components of the primary endpoint

We will describe the frequencies of the observed patterns of components of the primary endpoint in cases with at least one complication. A Venn diagram will be used for illustration.

5.5 Primary endpoint

The study protocol specifies: Analysis of the binary composite primary endpoint is based on a logistic regression with treatment as fixed effect, adjusting for EuroSCORE, Diabetes status (Y/N) and concomitant use of Statin (y/n) (fixed effects) and Center (random effect). The

treatment difference is quantified as odds-ratio with 95% confidence interval. The test decision is based on the Wald test of the coefficient of treatment in the logistic regression.

Further specification of the primary analysis:

The EuroSCORE is entered as full score (as opposed to using the cut EuroSCORE 4+ as in stratification of the randomization) since the EuroSCORE was originally developed as linear predictor using logistic regression.

Statin use appeared as Cholesterol/Lipid lowering drugs (y/n) on the baseline CRF. This Variable is used.

The general linear mixed model is fitted using R package “lme4” with option: Gauss-Hermite Quadrature as integration method. For testing we used R package “lmerTest” using the Kenward & Roger method to determine the appropriate degrees of freedom, which is also used in SAS1. This choice of methodological details and software is in line with current published recommendations [4–7].

In addition, a simple estimate and 95% confidence interval for the difference in complication rates will be provided.

5.6 Secondary endpoints

5.6.1 Analysis of binary components of the composite endpoint

The same logistic regression model as for the primary endpoint is used in the analysis of the treatment effect in the separate binary components of the composite endpoint.

A table and a Forest plot of the confidence intervals of the intervention effect (both for model based odds ratios and crude differences in complication rates) for the primary composite endpoint and its binary components will be provided.

5.6.2 Analysis of time to composite endpoint and Overall survival

Time to event data will be described using Kaplan-Meier product limit estimator. The intervention effect will be analyzed with a Cox regression including the same covariates as in the analysis of the primary endpoint and including Center as random effect..

Validity of the proportional hazard assumption will be checked by graphical methods.

Impact of further covariates can be explored in supplementary analyses.

5.6.3 Analysis of time to occurrence of non-fatal endpoint components

Death from other causes is treated as a competing risk. Standard methods of Gray[9,10] for competing risk analysis as implemented in R package “cmprsk” will be used.

Cumulative incidence rates at d30, d90 and d365 will be estimated.

The intervention effect will be analyzed with the proportional subdistribution hazards regression model described in Fine and Gray[10] including the same covariates as in the analysis of the primary endpoint.

Impact of further covariates can be explored in supplementary analyses.

5.6.4 Analysis of Troponin

The analysis of the intervention effect on Troponin is complicated by the fact that most centers used Troponin T, but four centers used Troponin I instead of Troponin T, and one center switched between Troponin T and Troponin I for different patients.

In addition, upper normal values for Troponin T were uniform among centers, but varied for Troponin I.

Two separate analyses for Troponin T and Troponin I will be provided.

5.6.4.1 Analysis of Troponin T

Troponin T values will be analyzed on a log10 scale. Taking the logarithm transforms a heavily skewed distribution to an approximately symmetrical one.

The intervention effect on the maximum documented post surgery Troponin T value for each patient will be assessed with a t-test.

In addition the time course of Troponin T will be analyzed. In the protocol, Troponin measurements were scheduled at 6h, 12h, 24h, and 48h post operation. As exact times of the blood sampling were recorded these are used in the analysis specified below.

Blinded preparatory analysis revealed that Troponin T typically decreases from an early peak. This decrease is approximately linear if plotted against log(time after end of operation).

Thus we will fit a linear mixed model with a bivariate random patient effect on the intercept and the slope of the regression against log(time). After adjusting for the covariates specified for the analysis of the primary endpoint an intervention effect of the intercept and/or the slope will be assessed using the Wald test on the respective coefficient.

Impact of further covariates in particular type of surgery will be explored in supplementary analyses.

5.6.4.2 Analysis of Troponin I

Troponin I values will be analyzed in units of the respective center’s upper normal value and analyzed on the log10 scale.

The intervention effect on the maximum documented post operation Troponin I value for each patient will be assessed with a t-test.

5.6.5 Analysis of Creatinine

Serum creatinine values will be analyzed in units of the center’s upper normal value and analyzed on the log10 scale.

The maximum documented post surgery serum creatinine value for each patient will be assessed with a t-test.

5.6.6 Analysis of durations

- Cumulative duration of invasive ventilator support
- Cumulative duration of non-invasive ventilator support

- Length of stay on the intensive care unit
- Total hospital stay

In these endpoints for simple analysis the t-test is used. For model based analysis a linear mixed model analogous to the analysis of the primary endpoint is used.

5.7 Further outcomes

Further endpoints (presence of a condition post surgery) are binary. Analysis of the treatment effect in further binary variables follows the analysis strategy for the primary endpoint.

5.8 Safety aspects

Adverse Event (AE) and Serious Adverse Event (SAE) reporting was limited by the protocol (6.1.3) to a time horizon of 96h post operation and exclusively to AEs that concerned the patient's arm on which the study intervention (RIPC/ShamRIPC) was performed or AEs otherwise suspected to be related to the RIPC/ShamRIPC intervention.

Few AEs were expected and reported.

Adverse events and serious adverse events will be listed by study arm.

6 References

1. Meybohm P, Zacharowski K, Cremer J, et al. Remote ischaemic preconditioning for heart surgery. The study design for a multi-center randomized double-blinded controlled clinical trial--the RIPHeart-Study. Eur Heart J. 2012 Jun;33(12):1423-6.
2. Thygesen K, Alpert JS, Jaffe AS, et al. Third universal definition of myocardial infarction. Eur Heart J. 2012 Oct;33(20):2551-67.
3. Thygesen K, Alpert JS, White HD, et al. Universal definition of myocardial infarction. Eur Heart J. 2007 Oct;28(20):2525-38.
4. Schaalje GB, McBride JB, Fellingham GW. Adequacy of approximations to distributions of test statistics in complex mixed linear models. JABES 2002;7:512–24.
5. Bolker BM, Brooks ME, Clark CJ, et al. Generalized linear mixed models: a practical guide for ecology and evolution. Trends in ecology & evolution 2009;24:127–35. eng.
6. Casals M, Girabent-Farrés M, Carrasco JL. Methodological quality and reporting of generalized linear mixed models in clinical medicine (2000-2012): a systematic review. PloS one 2014;9:e112653. eng.
7. Li B, Lingsma HF, Steyerberg EW, Lesaffre E. Logistic random effects regression models: a comparison of statistical packages for binary and ordinal outcomes. BMC medical research methodology 2011;11:77. eng.
8. Thiele J. Potential of GLMM in modelling invasive spread. CAB Reviews 2012;7.
9. Gray RJ. A Class of K-Sample Tests for Comparing the Cumulative Incidence of a Competing Risk. The Annals of Statistics 1988;16:1141–54.
10. Fine JP, Gray RJ. A Proportional Hazards Model for the Subdistribution of a Competing Risk. Journal of the American Statistical Association 1999;94:496–509.
11. Gould AL. Interim analyses for monitoring clinical trials that do not materially affect the type I error rate. Stat Med 1992;11:55-66.

7 Appendix

7.1 Abbreviations

AE	Adverse event
CABG	Coronary artery bypass graft
CPB	Cardiopulmonary bypass
CRF	Case Report Form
EC	Ethics Committee
ECG	Electrocardiogram
ECLS	Extracorporeal Life Support System
FAS	Full analysis set
GCP	Good Clinical Practice
ICH	International Conference on Harmonisation
IABP	Intra-aortic balloon pump
LVEF	Left ventricular ejection fraction
OPCAB	Off-Pump-Coronary-Artery-Bypass
PPS	Per protocol set
RIPC	Remote ischemic pre-conditioning
SAE	Serious adverse event
SAP	Statistical Analysis Plan
SAS	Safety analysis set

Statistical Analysis Plan
(SAP)

for the trial

Remote Ischaemic Preconditioning for Heart Surgery: a multi-centre randomized double-blinded controlled clinical trial
(RIPHeart)

NCT01067703

Coordinating Investigator

Prof. Dr. Patrick Meybohm

Klinik für Anästhesiologie, Intensivmedizin und Schmerztherapie

Klinikum der J.-W. von Goethe-Universität

Theodor-Stern-Kai 7, D - 60590 Frankfurt am Main

Responsible trial biometrician

Dr. Dirk Hasenclever

Institut für Medizinische Informatik, Statistik und Epidemiologie, Universität Leipzig

Härtelstrasse 16-18, D - 04107 Leipzig

dirk.hasenclever@imise.uni-leipzig.de

Date of the document: 08.01.2015

Version of the document: Final 2.0

We hereby approve the Statistical Analysis Plan in its final version:

Coordinating Investigator	Date	Signature
Biometrician	Date	Signature

Table of contents

1 Introduction 5

2 Changes in the planned analysis and sample size calculation 5

2.1 Changes with respect to study protocol 5

2.2 Changes with respect to the Statistical Analysis Plan Version 1.0 5

3 Endpoints and further variables 6

3.1 Primary Endpoint 6

3.1.1 Time period 6

3.1.2 Non-fatal myocardial infarction 6

3.1.3 Stroke 8

3.1.4 Acute renal failure 8

3.2 Secondary Endpoints 9

3.3 Covariates for confirmatory analyses 11

3.4 Further Variables 12

3.4.1 Baseline characteristics 12

3.4.2 Trial intervention 13

3.4.3 Intra- and postoperative assessments 14

4 General Analysis Definitions 16

4.1 Study periods 16

4.2 Analysis Populations 16

4.3 Major protocol violations 17

4.3.1 Violation of inclusion or exclusion criteria 17

4.3.2 Intervention not per protocol 17

4.3.3 Surgery performed meeting an exclusion criterion 18

4.4 Subgroups 18

4.5 Handling of center effects 18

5 Planned analysis 19

5.1 Demographic and other baseline parameter 19

5.2 Concomitant Diseases and Medication 19

5.3 Compliance with regard to the Study Intervention 19

5.4 Pattern of Components of the primary endpoint 19

5.5 Primary endpoint 19

5.6 Secondary endpoints 20

5.6.1 Analysis of binary components of the composite endpoint 20
5.6.2 Analysis of time to composite endpoint and Overall survival 20
5.6.3 Analysis of time to occurrence of non-fatal endpoint components 20
5.6.4 Analysis of Troponin 21
5.6.5 Analysis of Creatinine 21
5.6.6 Analysis of durations 21
5.7 Further outcomes 22
5.8 Sub-group analyses 22
5.9 Safety aspects 22
6 References 22
7 Appendix 23
7.1 Abbreviations 23

1 Introduction

The purpose of this document is to provide a detailed elaboration of the statistical analysis described in the protocol, including detailed procedures for the confirmatory analysis of the primary and secondary endpoints and other variables.

The Statistical Analysis Plan (SAP) assumes familiarity with the Study Protocol (Version final 0.1 dated 17.10.2010), including Protocol Amendments. The study design was also published in English (Meybohm et al.)[1]. If in doubt the study protocol formulation takes precedence.

The SAP is based on the planned analysis specification as written in the study protocol Section 7 "Biometrical Aspects of the Trial". SAP readers may consult the study protocol for more background information on the study, e.g., on study objectives, study design and population, trial intervention, definition of measurements and variables, planning of sample size, and randomization.

IBM® SPSS ® Statistics Version 20 and R 3.1, (R packages named below) will be used for all statistical analyses.

2 Changes in the planned analysis and sample size calculation

2.1 Changes with respect to study protocol

Anticipating a pooled overall complication rate of 10% and assuming a 10% drop-out rate, we originally determined that the enrolment of 2,070 patients would be required to show a 33% risk reduction from 12% in the sham-RIPC group to 8% in the RIPC group using a two-sided Chi-square test with continuity correction with a significance level of 0.05 and providing a power of 80%. No formal interim analysis was planned. As patient accrual was lower than expected after the first 3 years, we performed a blinded sample size recalculation based on pooled data of the first 724 evaluable patients not affecting the type I error.11 We observed a higher than envisaged pooled complication rate (17.4% instead of 10%) and a much lower drop out rate (1.4% instead of 10%). Conservatively assuming a complication rate of 17% in the sham-RIPC arm and a drop out rate of 2% in the total study cohort, a total sample size of N=1,400 suffices to achieve 90% power to detect 33% risk reduction. After consent of the Independent Data Monitoring and Safety Committee and Steering Committee accrual stopped after 1,400 patients following ethical approval in May of 2014.

2.2 Changes with respect to the Statistical Analysis Plan Version 1.0

In section 3.4 (Further Variables) a few derived variables were added (Aortic aneurysm, Mitral regurgitation, Aortic insufficiency, Aortic valve stenosis, Total duration of intervention as per protocol) or their classification was modified (Applied pressure according to protocol, Type of surgery).

In section 4.3 an operational definition of major protocol violations was added.

For consistency, in 5.6.4 and 5.6.5 analyses of maximum post-surgery troponin and creatinine values are based on linear mixed models adjusting for the same covariates including Center as random effect as in the analysis of the primary endpoint.

A section 5.8 on explorative sub-group analyses was added.

3 Endpoints and further variables

3.1 Primary Endpoint

The primary endpoint is defined as a composite of

- all-cause mortality,
- non-fatal myocardial infarction,
- any new stroke, and/or
- acute renal failure

until hospital discharge (with a maximum period of 14 days after surgery).

Operational definitions for the primary endpoint are detailed in the following sub-sections.

3.1.1 Time period

- We accepted documentation of the primary endpoint on the day before actual discharge.
  - Discharge typically takes place in the morning without further medical assessment on the day of discharge.
  - This rule should be uncritical since medical problems occurring on the day of planned discharge would prolong hospital stay.
- In case of prolonged hospital stay the primary endpoint was documented on day 14. A one day tolerance policy was applied.
- In cases in which a patient was discharged before day 14 from the primary hospital to a peripheral hospital, local investigators had to obtain written status information from the non-study center.
  - If not available take discharge from primary hospital.

3.1.2 Non-fatal myocardial infarction

Non-fatal myocardial infarction is defined (according to the ESC/ACC/AHA/WHF Task Force for the Redefinition of Myocardial Infarction) as

- Biomarker values more than five times the 99th percentile of the normal reference range

combined with

- new pathological Q-waves or
- new left bundle branch block (LBBB)
- acute signs of bypass ischemia using echocardiography or coronary angiography within the first 72 h,
- standard clinical criteria for myocardial infarction from 72 h on, or
- myocardial infarction diagnosed at autopsy.

Problems in biomarker criterion

- In the protocol only Troponin T is named as biomarker.
  - Later use of Troponin I was allowed, because some centers did not measure Troponin T routinely. The protocol was not changed respectively.
- After the start of the trial the biomarker requirement was raised to 10 fold by the task force (Thygesen et al.)[2]. As our trial was already ongoing, we kept the protocol definition of 5 fold.
- We did not require measurement of more specific biomarkers.
- Since practically all patients show a 10 fold Troponin increase after surgery the biomarker criterion is unspecific and non-informative.

Assessment of all available electrocardiograms by blinded endpoint committee

- There were two reference reader as named in the protocol amendment
- Assessment was based on criteria published by Thygesen et al.[3]
- All available ECGs of a patient were reviewed as a sequence by one reviewer.
  - ECGs were required at baseline, 24h and 72h
  - If additional ECGs were available these were also reviewed.
- The reference reader only documented presence of a Q-wave or a left bundle branch block for each individual ECG.
- The whole ECG sequence of the patient was resubmitted for a second review if in any ECG at any time point
  - a first review assessment differed from the assessment of the local investigator or
  - there was a positive reference finding and the local assessment was missing,
- If the first reference reader did not detect an abnormality and the local assessment was missing, no second reading was performed.
  - Complications are rare
    - The reference reading was generally more sensitive than the local assessment.
- The second reader
  - was aware that this was a discrepant case.
  - was not informed about what the discrepancy was.

The endpoint was determined based on the last reference assessment.

The algorithm to compute presence of a **new** abnormality has to deal

- with occasional transient abnormalities
- with rare missing or not evaluable ECGs
- with abnormalities already present at baseline.

A new abnormality counts for the composite primary endpoint

- if the abnormality is confirmed on the **last available** post surgery ECG
  - Transient abnormalities seen at 24h, but not at 72h are ignored.
  - If the 72h ECG is missing or not evaluable, the 24h ECG is used.
- **unless** the abnormality is positively confirmed to have been present at baseline.
  - In cases of missing or not evaluable baseline ECGs and no further information it is assumed that no abnormality was present at baseline.
  - Abnormalities at baseline were reported in 16% of the cases.

Handling of missing ECGs:

- There is a non negligible proportion of patients in whom either no electrocardiograms at all (1%) or only a baseline ECG, but no post surgery ECG (6%) was available, despite multiple queries and training by monitors.
  - In these cases the local assessment on the PE summary CRF is used.

3.1.3 Stroke

Stroke is defined in the protocol as

- any new, temporary or permanent, focal or global neurological deficit (NIH stroke score ≥4 points)
  - NIH stroke scale was documented preoperatively, at discharge from ICU and at hospital discharge or in case of suspected stroke.
- or evidence of stroke on autopsy

Problematic cases:

- In very few cases NIH score assessment was not feasible due to sedation, but there was radiological evidence of stroke. These cases are counted as stroke.
- NIH score ≥4 points was reported but explained by prolonged sedation, delirium or prolonged intubation. No stroke according to local investigator. We apply the operational protocol definition and count these cases as stroke.

3.1.4 Acute renal failure

Acute renal failure is defined as

- any serum creatinine greater than or equal to two-fold increase from baseline any time until hospital discharge or day 14, or
- urine output ≤0.5 mL/kg/h for 12 h any time until hospital discharge or day 14 (RIFLE injury), or
- use of renal replacement therapy, or
- evidence of renal failure on autopsy.

Note: There is an inconsistency between study protocol and design paper: The design paper reads “within the first 48 h”.

Problematic cases:

- Serum creatinine greater than or equal to two-fold increase from baseline confirmed, but local investigator does not agree with diagnosis of renal failure. We stick to the protocol: Counts as renal failure.

3.2 Secondary Endpoints

The **occurrence of any individual component of the composite** endpoint at 30 days and 3 and 12 months after surgery (phone interview).

- Dates of FU-interviews may vary about scheduled days. To circumvent problem with missing values we determine cumulative incidence curves using the actual Dates of FU-interviews and report rate estimates from these curves at d30, d90, d365.

The **cumulative duration of invasive ventilator support** (up to d30 post operation).

- CRFs allowed for documentation of multiple episodes of ventilator support with date and time of start and end. For the first episode start time is end of operation (= time of cutaneous suture).
- If a patient dies before first extubation, time of death is used as end of the initial mechanic ventilation episode.
- Time differences will be calculated and added and reported in [h].

The **cumulative duration of noninvasive ventilation** (up to d30 post operation).

- CRFs allowed for documentation of multiple episodes of ventilator support with date and time of start and end.
- Time differences will be calculated and added and reported in [h].
- Not mentioned in design paper

Length of stay on the intensive care unit (ICU)

- CRFs allowed for documentation of multiple episodes of stay on ICU with date and time of start and end. For the first episode start time is end of operation (= time of cutaneous suture).
- Time differences will be calculates and added and reported in [h].
- If a patient is transferred directly to an ICU at a peripheral hospital, ICU time there is taken into the account.
    - If a patient is transferred to a non-ICU at a peripheral hospital and later requires ICU care, this ICU time is not documented.
- If a patient dies on ICU, time of death is used as end of the respective ICU episode.

Total hospital stay

- This duration is calculated as the time difference between hospital discharge and end of operation (= time of cutaneous suture).
- If a patient is transferred to a peripheral hospital, hospitalization time there is added.

- If a patient dies in hospital, time of death is used as end of hospital stay.

Troponin T (preoperative, 6, 12, 24, and 48 h after surgery).

- Measurements are converted to [pg/ml] as preferred unit.

Troponin I (preoperative, 6, 12, 24, and 48 h after surgery).

- Since upper normal levels vary by center, measurements are converted to ratios to their upper normal level.

Renal function

Evaluation of renal function is based on serum creatinine measurements. Preoperative, 24h and 48h postoperative, and maximum creatinine values until discharge (up to day 14) are documented in the CRF.

- Measurements are converted to [mg/dl] as preferred unit.
- Mistake in design paper: 6h, 12h postoperative were not documented.

The following variables are analyzed:

- **Creatinine values preoperative, 24h and 48 postoperative** as well as **maximum creatinine**.

Vasopressor and inotropic support (yes / no)

- The cumulative dose of **vasopressor** (Noradrenalin, Vasopressin, Akrinor), **inotropic** (Adrenalin, Dobutamin) and **inodilatator** (Levosimendan, Enoximon, Milrinon) agents was documented for each drug used intraoperatively and within 24h postoperative.
- Six variables indicating use of the class of agents (vasopressor, inotrope, inodilatator) in the respective time period (intraoperative or postoperative) will be generated. Agent is counted as given if for at least one drug of the agent class in the time period the cumulative dose is documented to be > 0.

Note: there is a contradiction between the design paper (secondary endpoint "cumulative vasopressor and inotropic support within max. 7 days after surgery") and study protocol (secondary endpoints: "vasopressor and inotropic support intraoperative resp. within 24h postoperative").

New onset of atrial fibrillation within 4 days after surgery (yes / no)

- Atrial fibrillation is documented at 48h and 96h as yes / no
- Rhythm of the heart is documented with the categories "sinus rhythm / atrial fibrillation / other rhythm" at baseline, 24h and 72h in local ECG assessment. No reference reading has been performed.
- Preoperative atrial fibrillation was an exclusion criterion
- New onset of atrial fibrillation is defined as any atrial fibrillation reported post surgery unless documented to be present as baseline.

Incidence of **postoperative delirium** within 4 days after surgery (yes / no)

- Presence yes / no was documented (preoperative, 24, 48, 72, and 96 h after surgery).
- Assessment was to be based on the CAM-ICU score but details were not collected.
- Preoperative delirium was an exclusion criterion
- Postoperative delirium is counted as present if delirium was documented as “yes” at least once postoperatively.

Use of any **delirium medication** within 4 days after surgery (yes / no)

- On day 4, cumulative dose of delirium medication (Haloperidol, Promethazin, Lorazepam, Risperidon, Clonidin, other medication) was documented for each drug used.
- Delirium medication is counted as given if for at least one drug the cumulative dose is documented to be > 0.

Use of any **cardiac assist devices** intraoperative and postoperative within 30 days (yes / no)

- Intraoperative use of cardiac assist devices was documented on the CRF with the categories “none / intra-aortic balloon pump (IABP) / Extracorporeal Life Support System (ECLS) / other”. An indicator variable will be generated indicating any use of cardiac assist devices.
- Postoperative use of cardiac assist devices was documented on the CRF as IABP (yes/no), and if yes with start and end date, and accordingly for ECLS. Cardiac assist device is counted as present, if either IABP or ECLS was documented as “yes”.

Analysis of optional additional data (intraoperative transoesophageal echocardiography, neurocognitive testing) collected in subgroups only is not part of this analysis plan and will be specified later in an addendum.

3.3 Covariates for confirmatory analyses

According to the protocol “Logistic regression adjusting for EuroSCORE, diabetes mellitus status, concomitant treatment with statins, and incorporating centres as random effect will be used to estimate the treatment effect on the odds ratio scale with two-sided 95% confidence intervals provided.”

EuroSCORE was documented used to stratify randomization. The score includes a component type of operation.

- In some cases the planned type of operation differs from the type of surgery actually performed.
- Therefore we define an additional post operation EuroSCORE updating the EuroSCORE documented at randomization with the actual type of operation.
- The original EuroSCORE documented at randomization will be used in the intent to treat analysis.

- The actual EuroSCORE will be used in the per-protocol analysis.

Diabetes mellitus (yes / no / unknown) is recorded on baseline CRF.

- We crosschecked with reported use of anti diabetes drugs triggering queries in case of inconsistency.

Statins (yes / no): Is recorded on baseline CRF as cholesterol / lipid lowering drugs.

- We use cholesterol / lipid lowering drugs (yes / no) as a proxy for statin use although there are Fibrates which may have been used occasionally.
- We will use the label “Use of cholesterol lipid lowering drugs” in the publication.

3.4 Further Variables

3.4.1 Baseline characteristics

Age is recorded on patient registration CRF.

Gender is recorded on patient registration CRF.

Preexisting conditions

Ischemic heart disease (yes / no) is recorded on baseline CRF as “yes / no / unknown” (patient history). In addition, results of any cardiac catheter examination performed within 3 months prior to enrolment are documented on the baseline CRF.

- Ischemic heart disease is counted as present, if either documented as “yes” in the medical history or if its diagnosis is confirmed by cardiac catheter examination.
- “Unknown” is counted as missing.

Previous myocardial infarction (yes / no) is recorded on baseline CRF as “yes / no / unknown” (patient history). In addition, baseline ECG findings indicating prior myocardial ischemia are documented on the baseline CRF.

- Previous myocardial infarction is counted as present, if either documented as “yes” in the medical history or if it is diagnosed by baseline ECG.
- “Unknown” is counted as missing.

Chronic heart failure (yes / no) is recorded on baseline CRF as “yes / no / unknown” (patient history). “Unknown” is counted as missing.

Aortic aneurysm (yes / no) is recorded on baseline CRF as “yes / no / unknown” (patient history). In addition, during baseline echocardiography presence of an aortic aneurysm was assessed.

- Aortic aneurysm is counted as present, if either
- documented as “yes” in the echocardiographic assessment, or
- documented as “yes” in the medical history in cases with missing echocardiographic results, or
- if it is documented as “yes” in the medical history and confirmed by further plain text explanations, even if it was not diagnosed by baseline ECG.
- “Unknown” is counted as missing.

Mitral regurgitation, Aortic insufficiency and **Aortic valve stenosis** were assessed by baseline echocardiography and documented on the baseline CRF as "none / mild / moderate / severe"

Chronic obstructive pulmonary disease (yes / no) is recorded on baseline CRF as "yes / no / unknown" (patient history). "Unknown" is counted as missing.

Current smoking (yes / no)

Smoking is recorded on baseline CRF (patient history) with the categories "never / >= 6 months clean / current". The categories "never" and ">= 6 months clean" are merged to "no"

Peripheral vascular disease (yes / no) is recorded on baseline CRF as "yes / no / unknown" (patient history). "Unknown" is counted as missing.

Chronic kidney disease (yes / no) is recorded on baseline CRF as "yes / no / unknown" (patient history). "Unknown" is counted as missing.

Previous stroke (yes / no) is recorded on baseline CRF as "yes / no / unknown" (patient history). "Unknown" is counted as missing.

Chronic arterial hypertension (yes / no) is recorded on baseline CRF as "yes / no / unknown" (patient history). "Unknown" is counted as missing.

Drug history

Cholesterol / lipid lowering drugs (yes / no) is recorded on baseline CRF (concomitant medication)

Beta-blocker (yes / no) is recorded on baseline CRF (concomitant medication)

ACE inhibitors (yes / no) is recorded on baseline CRF (concomitant medication)

Other

Left ventricular ejection fraction (LVEF) (grade 1: >=55% / grade 2: 30 – 55% / grade 3: < 30%)

- LVEF is assessed at baseline by either echocardiography or by cardiac catheter examination. For both procedures, it was allowed to document either LVEF in %, or LVEF function by categories only " >=55% / 45 – 55% / 30 – 45% / < 30%"
- The continuous values are graded. If no continuous values are available, the documented grades are used. The categories "45 – 55%" and "30 – 45%" are combined.
- If LVEF measurements by both methods are available the worst grade is used.

NYHA class (I / II / III / IV) is recorded on baseline CRF.

3.4.2 Trial intervention

The following variables will be used to assess compliance with the trial intervention (RIPC / sham RIPC)

Intervention done (yes/no) as recorded on the CRF.

Intervention as randomized (yes/no) as recorded on the CRF.

Applied pressure according to protocol (yes / too low / too high). The patient's blood pressure before start of intervention as well as the applied pressure for each intervention cycle is recorded on the CRF. The indicator variable is defined as "yes" if the applied

pressure was protocol conform for all intervention cycles, and as “too low” resp. “too high” if the applied pressure was lower or higher as per protocol. No deviations were tolerated.

Number of intervention cycles. Each intervention cycle is documented with start and end time. The number of cycles for which a start time is recorded is calculated.

Total duration of intervention [min], defined as sum of time differences from start to end of all intervention cycles applied.

Total duration of intervention as per protocol (yes/no). As per protocol, every inflation cycles and every pause should have had a duration of 5 min each. We tolerate a deviation of ± 2min per cycle or pause. The indicator variable “Total duration of intervention as per protocol” is defined as “no”, if in at least one inflation cycle or pause the duration is outside the tolerated interval.

Blinding successful (yes/no) as recorded on the CRF.

3.4.3 Intra- and postoperative assessments

Type of surgery

Type of surgery was recorded on the CRF and will be classified as

- CABG (isolated)
- Aortic valve replacement (isolated)
- Mitral valve replacement or reconstruction (isolated)
- Aorta ascendens replacement (isolated)
- Combined procedures
- Other type of surgery (alone)

Time of procedure

On the CRF, start and end of cardiopulmonary bypass, cross clamping and circulatory arrest (if applicable) were documented, allowing for the documentation of more than one episodes.

Duration of the respective procedure is calculated as the time difference between start and end of the respective procedure, summing up duration of multiple episodes.

Cardiopulmonary bypass management

Type of **Priming**

The priming substances are documented (named) on the CRF.

Indicator variables will be generated for the following classes of priming substances

- Crystalloid
- Colloid (including hydroxyethyl starch 6%, gelatin)
- Albumin
- Mannitol

defined as “yes” if a substance belonging to the respective class is documented.

Type of **Cardioplegia** is documented on the CRF with the categories “Blood Buckberg / Bretschneider / Calafior / St. Thomas’ solution”

Cardioplegia Temperature is recorded on the CRF with the categories “warm (32 – 37°C) / cold (4 – 10°C).

Type of **Anesthesia**

The anesthetics used for anesthesia resp. for anesthesia maintenance are documented (named) on the CRF.

Indicator variables will be generated for the following classes of anesthetics

- Propofol / midazolam/ etomidat / (S)-ketamine / volatile agent
- Remifentanil / sufentanil

separately for anesthesia induction and maintenance and defined as “yes” if a substance belonging to the respective class is documented.

Lowest hematocrit value (%) intraoperatively and within 48h after surgery.

- The lowest hematocrit value is documented on the CRF for the intraoperative period
- Hematocrit values are documented at 24h and 48h postoperatively. The minimum of these two values will be used.

Fluid intake

Fluid intake is documented intraoperatively and for 24h as well as for 7 days postoperatively.

The following variables will be evaluated separately for the three time periods:

Crystalloid volume [ml] as recorded on the CRF.

Colloid volume [ml]. The CRF allowed for the documentation of several types of colloids. The total volume will be calculated.

Use of colloids (yes/no) defined as “yes”, if at least one colloid was documented as given.

Packed red blood cell transfusion volume [ml] as recorded on the CRF.

Transfusion with packed red blood cells (yes/no) is defined as “yes”, if the documented volume of packed red blood cells and / or the documented number of packed red blood cell packs is >0.

Transfusion with fresh frozen plasma (yes/no) is defined as “yes”, if the documented volume of fresh frozen plasma and / or the documented number of fresh frozen plasma packs is >0.

Transfusion with thrombocytes (yes/no) is defined as “yes”, if the documented volume of thrombocyte preparations and / or the documented number of thrombocyte preparation packs is >0.

Complications

The following complications are recorded during ICU stay

Wound infection (yes/no)

Severe sepsis / septic shock (yes/no)

Cardiopulmonary resuscitation (yes/no)

Cardiac arrest (yes/no)

Tracheotomy (yes/no)

Reoperation (yes/no)

- For reoperations, date, reason and type are recorded on the CRF.
- Reoperation is counted as “yes” if any reoperation has been documented.
- In addition, the number of reoperations is calculated.

Re-thoracotomy (yes/no)

The documented type of reoperation for all reoperations performed will be used to generate an indicator variable for re-thoracotomy. The indicator variable is defined as “yes”, if any of the re-operations was a re-thoracotomy.

4 General Analysis Definitions

4.1 Study periods

The following study periods are distinguished for analysis:

- Registration
- Baseline Assessment
- Randomization and Intervention (RIPC or shamRIPC)
- Intraoperative period (up to cutaneous suture)
- Postoperative period (up to 96 h postoperative)
- ICU stay (all time episodes on ICU up to 30 days postoperative)
- Hospital stay (until discharge from primary hospital)
- Assessment of primary endpoint (up to hospital discharge, but for a maximum period of 14 days after surgery)
- Follow-up at d30, d90, d365

4.2 Analysis Populations

Analysis populations are defined according to the protocol of the RIPHeart-Study (see protocol chapter 7.5).

The **Full Analysis Set (FAS)** for the intent to treat analysis consists of all randomized patients with valid informed consent in whom heart surgery was started. Since the primary endpoint is a complication rate of heart surgery patients without surgery are non-informative. The confirmatory analysis based on the FAS utilizes the randomized arm independent of the true intervention.

The **Per-Protocol Set (PPS)** is a subset of the FAS consisting of patients without major protocol violations (see below 4.3).

The **Safety Analysis Set** consists of all patients who received the intervention (RIPC/shamRIPC).

4.3 Major protocol violations

The following protocol violations are considered major:

- Violation of inclusion or exclusion criteria
- Intervention (RIPC/shamRIPC) not done or not performed according to randomization result or not performed as per protocol.
- Surgery performed meeting an exclusion criterion
- Volatile anesthetics (forbidden by protocol) used (as recorded on the CRF).

4.3.1 Violation of inclusion or exclusion criteria

Inclusion and exclusion criteria were recorded as indicator variables on the patient registration CRF. Patient were only registered if all inclusion criteria were documented as "yes" and all exclusion criteria were documented as "no". However, in few cases it was detected e.g. by monitoring on site, that an exclusion criterion was already met at registration. In these cases, the respective indicator variables were corrected by the local investigators.

In addition, some of the exclusion criteria could be cross checked with data recorded in the baseline CRF.

A violation of inclusion or exclusion criteria was considered present if:

- Any of the inclusion criteria was recorded as "no" or
- Any of the exclusion criteria was recorded as "yes" or
- **Left ventricular ejection fraction (LVEF)** derived as <= 30% (see 3.4.1) or
- At baseline ECG, **rhythm of the heart** was documented directly as "atrial fibrillation" or if it was as "other rhythm" and the explanatory plain text on the CRF indicated presence of atrial fibrillation or
- An antiarrhytmic drug was recorded as further concomitant medication (plain text variable) at baseline or
- **Date of a prior myocardial infarction** documented in the medical history section was <= 7 days prior to randomisation or
- Baseline **Troponin T** was documented as ≥ 50 pg/ml or
- Baseline **creatinine** was documented as > 2,0 mg/dl (> 176 µmol/l) or
- In baseline lung function assessment, **FeV1** was documented as <=50% or
- Number of drinks per week as recorded on the baseline CRF exceeded a mean alcohol consumption of > 60g/day for males and > 40g/day for females or
- **Date of a prior stroke** documented in the medical history section was <= 2 months prior to randomisation

4.3.2 Intervention not per protocol

A major protocol violation with respect to the trial intervention was considered present if:

- **Intervention done** is recorded as "no" or
- **Intervention as randomized** is recorded as "no" or

- **Applied pressure according to protocol** is derived as “too low” or
- **Total duration of intervention as per protocol is derived as “no” or**
- **Blinding successful** is recorded as “no”

4.3.3 Surgery performed meeting an exclusion criterion

Surgery-related exclusion criteria comprised planned off-pump surgery, concomitant carotid surgery, minimal-invasive surgery, selective antegrade cerebral perfusion, aorta descendens surgery. However, in some cases, performed surgery deviated from planned surgery. In these cases performed surgery was recorded on the CRF as plain text description.

A major protocol violation with respect to surgery was considered as present if one of the excluded surgical procedures was described as performed on the CRF.

4.4 Subgroups

No subgroup analyses were pre-specified in the study protocol.

Exploratory subgroup analyses will be clearly labeled as post hoc.

4.5 Handling of center effects

Random center effects will be included in analytic models if feasible.

5 Planned analysis

A flowchart according to the CONSORT statement will describe the disposition of all patients registered to the trial detailing screening failure before randomization, withdrawals, drop-outs and inclusion in the analyses sets defined above. Respective listing will be provided. In addition, patients with major protocol violations will be listed.

Standard methods of descriptive statistics will be used always indicating the number of valid and missing values. Summary statistic will be reasonably rounded to avoid pseudo-precision.

General on the planned confirmatory analyses:

Each treatment comparison will be reported as a point estimate of the intervention effect on a meaningful scale, its 95% confidence interval and a respective p-value.

For each treatment comparison in the primary and secondary endpoints both a simple and a model based analysis will be provided.

- The simple, easy to communicate analyses will use widely known standard methods like the chi2-test or t-test and the associated confidence intervals for the underlying measure of difference.
- Advanced analyses will use generalized linear mixed models to adjust the treatment comparison for relevant covariates and possible random center effects.

If a relevant discordance between simple and advanced analysis arises, the model based approach is given preference in general; but the conflict has to be explored and the statistical report and any publication will mention and discuss the discrepancy.

5.1 Demographic and other baseline parameter

Demographic and other baseline parameter will be described for the whole FAS and by randomization arm.

5.2 Concomitant Diseases and Medication

Frequencies of concomitant diseases and medication will be described for the whole FAS and by randomization arm.

5.3 Compliance with regard to the Study Intervention

Patients will be listed on whom no intervention or an intervention not corresponding to the randomization arm was performed.

5.4 Pattern of Components of the primary endpoint

We will describe the frequencies of the observed patterns of components of the primary endpoint in cases with at least one complication. A Venn diagram will be used for illustration.

5.5 Primary endpoint

The study protocol specifies: Analysis of the binary composite primary endpoint is based on a logistic regression with treatment as fixed effect, adjusting for EuroSCORE, Diabetes status (Y/N) and concomitant use of Statin (y/n) (fixed effects) and Center (random effect).

The treatment difference is quantified as odds-ratio with 95% confidence interval. The test decision is based on the Wald test of the coefficient of treatment in the logistic regression.

Further specification of the primary analysis:

The EuroSCORE is entered as full score (as opposed to using the cut EuroSCORE 4+ as in stratification of the randomization) since the EuroSCORE was originally developed as linear predictor using logistic regression.

Statin use appeared as Cholesterol/Lipid lowering drugs (y/n) on the baseline CRF. This Variable is used.

The general linear mixed model is fitted using R package “lme4” with option: Gauss-Hermite Quadrature as integration method. For testing we used R package “lmerTest” using the Kenward & Roger method to determine the appropriate degrees of freedom, which is also used in SAS1. This choice of methodological details and software is in line with current published recommendations [4–7].

In addition, a simple estimate and 95% confidence interval for the difference in complication rates will be provided.

5.6 Secondary endpoints

5.6.1 Analysis of binary components of the composite endpoint

The same logistic regression model as for the primary endpoint is used in the analysis of the treatment effect in the separate binary components of the composite endpoint.

A table and a Forest plot of the confidence intervals of the intervention effect (both for model based odds ratios and crude differences in complication rates) for the primary composite endpoint and its binary components will be provided.

5.6.2 Analysis of time to composite endpoint and Overall survival

Time to event data will be described using Kaplan-Meier product limit estimator. The intervention effect will be analyzed with a Cox regression including the same covariates as in the analysis of the primary endpoint and including Center as random effect.

Validity of the proportional hazard assumption will be checked by graphical methods.

Impact of further covariates can be explored in supplementary analyses.

5.6.3 Analysis of time to occurrence of non-fatal endpoint components

Death from other causes is treated as a competing risk. Standard methods of Gray[9,10] for competing risk analysis as implemented in R package “cmprsk” will be used.

Cumulative incidence rates at d30, d90 and d365 will be estimated.

The intervention effect will be analyzed with the proportional subdistribution hazards regression model described in Fine and Gray[10] including the same covariates as in the analysis of the primary endpoint.

Impact of further covariates can be explored in supplementary analyses.

5.6.4 Analysis of Troponin

The analysis of the intervention effect on Troponin is complicated by the fact that most centers used Troponin T, but four centers used Troponin I instead of Troponin T, and one center switched between Troponin T and Troponin I for different patients.

In addition, upper normal values for Troponin T were uniform among centers, but varied for Troponin I.

Two separate analyses for Troponin T and Troponin I will be provided.

5.6.4.1 Analysis of Troponin T

Troponin T values will be analyzed on a log10 scale. Taking the logarithm transforms a heavily skewed distribution to an approximately symmetrical one.

The intervention effect on the maximum documented post surgery Troponin T value for each patient will be assessed within a linear mixed regression model including the same covariates as in the analysis of the primary endpoint and including Center as random effect.

In addition, the time course of Troponin T will be analyzed. In the protocol, Troponin measurements were scheduled at 6h, 12h, 24h, and 48h post operation. As exact times of the blood sampling were recorded these are used in the analysis specified below.

Blinded preparatory analysis revealed that Troponin T typically decreases from an early peak. This decrease is approximately linear if plotted against log(time after end of operation).

Thus we will fit a linear mixed model with a bivariate random patient effect on the intercept and the slope of the regression against log(time). After adjusting for the covariates specified for the analysis of the primary endpoint an intervention effect of the intercept and/or the slope will be assessed using the Wald test on the respective coefficient.

Impact of further covariates in particular type of surgery will be explored in supplementary analyses.

5.6.4.2 Analysis of Troponin I

Troponin I values will be analyzed in units of the respective center’s upper normal value and analyzed on the log10 scale.

The intervention effect on the maximum documented post operation Troponin I value for each patient will be assessed within a linear mixed regression model including the same covariates as in the analysis of the primary endpoint and including Center as random effect.

5.6.5 Analysis of Creatinine

Serum creatinine values will be analyzed in units of the center’s upper normal value and analyzed on the log10 scale.

The maximum documented post surgery serum creatinine value for each patient will be assessed within a linear mixed regression model including the same covariates as in the analysis of the primary endpoint and including Center as random effect.

5.6.6 Analysis of durations

- Cumulative duration of invasive ventilator support
- Cumulative duration of non-invasive ventilator support
- Length of stay on the intensive care unit

- Total hospital stay

In these endpoints for simple analysis the t-test is used. For model based analysis a linear mixed model analogous to the analysis of the primary endpoint is used.

5.7 Further outcomes

Further endpoints (presence of a condition post surgery) are binary. Analysis of the treatment effect in further binary variables follows the analysis strategy for the primary endpoint.

5.8 Sub-group analyses

We perform the following explorative sub-group analyses: Diabetes yes/no, EuroScore 0-2 versus 3-5 vs 6+, use of cholesterol or lipid lowering drugs yes/no and type of surgery CABG alone versus other type of surgery.

The test for interaction is performed adding an interaction term of the respective covariate with treatment to the basic logistic regression model used for the analysis of the primary endpoint (compare 5.5).

5.9 Safety aspects

Adverse Event (AE) and Serious Adverse Event (SAE) reporting was limited by the protocol (6.1.3) to a time horizon of 96h post operation and exclusively to AEs that concerned the patient’s arm on which the study intervention (RIPC/ShamRIPC) was performed or AEs otherwise suspected to be related to the RIPC/ShamRIPC intervention.

Few AEs were expected and reported.

Adverse events and serious adverse events will be listed by study arm.

6 References

1. Meybohm P, Zacharowski K, Cremer J, et al. Remote ischaemic preconditioning for heart surgery. The study design for a multi-center randomized double-blinded controlled clinical trial--the RIPHeart-Study. Eur Heart J. 2012 Jun;33(12):1423-6.
2. Thygesen K, Alpert JS, Jaffe AS, et al. Third universal definition of myocardial infarction. Eur Heart J. 2012 Oct;33(20):2551-67.
3. Thygesen K, Alpert JS, White HD, et al. Universal definition of myocardial infarction. Eur Heart J. 2007 Oct;28(20):2525-38.
4. Schaalje GB, McBride JB, Fellingham GW. Adequacy of approximations to distributions of test statistics in complex mixed linear models. JABES 2002;7:512–24.
5. Bolker BM, Brooks ME, Clark CJ, et al. Generalized linear mixed models: a practical guide for ecology and evolution. Trends in ecology & evolution 2009;24:127–35. eng.
6. Casals M, Girabent-Farrés M, Carrasco JL. Methodological quality and reporting of generalized linear mixed models in clinical medicine (2000-2012): a systematic review. PloS one 2014;9:e112653. eng.

7. Li B, Lingsma HF, Steyerberg EW, Lesaffre E. Logistic random effects regression models: a comparison of statistical packages for binary and ordinal outcomes. BMC medical research methodology 2011;11:77. eng.
8. Thiele J. Potential of GLMM in modelling invasive spread. CAB Reviews 2012;7.
9. Gray RJ. A Class of K-Sample Tests for Comparing the Cumulative Incidence of a Competing Risk. The Annals of Statistics 1988;16:1141–54.
10. Fine JP, Gray RJ. A Proportional Hazards Model for the Subdistribution of a Competing Risk. Journal of the American Statistical Association 1999;94:496–509.
11. Gould AL. Interim analyses for monitoring clinical trials that do not materially affect the type I error rate. Stat Med 1992;11:55-66.

7 Appendix

7.1 Abbreviations

AE	Adverse event
CABG	Coronary artery bypass graft
CPB	Cardiopulmonary bypass
CRF	Case Report Form
EC	Ethics Committee
ECG	Electrocardiogram
ECLS	Extracorporeal Life Support System
FAS	Full analysis set
GCP	Good Clinical Practice
ICH	International Conference on Harmonisation
IABP	Intra-aortic balloon pump
LVEF	Left ventricular ejection fraction
OPCAB	Off-Pump-Coronary-Artery-Bypass
PPS	Per protocol set
RIPC	Remote ischemic pre-conditioning
SAE	Serious adverse event
SAP	Statistical Analysis Plan
SAS	Safety analysis set

2. Statistical analysis plan - summary of changes

- In section 3.4 (Further Variables) a few derived variables were added (Aortic aneurysm, Mitral regurgitation, Aortic insufficiency, Aortic valve stenosis, Total duration of intervention as per protocol) or their classification was modified (Applied pressure according to protocol, Type of surgery).
- In section 4.3 an operational definition of major protocol violations was added.
- In 5.6.4 and 5.6.5 analyses of maximum post-surgery troponin and creatinine values are based on linear mixed models adjusting for the same covariates including Center as random effect as in the analysis of the primary endpoint.
- A section 5.8 on explorative sub-group analyses was added.
