# Supplementary material for: Genome-wide association study of myocardial infarction, atrial fibrillation, acute stroke, acute kidney injury and delirium after cardiac surgery – a sub-analysis of the RIPHeart-Study
Source: BMC Cardiovasc Disord. 2019 Jan 24;19:26. doi: 10.1186/s12872-019-1002-x (PMC6345037; doi:10.1186/s12872-019-1002-x)
Supplement: Supplementary file 3 — Table S1. List of adjustment variables. (PDF 65 kb) [file 12872_2019_1002_MOESM3_ESM.pdf]

Name	Position	Label		Measurement Level	Format	Column Width	Alignment
PATNO	1	PatientID		Nominal	A8	8	Left
CNO	2	Centre	1	Nominal	F3	10	Right
AGE	3	Age	2	Scale	F3	8	Right
SEX	4	Gender	2	Nominal	F2	8	Right
RAUCH	5	Smoker	11	Nominal	F2	8	Right
ANZZIG	6	Smoker: packs per year	11	Scale	F3	8	Right
UHB_B	7	Hemoglobin Baseline [g/dL]	7	Scale	F8.1	10	Right
UKREA_B	8	Creatinine Baseline [mg/dL]	7	Scale	F8.2	10	Right
NIHSS_B	9	NIH Stroke Scale - baseline	18	Ordinal	F3	8	Right
KHKneu	10	Ischemic heart disease	18	Nominal	F2	10	Right
MIINS_ECHO	11	ECHO mitral regurgitation - baseline	18	Nominal	F2	8	Right
AOINS_ECHO	12	ECHO aortic insufficiency - baseline	18	Nominal	F2	8	Right
AOST_ECHO	13	ECHO aortic valve stenosis - baseline	18	Nominal	F2	8	Right
ANEURneu	14	Aortic aneurysm	18	Nominal	F2	10	Right
ZNMIneu	15	Previous myocardial infarction	18	Nominal	F2	10	Right
HERZINS	16	Heart failure	18	Nominal	F2	8	Right
COPD	17	COPD	21	Nominal	F2	8	Right
PAVK	18	Peripheral artery occlusive disease	19	Nominal	F2	8	Right
NIERE	19	Renal disease	30	Nominal	F2	8	Right
DM	20	Diabetes mellitus	22	Nominal	F2	8	Right
ZNSTROKE	21	Prior stroke	18	Nominal	F2	8	Right
AHT	22	Arterial hypertension	18	Nominal	F2	8	Right
CHOLESENK	23	Cholesterol lowering drug	13	Nominal	F2	8	Right
MBB	24	Beta blockers	13	Nominal	F2	8	Right
MACE	25	ACE inhibitor	13	Nominal	F2	8	Right
INSUL	26	Insuline	13	Nominal	F2	8	Right
ASS	27	ASS	13	Nominal	F2	8	Right
CLOP	28	Clopidogrel	13	Nominal	F2	8	Right
LVEF	29	LVEF	18	Nominal	F8.2	10	Right
NYHA	30	NYHA class	18	Nominal	F2	8	Right
EUROSCOR	31	EuroSCORE	18	Ordinal	F3	8	Right
PE	32	Primary endpoint including reference ECG information	18	Nominal	F4	10	Right
DEATH	33	Death according to CRF	1	Nominal	F4	10	Right
MI	34	Myocardial infarction incl. ECG reference information	18	Nominal	F4	10	Right
STROKE	35	Stroke according to CRF	18	Nominal	F4	10	Right
RENFAIL	36	Renal failure according to CRF	30	Nominal	F4	10	Right
AFF	37	New onset of atrial fibrillation (within 96h after surgery)	18	Nominal	F2	10	Right
DELIR	38	Delirium within 96h after surgery	18	Nominal	F2	10	Right
OPDURCH	39	Operation performed	18	Nominal	F5	10	Right
UTROPT_B	40	Troponin T Baseline [pg/ml]	13	Scale	F8.2	10	Right
UTROPT_06	41	Troponin T 6h [pg/ml]	13	Scale	F8.2	11	Right
UTROPT_12	42	Troponin T 12h [pg/ml]	13	Scale	F8.2	11	Right
UTROPT_24	43	Troponin T 24h [pg/ml]	13	Scale	F8.2	11	Right
UTROPT_48	44	Troponin T 48h [pg/ml]	13	Scale	F8.2	11	Right
UTROPI_B	45	Troponin I Baseline [ng/ml]	13	Scale	F8.2	10	Right
UTROPI_06	46	Troponin I 6h [ng/ml]	13	Scale	F8.2	11	Right
UTROPI_12	47	Troponin I 12h [ng/ml]	13	Scale	F8.2	11	Right
UTROPI_24	48	Troponin I 24h [ng/ml]	13	Scale	F8.2	11	Right
UTROPI_48	49	Troponin I 48h [ng/ml]	13	Scale	F8.2	11	Right
RIFLE	50	Modified RIFLE Classification (based on maximum creatinine)	30	Nominal	F2	10	Right
WundInf	51	Wound infection or re-operation due to wound healing problems	30	Unknown	F8.2	10	Right
SEPSIS_KP	52	Severe sepsis / septic shock	30	Nominal	F2	8	Right
REA_KP	53	Cardiopulmonary resuscitation	18	Nominal	F2	8	Right
ReThorak	54	Re-Thoracotomy	30	Nominal	F2	10	Right
AssistDevice_ICU	55	Use of any cardiac assist device - during ICU stay	18	Nominal	F2	18	Right
DELIRMED	56	Any delirium medication	13	Nominal	F2	10	Right

Value		Label	
PE	-88	PE ass. done, but inf. missing	30
PE	111	stroke + MI + death	19
PE	1010	renal failure + MI	18
PE	1011	renal failure + MI + death	26
PE	1110	renal failure + stroke + MI	27
PE	1111	RF + stroke + MI + death	24
MI	100	yes,<=72h Troponin+new LBB	26
MI	1000	yes,<=72h Troponin+new Q-wave	29
MI	1001	yes,<=72hTropo.+newQ-wave+Angi	30
MI	1100	yes,<=72hTropo.+nQ-wave+newLBB	30
STROKE	11	yes, inhospital+aft. Discharge	30
RENFAIL	11	yes, uri outp+ mx.creat	23
RENFAIL	111	yes, RRT+uri outp+ mx.creat	27
RENFAIL	1001	yes,autopsy + max creatinine	28
RENFAIL	1010	yes, autopsy + urine output	27
RENFAIL	1011	yes,autopsy+uri outp+max creat	30
RENFAIL	1100	yes,autopsy + RRT	17
RENFAIL	1101	yes, autopsy+RRT+mx creatinine	30
RENFAIL	1110	yes, autopsy+RRT+urine outp.	28
RENFAIL	1111	yes,autopsy+RRT+uri out+m.crea	30
OPDURCH	2	Aortic valve repl. (alone)	26
OPDURCH	3	Mitral val.repl./recon.(alone)	30
OPDURCH	4	Aorta ascendens repl. (alone)	29
CNO	1	Kiel	4
CNO	2	Aachen	6
CNO	3	Bonn	4
CNO	4	Duesseldorf	11
CNO	5	Frankfurt	9
CNO	6	Göttingen	9
CNO	7	Magdeburg	9
CNO	8	Rostock	7
CNO	9	Luebeck	7
CNO	10	Wuerzburg	9
CNO	11	Berlin	6
CNO	12	Giessen	7
CNO	13	Mainz	5
CNO	14	Erlangen	8
CNO	15	Jena	4
SEX	1	male	4
SEX	2	female	6
RAUCH	1	never	5
RAUCH	2	>= 6 months clean	17
RAUCH	3	current	7
KHKneu	0	no	2
KHKneu	1	yes	3
KHKneu	99	unknown	7
MIINS_ECHO	1	none	4
MIINS_ECHO	2	mild	4
MIINS_ECHO	3	moderate	8
MIINS_ECHO	4	severe	6
AOINS_ECHO	1	none	4
AOINS_ECHO	2	mild	4
AOINS_ECHO	3	moderate	8
AOINS_ECHO	4	severe	6
AOST_ECHO	1	none	4
AOST_ECHO	2	mild	4
AOST_ECHO	3	moderate	8
AOST_ECHO	4	severe	6
ANEURneu	0	no	2
ANEURneu	1	yes	3
ZNMIneu	0	no	2
ZNMIneu	1	yes	3
ZNMIneu	99	unknown	7
HERZINS	0	no	2
HERZINS	1	yes	3
HERZINS	99	unknown	7
COPD	0	no	2
COPD	1	yes	3
COPD	99	unknown	7
PAVK	0	no	2
PAVK	1	yes	3
PAVK	99	unknown	7
NIERE	0	no	2
NIERE	1	yes	3
NIERE	99	unknown	7
DM	0	no	2
DM	1	yes	3
DM	99	unknown	7
ZNSTROKE	0	no	2
ZNSTROKE	1	yes	3
ZNSTROKE	99	unknown	7
AHT	0	no	2
AHT	1	yes	3
AHT	99	unknown	7
CHOLESENK	0	no	2
CHOLESENK	1	yes	3
MBB	0	no	2
MBB	1	yes	3
MACE	0	no	2
MACE	1	yes	3
INSUL	0	no	2
INSUL	1	yes	3
ASS	0	no	2
ASS	1	yes	3
CLOP	0	no	2
CLOP	1	yes	3
LVEF	1	>=55%	5
LVEF	2	30-55%	6
LVEF	3	<30%	4
NYHA	1	NYHA I	6
NYHA	2	NYHA II	7
NYHA	3	NYHA III	8
NYHA	4	NYHA IV	7
PE	-99	unknown	7
PE	0	no	2
PE	1	death	5
PE	10	myocardial infarction	21
PE	11	myocardial infarction + death	29
PE	100	stroke	6
PE	101	stroke + death	14
PE	110	stroke + myocardial infraction	30
PE	1000	renal failure	13
PE	1001	renal failure + death	21
PE	1100	renal failure + stroke	22
PE	1101	renal failure + stroke + death	30
DEATH	-99	unknown	7
DEATH	-88	inconsistencies???	18
DEATH	0	no	2
DEATH	1	yes, myocardial infarction	26
DEATH	2	yes, arrhytmia	14
DEATH	3	yes, asystole	13
DEATH	4	yes, pump failure	17
DEATH	5	yes, other cardiac	18
DEATH	6	yes, cardiac?	13
DEATH	7	yes, pneumonia	14
DEATH	8	yes, sepsis	11

PatientID	$ODBC.Name	PATNO
	$ODBC.Size	8
	$ODBC.Type	12
Age	$ODBC.Name	AGE
	$ODBC.Size	3
	$ODBC.Type	3
Gender	$ODBC.Name	SEX
	$ODBC.Size	1
	$ODBC.Type	3
Smoker	$ODBC.Name	RAUCH
	$ODBC.Size	1
	$ODBC.Type	3
Smoker: packs per year	$ODBC.Name	ANZZIG
	$ODBC.Size	4
	$ODBC.Type	3
NIH Stroke Scale - baseline	$ODBC.Name	NIHSS_B
	$ODBC.Size	2
	$ODBC.Type	3
ECHO mitral regurgitation - baseline	$ODBC.Name	MIINS_ECHO
	$ODBC.Size	1
	$ODBC.Type	3
ECHO aortic insufficiency - baseline	$ODBC.Name	AOINS_ECHO
	$ODBC.Size	1
	$ODBC.Type	3
ECHO aortic valve stenosis - baseline	$ODBC.Name	AOST_ECHO
	$ODBC.Size	1
	$ODBC.Type	3
Heart failure	$ODBC.Name	HERZINS
	$ODBC.Size	2
	$ODBC.Type	3
COPD	$ODBC.Name	COPD
	$ODBC.Size	2
	$ODBC.Type	3
Peripheral artery occlusive disease	$ODBC.Name	PAVK
	$ODBC.Size	2
	$ODBC.Type	3
Renal disease	$ODBC.Name	NIERE
	$ODBC.Size	2
	$ODBC.Type	3
Diabetes mellitus	$ODBC.Name	DM
	$ODBC.Size	2
	$ODBC.Type	3
Prior stroke	$ODBC.Name	ZNSTROKE
	$ODBC.Size	2
	$ODBC.Type	3
Arterial hypertension	$ODBC.Name	AHT
	$ODBC.Size	2
	$ODBC.Type	3
Cholesterol lowering drug	$ODBC.Name	CHOLESENK
	$ODBC.Size	1
	$ODBC.Type	3
Beta blockers	$ODBC.Name	MBB
	$ODBC.Size	1
	$ODBC.Type	3
ACE inhibitor	$ODBC.Name	MACE
	$ODBC.Size	1
	$ODBC.Type	3
Insuline	$ODBC.Name	INSUL
	$ODBC.Size	1
	$ODBC.Type	3
ASS	$ODBC.Name	ASS
	$ODBC.Size	1
	$ODBC.Type	3
Clopidogrel	$ODBC.Name	CLOP
	$ODBC.Size	1
	$ODBC.Type	3
NYHA class	$ODBC.Name	NYHA
	$ODBC.Size	1
	$ODBC.Type	3
EuroSCORE	$ODBC.Name	EUROSCOR
	$ODBC.Size	2
	$ODBC.Type	3
Severe sepsis / septic shock	$ODBC.Name	SEPSIS_KP
	$ODBC.Size	1
	$ODBC.Type	3
Cardiopulmonary resuscitation	$ODBC.Name	REA_KP
	$ODBC.Size	1
	$ODBC.Type	3

DEATH	9	yes, tumour	11
DEATH	10	yes, cerebrovascular accident	29
DEATH	11	yes, pulmonary embolism	23
DEATH	12	yes, other non-cardiac	22
DEATH	13	yes, other non-cardiac ?	24
DEATH	14	yes, unknown cause	18
MI	-99	unknown	7
MI	-88	inconsistencies???	18
MI	0	no	2
MI	1	yes, <=72h by Troponin + Angio	30
MI	10	yes, <=72h by Troponin + Echo	29
MI	10000	yes, > 72h postoperative	24
MI	100000	yes, according to autopsy	25
STROKE	-99	unknown	7
STROKE	-88	inconsistencies???	18
STROKE	0	no	2
STROKE	1	yes, in hospital	16
STROKE	10	yes, after discharge	20
STROKE	100	yes, according to autopsy	25
RENFAIL	-99	unknown	7
RENFAIL	-88	inconsistencies???	18
RENFAIL	0	no	2
RENFAIL	1	yes, max creatinine	19
RENFAIL	10	yes, urine output	17
RENFAIL	100	yes, RRT	8
RENFAIL	101	yes, RRT + max creatinine	25
RENFAIL	110	yes, RRT + urine output	23
RENFAIL	1000	yes, according to autopsy	25
AFF	0	no new AFF	10
AFF	1	new AFF	7
AFF	2	preexisting AFF	15
DELIR	-99	not evaluable	13
DELIR	0	no	2
DELIR	1	yes	3
OPDURCH	1	CABG (alone)	12
OPDURCH	5	Combined procedures	19
OPDURCH	6	Other type of surgery (alone)	29
RIFLE	1	No impairement	14
RIFLE	2	Risk	4
RIFLE	3	Injury	6
RIFLE	4	Failure	7
RIFLE	99	unknown	7
WundInf	0	no	2
WundInf	1	yes	3
SEPSIS_KP	0	no	2
SEPSIS_KP	1	yes	3
REA_KP	0	no	2
REA_KP	1	yes	3
ReThorak	0	no	2
ReThorak	1	yes	3
AssistDevice_ICU	0	no	2
AssistDevice_ICU	1	yes	3
DELIRMED	0	no	2
DELIRMED	1	yes	3
