# Supplementary figures and images for: Genome-wide association study of myocardial infarction, atrial fibrillation, acute stroke, acute kidney injury and delirium after cardiac surgery – a sub-analysis of the RIPHeart-Study
Source: BMC Cardiovasc Disord. 2019 Jan 24;19:26. doi: 10.1186/s12872-019-1002-x (PMC6345037; doi:10.1186/s12872-019-1002-x)

AFF

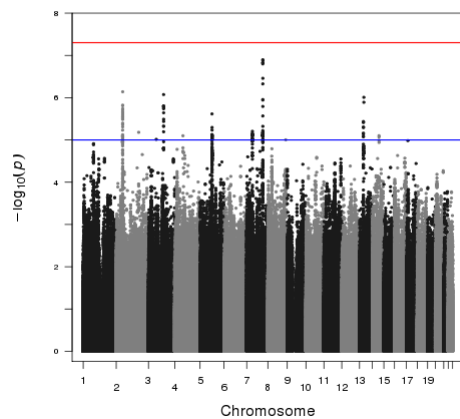

DELIR

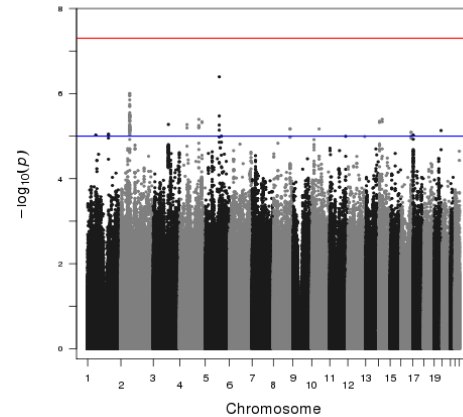

MI

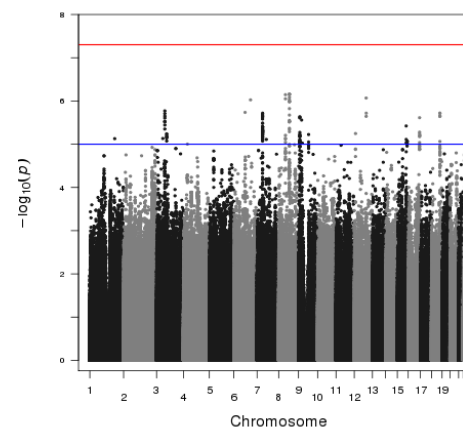

RENFAIL

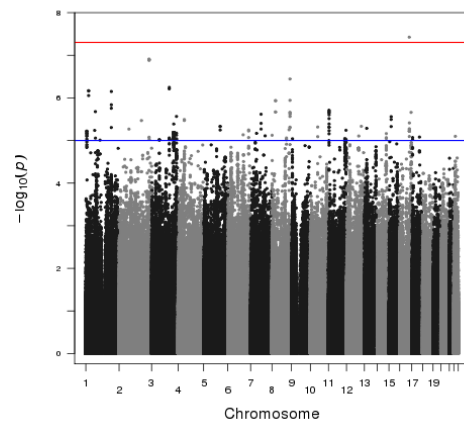

STROKE

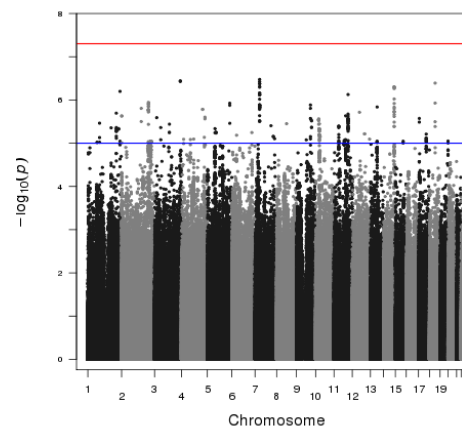

S1 Fig a

AFF

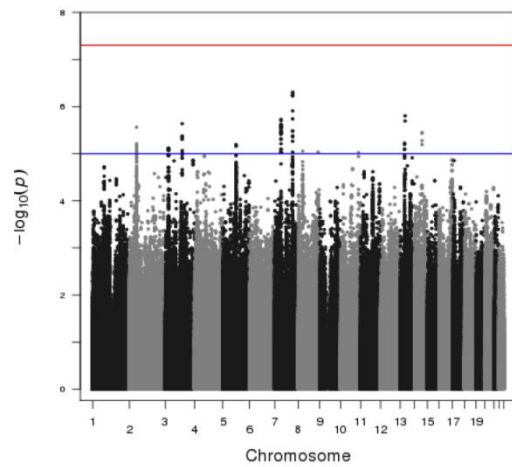

DELIR

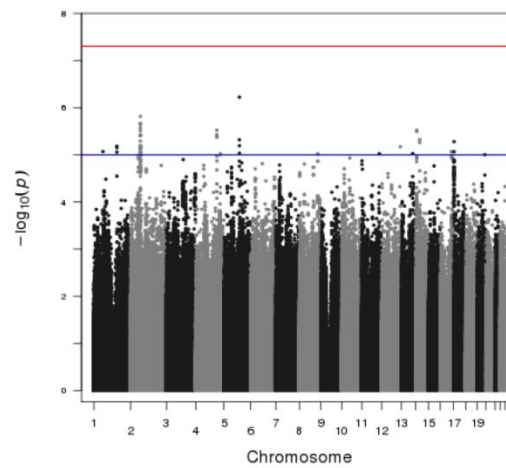

MI

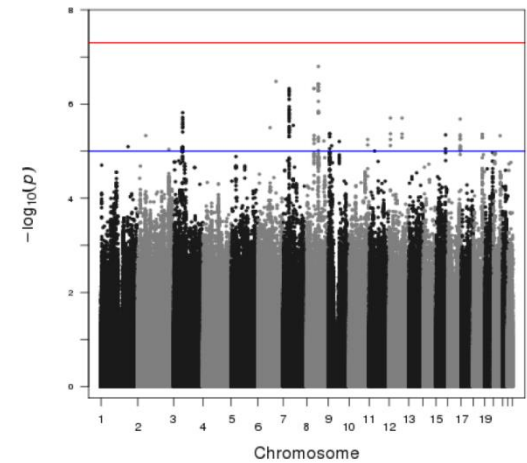

RENFAIL

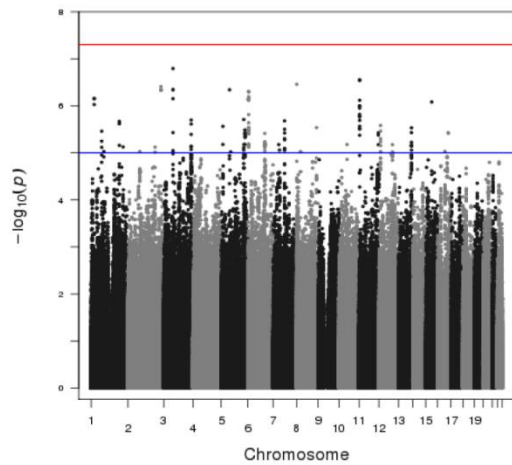

STROKE

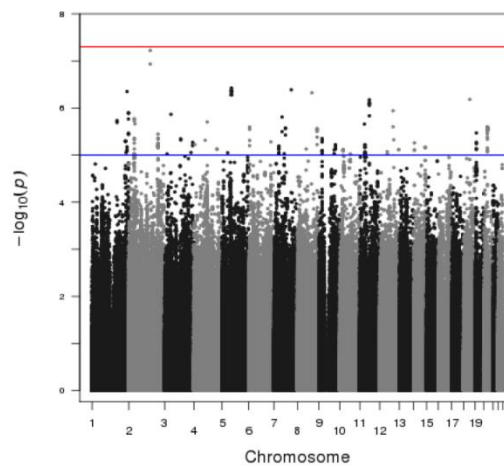

ALL

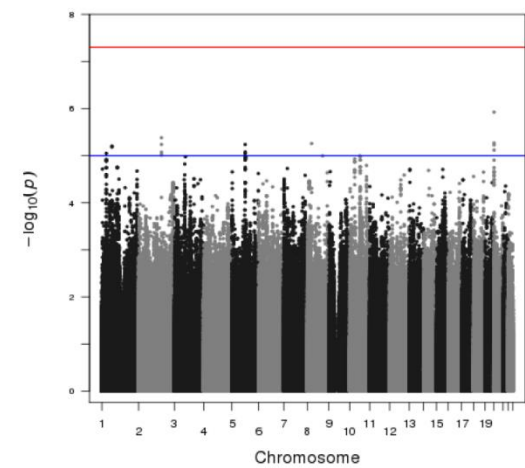

S1 Fig b

Supplement: Supplementary file 4 — Figure S1. Manhattan Plot of genome-wide association with AF, delirium, MI, renal failure and stroke. The x-axis represents the chromosomes in physical order, the y axis showing –log10(p) for all single nucleotide polymorphisms (SNPs). a) Adjustment for all other outcomes: one SNP reached genome-wide significance (p < 5 × 10− 8, red line) and 139 SNPs reached the predefined threshold of p < 1 × 10− 5 (blue line). b) No adjustment for all other outcomes and composite: 132 SNPs reached the predefined threshold of p < 1 × 10− 5. (PDF 498 kb) [file 12872_2019_1002_MOESM4_ESM.pdf]

# AFF

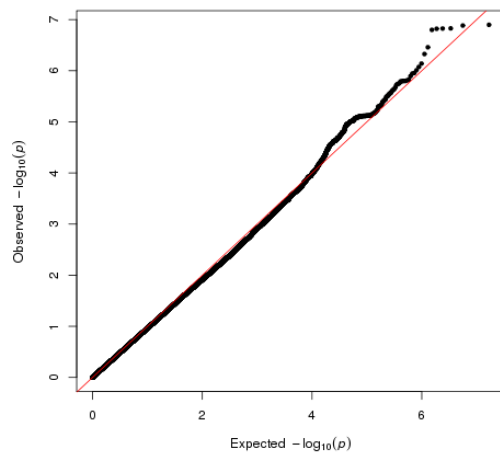

# DELIR

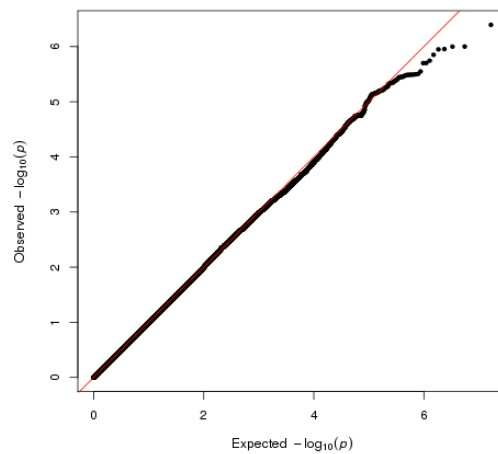

# MI

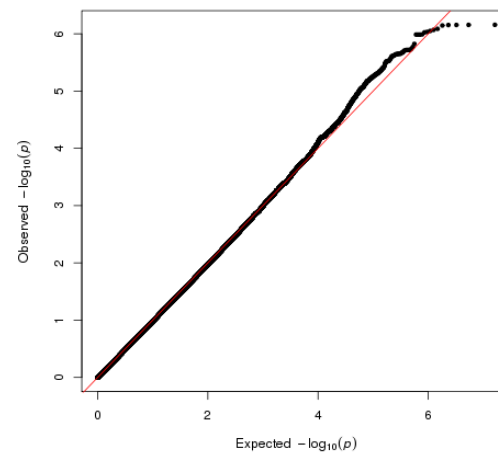

# RENFAIL

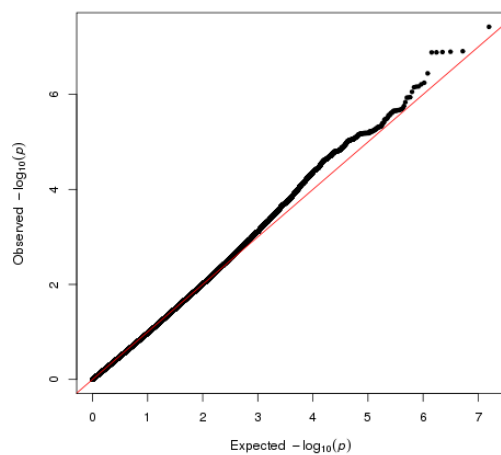

# STROKE

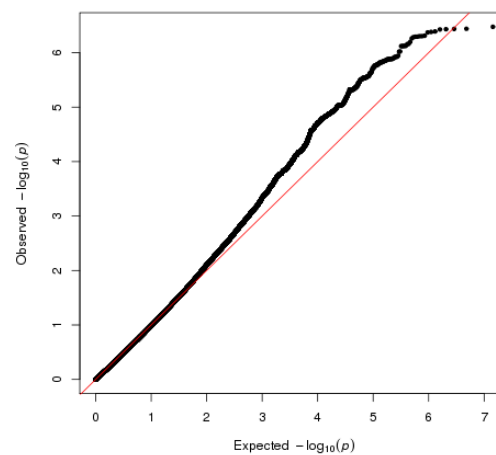

S2 Fig a

AFF

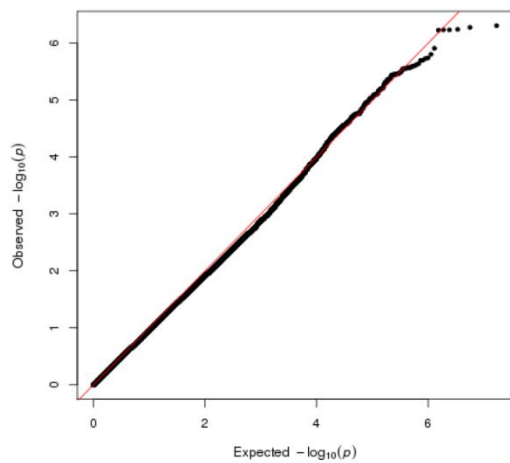

DELIR

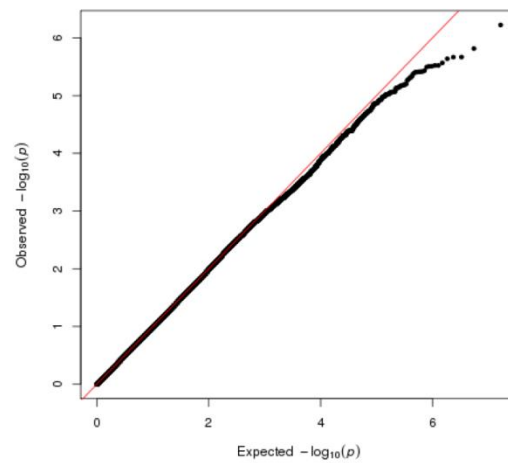

MI

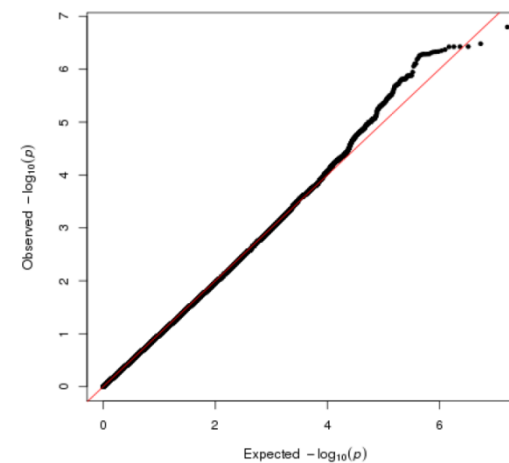

RENFAIL

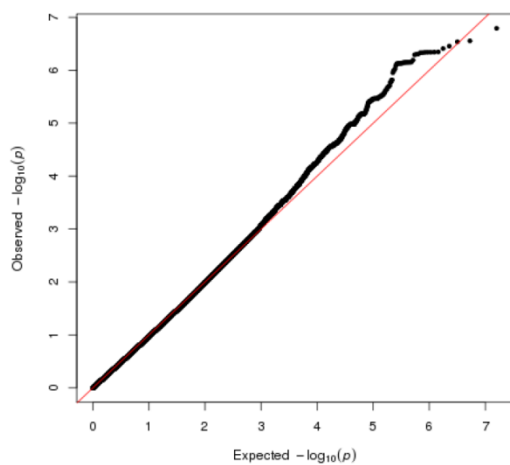

STROKE

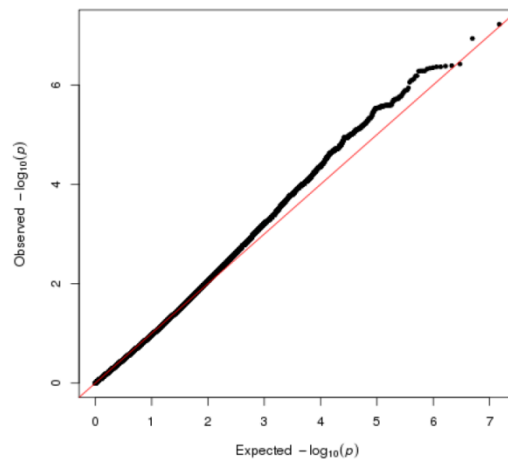

ALL

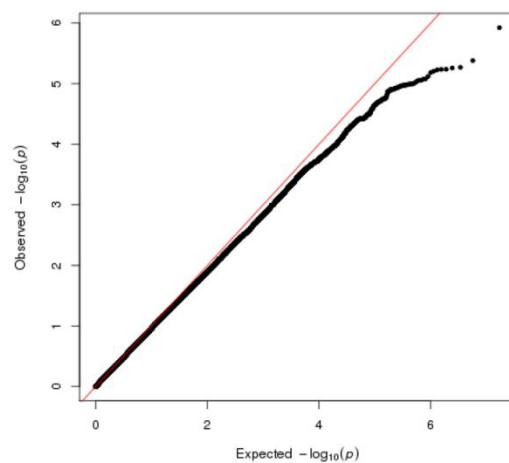

Supplement: Supplementary file 5 — Figure S2. Quantile-quantile plot showing the expected distribution of association test statistics across the SNPs compared to the observed values for AF, delirium, MI, renal failure and stroke. a) Adjustment for all other outcomes. b) No adjustment for all other outcomes and composite. (PDF 305 kb) [file 12872_2019_1002_MOESM5_ESM.pdf]
