# Supplementary material for: Genome-wide association study of myocardial infarction, atrial fibrillation, acute stroke, acute kidney injury and delirium after cardiac surgery – a sub-analysis of the RIPHeart-Study
Source: BMC Cardiovasc Disord. 2019 Jan 24;19:26. doi: 10.1186/s12872-019-1002-x (PMC6345037; doi:10.1186/s12872-019-1002-x)
Supplement: Supplementary file 6 — Table S2. Complete table of SNPs reaching the predefined threshold of p < 1 × 10− 5 with and without adjustment for all other complications. (PDF 1177 kb) [file 12872_2019_1002_MOESM6_ESM.pdf]

Table S2

Adjusted for all other outcomes

CHR	SNP	BP	A1	A2	FRQ	P	OR_L95	OR_U95	gene	conseq	genotyped	impute2.info
7	rs17512172	121198154	T	A	0.67	1.27e-07	0.43	0.68	-	intergenic_variant	0	0.997
2	rs7557600	48772202	T	C	0.83	7.25e-07	0.37	0.65	STON1, STON1-GTF2A1L, -	intron_variant, regulatory_region_variant	0	0.991
3	rs115155878	114055988	A	C	0.97	8.45e-07	0.11	0.39	ZBTB20	downstream_gene_variant	0	0.991
13	rs9563027	51708406	T	C	0.69	9.82e-07	0.45	0.71	LINC00371	intron_variant, non_coding_transcript_variant	0	0.978
5	rs4574581	89925895	G	A	0.34	2.43e-06	0.42	0.70	GPR98	intron_variant	0	0.984
13	rs1974039	48475331	G	A	0.61	3.67e-06	0.48	0.74	LINC00444	downstream_gene_variant	0	0.998
5	rs77190517	90216068	C	T	0.96	5.06e-06	0.20	0.52	GPR98	intron_variant	0	0.992
2	rs58835962	48328813	G	C	0.88	5.86e-06	0.37	0.67	AC079807.4	intron_variant, non_coding_transcript_variant	0	0.994
7	rs4442037	42559895	C	G	0.27	6.25e-06	1.35	2.12	-	intergenic_variant	0	0.993
14	rs150942	70542102	G	A	0.34	7.93e-06	0.43	0.72	SLC8A3	intron_variant, intron_variant, NMD_transcript_variant	0	0.961
4	rs1551049	62105676	A	G	0.67	7.95e-06	1.38	2.28	LPHN3	intron_variant, intron_variant, non_coding_transcript_variant	0	0.969
3	rs115978432	58525210	C	A	0.99	9.69e-06	0.06	0.34	ACOX2	upstream_gene_variant	0	0.993
8	rs58527822	134785991	C	T	0.92	9.94e-06	0.29	0.62	CTD-2588J1.1, CTD-2309H9.3	intron_variant, non_coding_transcript_variant, downstream_gene_variant	0	0.971
5	rs10051505	105009840	T	C	0.92	4.03e-07	0.14	0.42	-	intergenic_variant	0	0.975
2	rs13008718	65961565	G	T	0.56	9.99e-07	0.33	0.62	AC074391.1, RP11-340F16.1	intron_variant, non_coding_transcript_variant, downstream_gene_variant	0	0.986
14	rs188623516	46926170	T	A	0.99	4.02e-06	0.00	0.10	LINC00871	intron_variant, non_coding_transcript_variant	0	0.998
4	chr4:142710442	142710442	C	A	0.70	4.04e-06	1.64	3.36	-	intergenic_variant	2	1
14	rs73591732	27448446	T	C	0.90	4.48e-06	0.23	0.56	RP11-384J4.2	intron_variant, non_coding_transcript_variant	0	0.998
4	rs10015432	166855253	T	C	0.90	4.59e-06	0.21	0.53	TLL1	intron_variant, intron_variant, NMD_transcript_variant	0	0.957
3	rs74888047	112614048	C	A	0.99	5.32e-06	0.02	0.20	-	intergenic_variant	0	0.999
4	rs4864785	54394410	C	T	0.77	5.37e-06	1.81	4.39	LNX1-AS1, LNX1, FIP1L1	intron_variant, non_coding_transcript_variant, intron_variant, downstream_gene_variant	0	0.947
8	rs13281094	124741789	T	C	0.64	6.71e-06	0.35	0.66	ANXA13, -	upstream_gene_variant, intron_variant, regulatory_region_variant	0	0.98
10	rs78696618	55268813	G	A	1.00	6.78e-06	0.00	0.08	-	intergenic_variant	0	0.998
19	rs192196148	51983352	G	C	1.00	7.39e-06	0.00	0.09	CEACAM18, -	intron_variant, regulatory_region_variant	0	0.999
16	rs9922610	76512728	G	A	0.99	8.11e-06	0.01	0.18	CNTNAP4	intron_variant, non_coding_transcript_variant, intron_variant	0	1

1	rs74634803	156207690	G	C	0.99	9.00e-06	0.03	0.25	PMF1, PMF1-BGLAP, BGLAP	intron_variant, downstream_gene_variant, intron_variant, NMD_transcript_variant, upstream_gene_variant	0	0.993
1	rs180991369	61102621	G	A	0.97	9.41e-06	0.08	0.37	RP11-776H12.1	intron_variant, non_coding_transcript_variant	0	0.987
17	rs4362428	2090341	C	A	0.60	9.46e-06	0.37	0.68	SMG6	intron_variant	0	0.997
8	rs13275931	79168795	C	T	0.92	6.96e-07	0.21	0.51	-	intergenic_variant	0	0.995
8	rs7819484	49778212	T	C	0.96	7.16e-07	0.12	0.39	-	intergenic_variant	0	0.986
12	rs6538067	87650216	G	T	0.12	8.55e-07	1.84	4.08	-	intergenic_variant	0	0.988
6	rs144756202	122373287	A	G	0.98	9.44e-07	0.08	0.34	-	intergenic_variant	0	0.989
3	rs727476	60316417	G	T	0.53	1.70e-06	0.33	0.63	FHIT	intron_variant	0	0.994
6	rs193051711	82737197	C	T	0.99	1.84e-06	0.02	0.18	-	intergenic_variant	0	0.999
7	rs9690969	40541259	T	C	0.82	1.91e-06	0.31	0.61	SUGCT	intron_variant, downstream_gene_variant	0	0.997
18	rs79164706	61879513	G	A	0.97	1.91e-06	0.12	0.42	RP11-909B2.1, RP11-146N18.1	downstream_gene_variant, intron_variant, non_coding_transcript_variant	0	0.997
16	rs17750019	83277510	A	G	0.89	2.43e-06	0.23	0.54	CDH13	intron_variant, NMD_transcript_variant, intron_variant, intron_variant, non_coding_transcript_variant	0	0.977
9	rs4298578	22834741	T	A	0.96	2.76e-06	0.12	0.42	-	intergenic_variant	0	0.995
15	rs191166845	87015139	T	C	0.99	3.78e-06	0.04	0.26	AGBL1	intron_variant	0	0.999
3	rs59137280	74071304	A	G	0.91	5.76e-06	0.25	0.58	-	intergenic_variant	0	0.987
9	rs4744652	74639028	T	C	0.15	5.98e-06	1.60	3.27	-	intergenic_variant	0	0.995
16	rs117092646	85199964	A	G	0.98	6.25e-06	0.10	0.40	CTC-786C10.1	upstream_gene_variant	0	0.996
1	rs115246628	183787321	C	T	1.00	7.44e-06	0.01	0.14	RGL1	intron_variant	0	0.998
7	rs74476624	67956247	G	C	0.97	7.76e-06	0.12	0.44	-	intergenic_variant	0	0.996
15	rs74317074	94177001	A	G	0.99	8.47e-06	0.04	0.30	RP11-739G5.1	intron_variant, non_coding_transcript_variant	0	0.999
9	rs147007521	28306823	G	C	0.96	9.43e-06	0.15	0.48	LINGO2	intron_variant	0	0.989
4	rs112955975	29332523	T	G	0.96	9.95e-06	0.18	0.51	-	intergenic_variant	0	0.996
16	rs78064607	71723181	C	T	0.99	3.77e-08	0.01	0.09	PHLPP2	intron_variant, intron_variant, non_coding_transcript_variant, upstream_gene_variant	0	0.997
2	rs184936958	224157963	C	T	1.00	1.23e-07	0.00	0.05	-	intergenic_variant	0	0.999
8	rs189437718	134655419	A	G	0.99	3.60e-07	0.01	0.15	SNORA40	downstream_gene_variant	0	0.996
1	rs72654815	21354625	G	A	0.97	6.79e-07	0.05	0.27	EIF4G3, -	intron_variant, regulatory_region_variant	0	0.987
1	rs113318533	191203487	C	A	0.96	7.08e-07	0.08	0.33	-	intergenic_variant	0	0.992
8	rs143062081	26797906	G	A	0.98	1.16e-06	0.05	0.27	-	intergenic_variant	0	0.988
11	rs139967808	4503396	G	A	0.99	1.95e-06	0.01	0.17	-	regulatory_region_variant, intergenic_variant	0	0.997
1	rs76487613	72134192	T	A	0.99	2.10e-06	0.03	0.22	NEGR1, -	intron_variant, regulatory_region_variant	0	0.996
16	rs72801516	84969387	A	G	0.66	2.19e-06	0.21	0.53	RP11-254F19.3	intron_variant, non_coding_transcript_variant	0	0.941

7	rs117208670	77885127	T	C	0.98	2.42e-06	0.04	0.26	MAGI2	3_prime_UTR_variant, intron_variant, non_coding_transcript_variant, intron_variant	0	0.994
15	rs188722224	38535852	C	T	0.99	2.76e-06	0.01	0.16	-	intergenic_variant	0	0.998
4	rs10212651	46543306	A	G	1.00	3.22e-06	0.00	0.11	-	intergenic_variant	0	0.998
2	rs140459083	166887177	C	G	0.99	3.40e-06	0.03	0.22	AC010127.3, SCN1A	intron_variant, non_coding_transcript_variant, intron_variant	0	0.997
16	rs140394213	72499282	G	A	0.99	3.87e-06	0.01	0.15	AC004158.2	intron_variant, non_coding_transcript_variant	0	1
3	rs140332909	164997469	C	T	1.00	4.11e-06	0.00	0.05	RP11-85M11.2	intron_variant, non_coding_transcript_variant	0	0.999
12	chr12:110925718	110925718	A	G	0.99	4.64e-06	0.03	0.23	FAM216A, VPS29	non_coding_transcript_exon_variant, non_coding_transcript_variant, downstream_gene_variant, missense_variant	2	1
5	rs192526962	123429995	A	G	1.00	4.67e-06	0.00	0.09	-	intergenic_variant	0	0.999
10	rs191017384	54876442	C	T	1.00	4.83e-06	0.01	0.12	-	intergenic_variant	0	1
13	rs111697328	36720672	T	C	0.93	5.18e-06	0.13	0.44	-	intergenic_variant	0	0.964
2	rs147332456	73467224	C	G	0.99	5.41e-06	0.01	0.15	CCT7, -	upstream_gene_variant, intron_variant, intron_variant, NMD_transcript_variant, intron_variant, non_coding_transcript_variant, non_coding_transcript_exon_variant, non_coding_transcript_variant, regulatory_region_variant	0	0.998
6	rs9478662	155912941	A	G	0.99	5.67e-06	0.03	0.25	-	intergenic_variant	0	0.999
11	rs11222654	131333844	G	A	0.99	5.75e-06	0.01	0.18	NTM	intron_variant, non_coding_transcript_variant, intron_variant	0	0.999
1	rs114460275	7552998	C	T	0.98	6.04e-06	0.05	0.30	CAMTA1	intron_variant	0	0.998
7	rs193083142	43997164	G	A	1.00	6.79e-06	0.00	0.08	RP5-1165K10.2, UBE2D4, AC004985.12, POLR2J4	intron_variant, non_coding_transcript_variant, downstream_gene_variant	0	1
14	rs57562323	87276346	T	C	0.91	6.89e-06	0.16	0.48	-	intergenic_variant	0	0.991
6	rs58258587	114638966	C	T	0.94	7.35e-06	0.12	0.44	HS3ST5, RP3-399L15.3	intron_variant, intron_variant, non_coding_transcript_variant	0	0.978
8	rs143497687	1256659	T	C	0.99	7.53e-06	0.01	0.19	-	intergenic_variant	0	0.998
7	rs73193454	108223617	T	A	0.98	7.77e-06	0.05	0.31	-	intergenic_variant	0	0.988
12	rs10745552	91469729	T	C	0.03	7.85e-06	2.69	12.48	-	intergenic_variant	0	0.998
22	rs112403136	29031126	G	A	0.97	7.98e-06	0.08	0.38	TTC28	intron_variant, intron_variant, non_coding_transcript_variant	0	0.985

17	rs144471398	57547816	G	A	0.99	8.30e-06	0.01	0.17	RP11-567L7.6	intron_variant, non_coding_transcript_variant	0	0.998
17	rs60508831	6612682	A	G	0.77	8.54e-06	0.21	0.54	SLC13A5	intron_variant, intron_variant, NMD_transcript_variant, upstream_gene_variant	0	0.916
8	rs180696447	108436114	G	T	0.99	8.75e-06	0.03	0.27	ANGPT1	intron_variant	0	0.996
9	rs181096409	7472406	A	G	0.99	9.12e-06	0.01	0.15	RPL4P5	downstream_gene_variant	0	0.997
11	rs77876049	122936811	G	A	0.95	9.14e-06	0.14	0.46	HSPA8	upstream_gene_variant	0	0.999
2	rs150163435	225106755	C	T	0.99	9.32e-06	0.01	0.15	AC104802.1	upstream_gene_variant	0	0.997
3	rs2361350	57764197	A	G	0.09	9.55e-06	1.99	5.88	SLMAP, -	intron_variant, non_coding_transcript_variant, intron_variant, regulatory_region_variant	0	0.999
1	rs190886391	106605878	T	A	0.99	9.80e-06	0.01	0.18	-	intergenic_variant	0	0.997
7	rs4098926	33511328	A	C	0.99	3.35e-07	0.00	0.06	BBS9, -	intron_variant, intron_variant, NMD_transcript_variant, regulatory_region_variant	0	0.996
3	rs181832941	189567428	T	C	1.00	3.65e-07	0.00	0.06	TP63	intron_variant, intron_variant, non_coding_transcript_variant	0	0.998
18	rs140914711	41414229	T	C	0.98	4.07e-07	0.01	0.13	RNU6-443P	upstream_gene_variant	0	0.993
14	rs188208602	98385236	C	T	0.99	4.98e-07	0.00	0.06	-	intergenic_variant	0	0.999
1	rs192540202	237511541	C	G	0.99	6.33e-07	0.00	0.08	RYR2	intron_variant	0	0.996
11	rs72982705	103566801	C	A	0.89	7.49e-07	0.04	0.26	RP11-563P16.1	intron_variant, non_coding_transcript_variant	0	0.997
2	rs4241192	197794166	G	T	0.10	1.13e-06	3.52	18.89	PGAP1	upstream_gene_variant	0	0.994
5	rs147719528	165130442	T	A	0.99	1.19e-06	0.00	0.07	CTC-535M15.2	intron_variant, non_coding_transcript_variant	0	0.997
13	rs186592710	68901484	A	T	0.99	1.46e-06	0.01	0.13	RPL37P21	downstream_gene_variant	0	0.994
2	rs150728297	144599994	A	G	0.99	1.56e-06	0.01	0.11	-	regulatory_region_variant, intergenic_variant	0	0.999
4	rs148632338	156047536	C	T	0.99	1.65e-06	0.00	0.10	-	intergenic_variant	0	0.997
12	rs117277472	52793574	C	T	0.99	1.93e-06	0.00	0.07	KRT82, RP3-416H24.4	intron_variant, intron_variant, non_coding_transcript_variant	0	0.998
1	rs187653925	209939702	A	G	0.99	2.02e-06	0.00	0.08	TRAF3IP3, -	intron_variant, upstream_gene_variant, downstream_gene_variant, intron_variant, NMD_transcript_variant, regulatory_region_variant	0	0.996
2	rs75236429	755099	A	T	0.95	2.33e-06	0.03	0.25	-	intergenic_variant	0	0.988
11	rs111451843	85833109	C	G	0.98	2.33e-06	0.01	0.16	-	intergenic_variant	0	0.995
4	rs181248603	173255832	T	A	0.99	2.50e-06	0.00	0.09	GALNTL6	intron_variant, non_coding_transcript_variant, intron_variant	0	0.995
3	rs75169866	15819505	T	C	0.97	2.57e-06	0.02	0.19	ANKRD28	downstream_gene_variant, intron_variant, NMD_transcript_variant, intron_variant, intron_variant, non_coding_transcript_variant	0	0.989

10	rs184966137	32249822	C	G	1.00	6.98e-06	0.00	0.04	-	intergenic_variant	0	1
4	rs185049893	168087142	A	T	0.99	7.31e-06	0.01	0.13	SPOCK3	intron_variant, NMD_transcript_variant, intron_variant	0	0.999
17	chr17:56290388	56290388	G	A	0.99	7.66e-06	0.00	0.10	MKS1	3_prime_UTR_variant, NMD_transcript_variant, synonymous_variant, downstream_gene_variant, upstream_gene_variant	0	0.997
7	rs117667567	143726583	G	T	0.99	7.87e-06	0.01	0.15	-	intergenic_variant	0	0.995
4	rs114088559	90610327	C	T	0.98	8.02e-06	0.02	0.20	RP11-115D19.1	intron_variant, non_coding_transcript_variant	0	0.99
12	rs183935240	127197180	G	A	0.99	8.04e-06	0.00	0.10	-	intergenic_variant	0	0.998
4	rs114487955	16238359	G	A	0.99	8.11e-06	0.00	0.12	TAPT1-AS1	intron_variant, non_coding_transcript_variant	0	0.996
4	rs141146865	66228168	T	A	1.00	8.21e-06	0.00	0.03	EPHA5	intron_variant	0	1
11	rs609423	84705414	T	A	0.01	8.26e-06	8.26	220.30	DLG2, AP000857.2	intron_variant, downstream_gene_variant	0	0.998
9	rs142825270	72022683	T	A	0.97	8.38e-06	0.02	0.22	-	intergenic_variant	0	0.984
11	rs73494848	75159877	C	T	1.00	8.64e-06	0.00	0.07	GDPD5	intron_variant, intron_variant, NMD_transcript_variant, intron_variant, non_coding_transcript_variant	0	0.998
19	chr19:58952327	58952327	A	G	1.00	8.92e-06	0.00	0.04	ZNF132, CTD-2619J13.19, -	upstream_gene_variant, non_coding_transcript_exon_variant, non_coding_transcript_variant, regulatory_region_variant	2	1
2	rs7556839	217167604	A	G	1.00	8.96e-06	0.00	0.07	AC069155.1, MARCH4	upstream_gene_variant, intron_variant	0	1
15	rs117591562	79625213	T	C	0.99	9.02e-06	0.01	0.13	TMED3, -	intron_variant, regulatory_region_variant	0	0.997

Not adjusted to all other outcomes

CHR	SNP	BP	A1	A2	FRQ	P	OR_L95	OR_U95	gene	conseq	genotyped	impute2.info
7	rs17512172	121198154	T	A	0.67	4.94e-07	0.46	0.71	-	intergenic_variant	0	0.997
13	rs9563027	51708406	T	C	0.69	1.58e-06	0.46	0.72	LINC00371	intron_variant, non_coding_transcript_variant	0	0.978
7	rs1469640	42535390	C	T	0.34	1.87e-06	1.36	2.10	-	intergenic_variant	0	0.998
3	rs115155878	114055988	A	C	0.97	2.31e-06	0.12	0.42	ZBTB20	downstream_gene_variant	0	0.991
2	rs7557600	48772202	T	C	0.83	2.74e-06	0.39	0.68	STON1, STON1-GTF2A1L, -	intron_variant, regulatory_region_variant	0	0.991
14	rs150956	70530706	C	G	0.31	3.57e-06	0.42	0.70	SLC8A3	intron_variant, intron_variant, NMD_transcript_variant, upstream_gene_variant	0	0.948
13	rs1974039	48475331	G	A	0.61	5.98e-06	0.49	0.75	LINC00444	downstream_gene_variant	0	0.998
3	rs2661405	21358636	G	C	0.82	7.71e-06	0.44	0.72	-	intergenic_variant	0	0.995
8	rs58527822	134785991	C	T	0.92	9.26e-06	0.30	0.63	CTD-2588J1.1, CTD-2309H9.3	intron_variant, non_coding_transcript_variant, downstream_gene_variant	0	0.971
10	rs7069375	121096445	A	G	0.41	9.52e-06	1.33	2.10	RP11-79M19.2, GRK5, -	upstream_gene_variant, intron_variant, regulatory_region_variant	0	0.916
20	rs200890	1792089	C	T	0.44	1.19e-06	1.33	1.95	RP5-968J1.1	intron_variant, non_coding_transcript_variant	0	0.955
2	rs116266836	155376909	A	G	0.96	4.17e-06	0.19	0.51	-	intergenic_variant	0	0.994
8	rs4732926	29185969	T	C	0.82	5.53e-06	0.47	0.74	DUSP4	downstream_gene_variant	0	0.993
1	rs74081211	68642240	A	G	0.86	6.25e-06	0.41	0.70	WLS, GNG12-AS1	intron_variant, intron_variant, non_coding_transcript_variant, intron_variant, NMD_transcript_variant, upstream_gene_variant	0	0.966
1	rs2761065	30625707	A	G	0.29	8.94e-06	1.28	1.89	-	intergenic_variant	0	0.997
5	rs10051505	105009840	T	C	0.92	5.98e-07	0.15	0.43	-	intergenic_variant	0	0.975
2	rs12614005	65943823	C	G	0.55	1.53e-06	0.34	0.64	AC074391.1	intron_variant, non_coding_transcript_variant	0	0.952
4	chr4:142710442	142710442	C	A	0.70	3.01e-06	1.65	3.38	-	intergenic_variant	2	1
14	rs73591732	27448446	T	C	0.90	3.01e-06	0.23	0.55	RP11-384J4.2	intron_variant, non_coding_transcript_variant	0	0.998
14	rs188623516	46926170	T	A	0.99	4.76e-06	0.00	0.11	LINC00871	intron_variant, non_coding_transcript_variant	0	0.998
17	rs4362428	2090341	C	A	0.60	5.26e-06	0.37	0.67	SMG6	intron_variant	0	0.997
1	rs74634803	156207690	G	C	0.99	6.57e-06	0.03	0.25	PMF1, PMF1-BGLAP, BGLAP	intron_variant, downstream_gene_variant, intron_variant, NMD_transcript_variant, upstream_gene_variant	0	0.993
12	rs117172146	126553783	C	T	0.99	6.74e-06	0.03	0.25	-	intergenic_variant	0	0.994
1	rs180991369	61102621	G	A	0.97	8.53e-06	0.08	0.37	RP11-776H12.1	intron_variant, non_coding_transcript_variant	0	0.987
16	rs9922610	76512728	G	A	0.99	8.57e-06	0.01	0.19	CNTNAP4	intron_variant, non_coding_transcript_variant, intron_variant	0	1

13	rs6650325	95953289	C	A	0.41	9.43e-06	0.32	0.64	ABCC4, -	intron_variant, regulatory_region_variant	0	0.982
4	rs10015432	166855253	T	C	0.90	9.49e-06	0.22	0.55	TLL1	intron_variant, intron_variant, NMD_transcript_variant	0	0.957
11	rs150655300	118731132	A	G	0.99	9.51e-06	0.03	0.26	-	intergenic_variant	0	0.997
8	rs13281094	124741789	T	C	0.64	9.52e-06	0.36	0.67	ANXA13, -	upstream_gene_variant, intron_variant, regulatory_region_variant	0	0.98
19	rs192196148	51983352	G	C	1.00	9.93e-06	0.00	0.09	CEACAM18, -	intron_variant, regulatory_region_variant	0	0.999
8	rs140012666	79131942	G	A	0.99	1.59e-07	0.05	0.25	-	intergenic_variant	0	0.995
6	rs144756202	122373287	A	G	0.98	3.31e-07	0.08	0.32	-	intergenic_variant	0	0.989
8	chr8:49753613	49753613	A	C	0.96	4.65e-07	0.13	0.41	-	intergenic_variant	2	1
7	rs9690969	40541259	T	C	0.82	4.70e-07	0.30	0.59	SUGCT	intron_variant, downstream_gene_variant	0	0.997
3	rs727476	60316417	G	T	0.53	1.53e-06	0.34	0.63	FHIT	intron_variant	0	0.994
12	rs6538067	87650216	G	T	0.12	1.98e-06	1.75	3.80	-	intergenic_variant	0	0.988
16	rs17750019	83277510	A	G	0.90	2.08e-06	0.23	0.55	CDH13	intron_variant, NMD_transcript_variant, intron_variant, intron_variant, non_coding_transcript_variant	0	0.977
7	rs74476624	67956247	G	C	0.97	2.84e-06	0.12	0.41	-	intergenic_variant	0	0.996
6	rs193051711	82737197	C	T	0.99	3.17e-06	0.02	0.20	-	intergenic_variant	0	0.999
18	rs79164706	61879513	G	A	0.97	4.41e-06	0.14	0.45	RP11-909B2.1, RP11-146N18.1	downstream_gene_variant, intron_variant, non_coding_transcript_variant	0	0.997
15	rs191166845	87015139	T	C	0.99	4.52e-06	0.04	0.27	AGBL1	intron_variant	0	0.999
2	rs112604225	53667670	A	G	0.96	4.68e-06	0.15	0.46	-	intergenic_variant	0	0.984
10	rs76136730	125064878	C	G	0.99	5.65e-06	0.04	0.28	-	regulatory_region_variant, intergenic_variant	0	0.996
8	rs72678069	116412922	A	G	0.96	6.08e-06	0.15	0.48	-	intergenic_variant	0	0.99
9	rs4744652	74639028	T	C	0.15	6.24e-06	1.58	3.18	-	intergenic_variant	0	0.995
9	rs4298578	22834741	T	A	0.97	7.69e-06	0.14	0.46	-	intergenic_variant	0	0.995
1	rs115246628	183787321	C	T	1.00	8.03e-06	0.01	0.15	RGL1	intron_variant	0	0.998
3	rs77501676	59290744	T	C	1.00	8.05e-06	0.01	0.14	-	intergenic_variant	0	1
16	rs117092646	85199964	A	G	0.98	8.07e-06	0.10	0.41	CTC-786C10.1	upstream_gene_variant	0	0.996
11	rs2085874	37216930	G	A	0.96	9.93e-06	0.15	0.48	-	intergenic_variant	0	0.989
3	rs75648358	62738642	T	C	0.92	1.61e-07	0.14	0.40	CADPS	intron_variant	0	0.976
11	rs139967808	4503396	G	A	0.99	2.79e-07	0.01	0.14	-	regulatory_region_variant, intergenic_variant	0	0.997
8	rs143497687	1256659	T	C	0.99	3.50e-07	0.01	0.15	-	intergenic_variant	0	0.998
2	rs184936958	224157963	C	T	1.00	3.89e-07	0.00	0.07	-	intergenic_variant	0	0.999
5	rs145487482	56173745	T	C	0.99	4.57e-07	0.01	0.15	MAP3K1	intron_variant	0	0.997
6	rs2432788	5394848	A	G	0.98	4.95e-07	0.06	0.30	FARS2	intron_variant	0	0.999
1	rs72654815	21354625	G	A	0.97	7.00e-07	0.07	0.30	EIF4G3, -	intron_variant, regulatory_region_variant	0	0.987

15	rs6494317	62506361	A	C	0.48	8.28e-07	1.97	4.76	-	intergenic_variant	0	0.939
5	rs145762412	152739188	T	A	0.99	1.96e-06	0.02	0.18	-	intergenic_variant	0	0.997
3	rs11705701	185544309	G	A	0.57	2.00e-06	0.25	0.56	IGF2BP2, -	upstream_gene_variant, regulatory_region_variant	0	0.979
7	rs117208670	77885127	T	C	0.98	2.09e-06	0.05	0.28	MAGI2	3_prime_UTR_variant, intron_variant, non_coding_transcript_variant, intron_variant	0	0.994
1	rs74934992	191255088	G	T	0.97	2.14e-06	0.08	0.36	-	intergenic_variant	0	0.99
12	rs34400042	11746635	G	A	0.83	2.62e-06	0.21	0.53	RP11-434C1.1	downstream_gene_variant	0	0.98
5	rs13153499	11272680	A	G	0.97	2.74e-06	0.09	0.37	CTNND2	intron_variant, intron_variant, NMD_transcript_variant, intron_variant, non_coding_transcript_variant	0	0.994
8	rs189437718	134655419	A	G	0.99	2.91e-06	0.02	0.21	SNORA40	downstream_gene_variant	0	0.996
13	rs8002278	106137154	C	T	0.42	2.94e-06	1.74	3.83	DAOA, DAOA-AS1	intron_variant, NMD_transcript_variant, intron_variant, intron_variant, non_coding_transcript_variant	0	0.99
5	rs189159983	161161144	A	G	0.99	3.29e-06	0.03	0.25	-	intergenic_variant	0	0.996
1	rs76487613	72134192	T	A	0.99	3.47e-06	0.04	0.26	NEGR1, -	intron_variant, regulatory_region_variant	0	0.996
16	rs78064607	71723181	C	T	0.99	3.72e-06	0.01	0.17	PHLPP2	intron_variant, intron_variant, non_coding_transcript_variant, upstream_gene_variant	0	0.997
11	rs7110822	131329729	T	C	0.99	3.77e-06	0.01	0.16	NTM	intron_variant, non_coding_transcript_variant, intron_variant	0	0.997
16	rs140961631	72374645	T	A	0.98	3.84e-06	0.05	0.31	-	intergenic_variant	0	0.996
6	rs58258587	114638966	C	T	0.94	3.86e-06	0.14	0.45	HS3ST5, RP3-399L15.3	intron_variant, intron_variant, non_coding_transcript_variant	0	0.978
5	rs115638059	154003646	G	T	0.99	6.63e-06	0.04	0.27	-	intergenic_variant	0	0.996
10	rs188495776	55081033	G	A	0.99	6.64e-06	0.01	0.18	-	intergenic_variant	0	1
7	rs57149599	37961856	A	G	0.97	6.67e-06	0.07	0.34	EPDR1, SFRP4, -	intron_variant, regulatory_region_variant	0	0.992
12	rs10745552	91469729	T	C	0.03	6.68e-06	2.43	9.43	-	intergenic_variant	0	0.998
1	rs6697616	216636950	A	T	0.44	7.49e-06	1.68	3.72	-	regulatory_region_variant, intergenic_variant	0	0.989
2	rs187863579	183988127	A	G	0.99	7.54e-06	0.03	0.26	NUP35	intron_variant, upstream_gene_variant, downstream_gene_variant	0	0.995
5	rs149963055	152479292	G	A	0.99	7.77e-06	0.02	0.22	AC091969.1	intron_variant, non_coding_transcript_variant	0	0.997
7	rs10244850	43304193	T	C	1.00	8.71e-06	0.00	0.11	HECW1	intron_variant, non_coding_transcript_variant, intron_variant	0	0.998
16	rs8054506	50016562	T	C	0.67	9.33e-06	2.03	6.15	-	intergenic_variant	0	0.895
1	rs11161839	86991815	C	A	0.87	9.35e-06	0.21	0.55	-	intergenic_variant	0	0.98
2	rs146107308	80562716	G	A	0.99	9.36e-06	0.04	0.28	CTNNA2	intron_variant, intron_variant, non_coding_transcript_variant	0	0.998

8	rs143062081	26797906	G	A	0.98	9.41e-06	0.06	0.34	-	intergenic_variant	0	0.988
2	rs150728297	144599994	A	G	0.99	6.01e-08	0.01	0.11	-	regulatory_region_variant, intergenic_variant	0	0.999
5	rs12153771	63106169	G	A	0.95	3.79e-07	0.04	0.23	-	intergenic_variant	0	0.979
7	rs4730800	117373534	A	T	0.99	4.10e-07	0.01	0.10	CTTNBP2	downstream_gene_variant, intron_variant, intron_variant, NMD_transcript_variant	0	0.996
1	rs192540202	237511541	C	G	0.99	4.49e-07	0.01	0.10	RYR2	intron_variant	0	0.996
8	chr8:97892119	97892119	G	A	0.99	4.75e-07	0.01	0.12	CPQ	missense_variant	2	1
18	rs140914711	41414229	T	C	0.98	6.56e-07	0.02	0.19	RNU6-443P	upstream_gene_variant	0	0.993
11	rs146495243	64886310	C	T	0.99	6.69e-07	0.02	0.16	MRPL49, FAU, TM7SF2, ZN-HIT2, SYVN1, AP003068.12	upstream_gene_variant, downstream_gene_variant	0	0.998
12	rs144415411	90377606	G	A	1.00	1.14e-06	0.00	0.09	RP11-654D12.2	intron_variant, non_coding_transcript_variant	0	0.999
1	rs188095292	246892698	C	T	0.99	1.26e-06	0.02	0.19	SCCPDH, -	intron_variant, regulatory_region_variant	0	0.995
3	rs6788430	42505962	T	C	0.99	1.36e-06	0.01	0.11	-	intergenic_variant	0	0.998
7	rs12670163	55293011	A	G	1.00	1.55e-06	0.01	0.11	EGFR	intron_variant	0	0.999
2	rs72868355	37048367	C	G	0.99	1.70e-06	0.02	0.20	-	intergenic_variant	0	0.998
1	rs116255984	246634974	G	A	0.99	1.71e-06	0.01	0.13	SMYD3	intron_variant, non_coding_transcript_variant, in-tron_variant	0	0.997
1	rs140789420	169845576	C	T	1.00	1.87e-06	0.00	0.08	SCYL3	intron_variant, intron_variant, non_coding_transcript_variant	0	0.999
4	rs114088559	90610327	C	T	0.98	1.98e-06	0.02	0.21	RP11-115D19.1	intron_variant, non_coding_transcript_variant	0	0.99
2	rs2163050	41826738	G	A	0.85	2.18e-06	0.11	0.39	AC010739.1	downstream_gene_variant	0	0.989
11	rs189508277	32271897	G	A	0.99	2.20e-06	0.01	0.15	RP1-65P5.1	intron_variant, non_coding_transcript_variant	0	0.995
12	rs181634788	90788710	C	T	0.99	2.51e-06	0.00	0.10	-	intergenic_variant	0	0.997
20	rs76449538	24111259	T	C	0.98	2.51e-06	0.03	0.23	-	intergenic_variant	0	0.998
6	rs13213508	6152555	G	A	0.94	2.55e-06	0.08	0.35	F13A1	intron_variant	0	0.999
7	rs7794878	78080244	A	G	0.86	2.66e-06	0.12	0.41	MAGI2, -	intron_variant, intron_variant, non_coding_transcript_variant, regulatory_region_variant	0	0.999
8	rs139134207	133066750	C	T	1.00	2.71e-06	0.00	0.08	OC90	intron_variant	0	1
8	rs189901754	132632117	G	A	1.00	2.90e-06	0.00	0.08	-	intergenic_variant	0	0.998
19	rs147472919	8408802	A	G	0.97	3.38e-06	0.04	0.26	KANK3, CTD-2550O8.7, AC010323.1	upstream_gene_variant, downstream_gene_variant	0	0.989
9	rs10811524	21283847	T	A	0.80	4.45e-06	0.11	0.41	-	intergenic_variant	0	0.975

3	rs182573978	108804875	G	A	1.00	4.56e-06	0.00	0.12	MORC1	intron_variant	0	0.998
12	rs117624293	95438785	G	C	0.95	4.74e-06	0.06	0.33	NR2C1	upstream_gene_variant, downstream_gene_variant, intron_variant, intron_variant, non_coding_transcript_variant, intron_variant, NMD_transcript_variant	0	0.985
1	rs182819833	226681975	C	A	1.00	5.03e-06	0.00	0.09	CDKN2AIPNLP1	upstream_gene_variant	0	1
3	rs181832941	189567428	T	C	1.00	5.36e-06	0.01	0.13	TP63	intron_variant, intron_variant, non_coding_transcript_variant	0	0.998
14	rs143247799	25100109	C	T	0.99	5.51e-06	0.00	0.11	RP11-104E19.1, GZMB, -	intron_variant, non_coding_transcript_variant, downstream_gene_variant, regulatory_region_variant	0	0.998
4	rs73210745	5942961	A	C	0.79	5.95e-06	0.10	0.40	-	intergenic_variant	0	0.94
11	rs11033029	35240076	A	C	0.87	6.01e-06	0.10	0.40	RP1-68D18.2, CD44, RP1-68D18.4, -	downstream_gene_variant, intron_variant, intron_variant, non_coding_transcript_variant, upstream_gene_variant, intron_variant, NMD_transcript_variant, regulatory_region_variant	0	0.977
9	rs191216752	111308811	C	G	1.00	6.01e-06	0.00	0.11	-	intergenic_variant	0	0.998
7	rs79995619	33541130	A	G	0.99	6.48e-06	0.01	0.18	BBS9, -	intron_variant, upstream_gene_variant, intron_variant, NMD_transcript_variant, regulatory_region_variant	0	0.996
14	rs188208602	98385236	C	T	0.99	6.74e-06	0.01	0.13	-	intergenic_variant	0	0.999
4	rs148632338	156047536	C	T	0.99	7.39e-06	0.01	0.16	-	intergenic_variant	0	0.997
8	rs187288337	58939051	C	T	0.99	7.43e-06	0.01	0.13	FAM110B, RP11-1112C15.2	intron_variant, non_coding_transcript_variant, intron_variant, downstream_gene_variant	0	0.998
12	rs187329208	128727850	C	T	1.00	7.66e-06	0.00	0.01	MIR4419B	upstream_gene_variant	0	1
10	rs184497072	23252404	A	T	1.00	7.67e-06	0.00	0.05	ARMC3	intron_variant, non_coding_transcript_variant, downstream_gene_variant, intron_variant	0	0.999
14	chr14:24799164	24799164	G	T	1.00	7.77e-06	0.00	0.12	ADCY4, RP11-934B9.3, -	upstream_gene_variant, missense_variant, downstream_gene_variant, non_coding_transcript_exon_variant, non_coding_transcript_variant, missense_variant, NMD_transcript_variant, regulatory_region_variant	2	1
9	rs72733349	102987266	A	C	0.83	7.80e-06	0.10	0.41	INVS	intron_variant, non_coding_transcript_variant, intron_variant	0	0.961
12	rs183138047	51548306	A	C	1.00	8.49e-06	0.00	0.13	TFCP2, Y_RNA	intron_variant, downstream_gene_variant	0	0.999
1	rs12136490	230864486	G	T	0.98	8.82e-06	0.03	0.25	RP11-99J16__A.2, RN7SL467P, -	intron_variant, non_coding_transcript_variant, upstream_gene_variant, regulatory_region_variant	0	0.998

3	rs180782090	178167604	G	A	1.00	8.83e-06	0.00	0.09	LINC01014, KC-NMB2	intron_variant, non_coding_transcript_variant, intron_variant	0	0.999
5	rs2471068	38104956	G	A	0.06	8.93e-06	2.96	16.26	CTD-2116N24.1, -	intron_variant, non_coding_transcript_variant, regulatory_region_variant	0	0.975
7	rs191150966	69983852	G	C	1.00	9.34e-06	0.00	0.11	AUTS2	intron_variant	0	0.999
3	rs75169866	15819505	T	C	0.97	9.45e-06	0.04	0.29	ANKRD28	downstream_gene_variant, intron_variant, NMD_transcript_variant, intron_variant, intron_variant, non_coding_transcript_variant	0	0.989
10	rs117553785	71209051	G	A	0.98	9.53e-06	0.03	0.25	TSPAN15	upstream_gene_variant	0	0.987
